# Proteostasis is differentially modulated by inhibition of translation initiation or elongation

**DOI:** 10.7554/eLife.76465

**Published:** 2023-10-05

**Authors:** Khalyd J Clay, Yongzhi Yang, Christina Clark, Michael Petrascheck

**Affiliations:** 1 Department of Molecular Medicine, Department of Neuroscience, Scripps Research Institute La Jolla United States; Buck Institute for Research on Aging United States; Columbia University United States

**Keywords:** aging, protein folding, mRNA translation, protein synthesis, protein aggregation, longevity, *C. elegans*

## Abstract

Recent work has revealed an increasingly important role for mRNA translation in maintaining proteostasis. Here, we use chemical inhibitors targeting discrete steps of translation to compare how lowering the concentration of all or only translation initiation-dependent proteins rescues *Caenorhabditis elegans* from proteotoxic stress. We systematically challenge proteostasis and show that pharmacologically inhibiting translation initiation or elongation elicits a distinct protective profile. Inhibiting elongation protects from heat and proteasome dysfunction independently from HSF-1 but does not protect from age-associated protein aggregation. Conversely, inhibition of initiation protects from heat and age-associated protein aggregation and increases lifespan, dependent on *hsf-1*, but does not protect from proteotoxicity caused by proteasome dysfunction. Surprisingly, we find that the ability of the translation initiation machinery to control the concentration of newly synthesized proteins depends on HSF-1. Inhibition of translation initiation in wild-type animals reduces the concentration of newly synthesized proteins but increases it in *hsf-1* mutants. Our findings suggest that the HSF-1 pathway is not only a downstream target of translation but also directly cooperates with the translation initiation machinery to control the concentration of newly synthesized proteins to restore proteostasis.

## Introduction

Protein synthesis is a highly regulated process involving the precise orchestration of many chaperones, co-factors, enzymes, and biomolecular building blocks. It is critical at every level of the life cycle, from development through aging, and is central to stress adaptation. Protein synthesis largely determines the folding load on the proteostasis network, which regulates protein production, folding, trafficking, and degradation to maintain a functional proteome. The imbalance of the proteostasis network caused by age-associated stress or acute environmental insults leads to misfolding of proteins, accumulation of aggregates, and eventually to disease (Balch et al., 2008).

A substantial body of work has revealed that the protein synthesis machinery directly participates in protein folding or aggregation. For example, in mice, point mutations in specific tRNAs or components of the ribosomal quality control pathway can lead to protein aggregation and neurodegeneration (Vo et al., 2018; Yonashiro et al., 2016; Chu et al., 2009; Nollen et al., 2004; Nedialkova and Leidel, 2015). Similarly, early RNAi screens in the nematode *Caenorhabditis elegans* identified several ribosomal subunits whose knockdown increased the aggregation of polyglutamine (PolyQ) proteins (Nollen et al., 2004). These findings highlight the importance of translation in protein misfolding.

However, subsequent studies reveal a more intricate role of translation in protein aggregation. Depending on how translation is modulated, it leads to either decreased or increased protein aggregation. For example, RNAi-mediated knockdown of translation initiation factors increases lifespan, improves proteostasis, and reduces protein aggregation in *C. elegans* (Balch et al., 2008; Rogers et al., 2011; McQuary et al., 2016; Lan et al., 2019; Howard et al., 2016). Similarly, work in yeast and cell culture shows that pharmacological inhibition of translation prior to a heat shock prevents proteins from aggregating (Medicherla and Goldberg, 2008; Choe et al., 2016; Xu et al., 2016; Riback et al., 2017). While studies in *C. elegans*, cell culture, and yeast agree that inhibition of translation reduces protein aggregation, their proposed underlying mechanisms differ. Overall, the proposed mechanisms can be categorized into two broad models on how lowering translation reduces protein aggregation.

The first model, referred to as the *selective translation model*, proposes that inhibition of translation is selective. In the selective translation model, the increased availability of ribosomes leads to differential translation of mRNAs coding for stress response factors and thus to increased folding capacity (Rogers et al., 2011; McQuary et al., 2016; Lan et al., 2019; Seo et al., 2013). In general, studies proposing a version of *the selective translation* model show that inhibiting translation requires HSF-1 to reduce protein aggregation (Howard et al., 2016; Tye and Churchman, 2021). In the *selective translation model*, protein aggregation is reduced by an HSF-1-dependent active generation of folding capacity to remodel the proteome.

The second model, referred to as the *reduced folding load model,* proposes that newly synthesized proteins are the primary aggregation-prone species of proteins. Therefore, newly synthesized proteins constitute the most significant folding load on the proteostasis machinery. Inhibition of translation reduces the concentration of newly synthesized proteins and, thus, the load on the folding machinery. In contrast to the *selective translation model*, which proposes selective protein synthesis of HSF-1-dependent stress response factors, the *reduced folding load model* does not depend on specific factors but generates folding capacity by reducing the overall folding load. A problem comparing previous studies has been their use of different model organisms, different proteostatic insults, and modes of inhibition. Furthermore, previous studies did not control how much protein synthesis was reduced by different modes of translation inhibition making direct comparisons between the *selective translation model* and the *reduced folding load model* difficult.

In this study, we systematically compared these two models in *C. elegans* using pharmacological agents to block various steps along the protein synthesis cycle but ensured to use inhibitors that achieve comparable reductions in the concentration of newly synthesized proteins across the different interventions. We characterize how lowering translation protects *C. elegans* from proteotoxic insults such as proteasome inactivation, heat shock, and aging. Our data reveal that the step inhibited in mRNA translation dictates which of the two protective mechanisms is activated. Furthermore, they uncover an unexpected link between translation initiation and HSF-1 and show that inhibition of translation initiation fails to lower the concentration of newly synthesized proteins in the absence of HSF-1.

## Results

### Characterizing mRNA translation inhibitors in *C. elegans*

**Figure 1.**
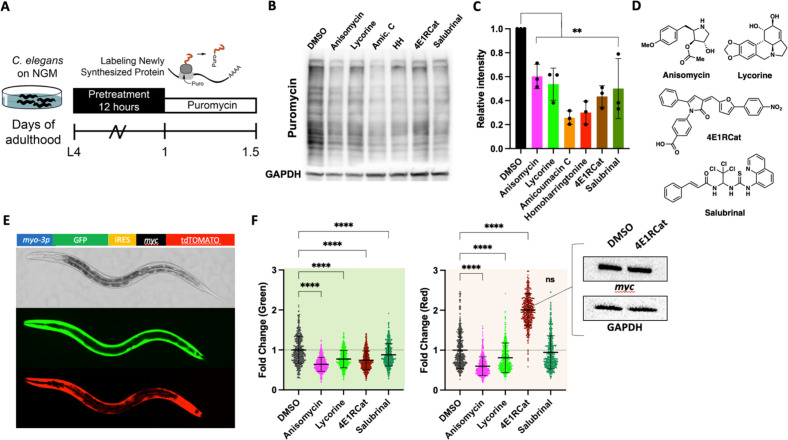
Identifying mechanistic inhibitors of protein synthesis in *C. elegans.* (**A**) Monitoring changes in protein synthesis using the SUrface SEnsing of Translation (SUnSET) method. *C. elegans* were treated with solvent (DMSO) or the indicated inhibitors (100 μM) for 12 hr, followed by a 4 hr puromycin incorporation. Worms were lysed, and protein extracts were run on SDS-PAGE gels, followed by staining with a puromycin-specific antibody. (**B**) Six translation inhibitors reduce puromycin incorporation relative to DMSO control. GAPDH was used as a loading control. (**C**) Quantification of three independent SUnSET experiments, as shown in (**B**). Significance was determined by one-way ANOVA with Dunnett’s multiple comparisons tests where **=p ≤ 0.01 for all treatments. Error bars indicate mean ± SD from three independent trials. (**D**) Chemical structures of anisomycin, lycorine, 4E1RCat, and salubrinal. Note each is structurally distinct. (**E**) Representative pictures showing the expression pattern of the bi-cistronic reporter in L4 stage animals. The image in the brightfield channel shows an L4 stage animal. Images in green and red channels show that GFP and tdtomato are expressed in body wall muscle. (**F**) Fluorescence of 500 transgenic animals treated with anisomycin, lycorine, 4E1RCat, and salubrinal. Each treatment reduced the GFP signal (green shading), but only anisomycin and lycorine reduced the dtTomato signal (red shading). Significance was determined by one-way ANOVA with Dunnett’s multiple comparisons tests where ****=p ≤ 0.0001 for all treatments. Error bars indicate mean ± SD. The experiment was repeated four times with similar results. Inset: 4E1RCat emits red fluorescence; therefore, the expression of tdTomato needed to be tested by western blot. 4E1Rcat does not change myc expression on the protein level, GAPDH is used as a loading control, a representative image of three independent experiments. Figure 1—source data 1.Unedited SUrface SEnsing of Translation (SUnSET) western blots. Figure 1—source data 2.Quantifications of western blots. Figure 1—source data 3.ChemDraw files for structures. Figure 1—source data 4.Summary of green/red integrated fluorescence and unedited myc/GAPDH blots.

We first set out to identify suitable pharmacological translation initiation (TI) and translation elongation (TE) inhibitors by screening a series of translation inhibitors for their ability to lower protein synthesis in *C. elegans* in a dose-dependent manner (Dmitriev et al., 2020). To monitor protein synthesis, we employed SUrface SEnsing of Translation (SUnSET) (Arnold et al., 2014). In this method, translating ribosomes incorporate puromycin into newly synthesized proteins. The level of puromycin incorporation serves as a quantitative measure for translation and is detected by western blotting using an anti-puromycin monoclonal antibody (Figure 1A). The six molecules examined all reduced puromycin incorporation relative to DMSO controls to varying degrees (Figure 1B and C). Based on their ability to lower the concentration of newly synthesized proteins by ~40–50% and their annotated targets (vide infra), we selected four structurally distinct inhibitors, anisomycin, lycorine, 4E1RCat, and salubrinal, to investigate further (Figure 1D).

**Figure 1—figure supplement 1.**
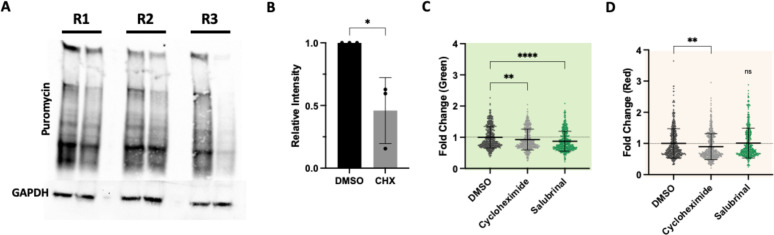
Cycloheximide, a ubiquitously used eukaryotic translation inhibitor, inhibits translation elongation (TE) in *C. elegans.* (**A**) Cycloheximide (100 μΜ) unreliably reduces puromycin incorporation relative to DMSO control in N2 animals. All three experiments were conducted under identical conditions and run on the same blot to reduce technical batch effects but resulted in unreliable reductions of newly synthesized proteins. (**B**) Quantification of three independent SUrface SEnsing of Translation (SUnSET) experiments. Data are displayed as mean ± SEM and *=p ≤ 0.05 by two-tailed Student’s t-test. Note the large error bars for cycloheximide treatment that led us to exclude it from the study, as cycloheximide does not allow precise control over the concentration of newly synthesized proteins. We note the p-value is higher than when anisomycin or lycorine are used. (**C**) Replicate experiment as in Figure 1F with cycloheximide and salubrinal. Each treatment reduced the GFP signal. Significance was determined by one-way ANOVA with Dunnett’s multiple comparisons tests where **=p ≤ 0.01 and ****=p ≤ 0.0001. Error bars indicate mean ± SD. The experiment was repeated three times with similar results. We note the p-value was less significant for cycloheximide than for the same experiment using anisomycin or lycorine instead. (**D**) Replicate experiment as in Figure 1F with cycloheximide and salubrinal. Only the TE inhibitor cycloheximide reduced tdTomato signal while the initiation inhibitor salubrinal did not. Significance was determined by one-way ANOVA with Dunnett’s multiple comparisons tests where **=p ≤ 0.01. Error bars indicate mean ± SD. The experiment was repeated three times with similar results. We note the p-value was less significant for cycloheximide than for the same experiment using anisomycin or lycorine instead. Figure 1—figure supplement 1—source data 1.Unedited SUrface SEnsing of Translation (SUnSET) western blots. Figure 1—figure supplement 1—source data 2.Quantifications of western blots. Figure 1—figure supplement 1—source data 3.Summary of green integrated fluorescence. Figure 1—figure supplement 1—source data 4.Summary of red integrated fluorescence.

The selected molecules have been extensively studied in yeast and cell culture to identify their purported mechanism of action—broadly, anisomycin and lycorine inhibit TE, while 4E1RCat and salubrinal inhibit TI. To confirm their action as TI or TE inhibitors in *C. elegans*, we generated a bi-cistronic reporter to co-express both GFP and tdTomato under the control of the *myo-3* promotor and an internal ribosomal entry site (IRES) (Figure 1E; Li and Wang, 2012). In transgenic worms carrying the *myo3p::*GFP-IRES*-*tdTomato construct, strong GFP and tdTomato fluorescence were observed primarily in the muscle tissue. Using this reporter, we measured the fluorescence of 500 L4 animals treated with one of the four translation inhibitors. As expected for the TE inhibitors, anisomycin and lycorine decreased fluorescence in both green and red channels.

On the other hand, the TI inhibitors 4E1RCat and salubrinal only decreased the fluorescence of the green channel, consistent with an initiation-dependent translation of the GFP mRNA sequence and the initiation-independent translation of the IRES-tdTomato sequence (Figure 1F). However, we observed a strong increase in red fluorescence in worms treated with 4E1RCat, but this is due to the red color of the molecule itself. We confirmed no change in tdTomato levels by western blot (Figure 1F, inset). The above results confirm that the four translation inhibitors function in distinct translation steps, consistent with previous reports in yeast and cell culture.

We also confirmed the mechanism of translation inhibition of cycloheximide. Although cycloheximide is a widely used inhibitor of translation throughout the literature, to our knowledge, it has not been shown to specifically act as a TE inhibitor in *C. elegans*. Using the SUnSET assay and our bi-cistronic reporter, we show that cycloheximide directly reduces the concentration of newly synthesized proteins (Figure 1—figure supplement 1A, B) and functions as a TE inhibitor since we observed a reduction of both the GFP and the tdTomato signal. Salubrinal, which we included as a TI inhibitor control, only reduced translation promoted by *myo-3* but not the IRES site as seen before (Figure 1—figure supplement 1C, D). We noted that the inhibition of translation by cycloheximide across different trials was considerably more variable compared to anisomycin and lycorine (compare Figure 1B and C with Figure 1—figure supplement 1A, B). We therefore decided to proceed with anisomycin and lycorine as TE inhibitors as they reliably reduced the concentration of newly synthesized proteins by 40–50%.

### Initiation and elongation inhibitors protect from thermal stress by HSF-1-dependent and -independent mechanisms, respectively

**Figure 2.**
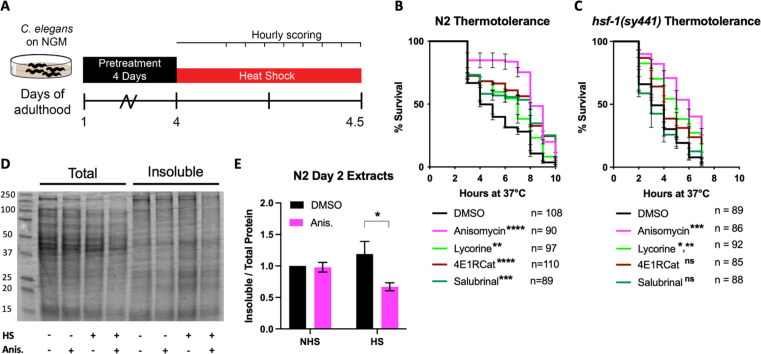
Initiation but not elongation inhibitors depend on HSF-1 to protect *C.* *elegans* from thermal stress. (**A**) Day 1 adult wild-type (**N2**) and *hsf-1(sy441)* animals were treated for 3 days, then transferred to NGM plates. They were then subjected to a constant, non-permissive temperature of 36°C (heat shock [HS]) and scored alive/dead every hour by movement. (**B**) Graph shows survival as a function of hours at 36°C of day 4 adult N2 animals pre-treated with 100 μM translation inhibitor. Data show the mean ± SEM from three independent trials where each measurement is at least: **=p ≤ 0.01, ***p≤0.001, and ****p≤0.0001 by row-matched two-way ANOVA with Šídák multiple comparisons test. (**C**) Graph shows survival as a function of hours at 36°C of day 4 adult *hsf-1(sy441)* animals pre-treated with 100 μM translation inhibitor. Data show the mean ± SEM from three independent trials where each measurement is at least: *=p ≤ 0.05, **=p ≤ 0.01, and ***p≤0.001 by row-matched two-way ANOVA with Šídák multiple comparisons test. (**D**) Representative SDS-PAGE gel stained with the protein stain Coomassie blue for visualization. Anisomycin (Anis.) reduces the proportion of detergent-insoluble protein following a 2 hr HS of N2 animals. Proteins were detergent extracted, ultracentrifuged, and the insoluble pellet was resuspended in 8 M urea before running on the gel. (**E**) Quantification of four separate extractions shows anisomycin significantly reduces HS-induced aggregation in wild-type N2 animals. Gels were stained with Sypro Ruby. Data are displayed as mean ± SEM and *=p ≤ 0.05 by two-tailed Student’s t-test. Figure 2—source data 1.Summary of thermotolerance survival numbers (**N2**). Figure 2—source data 2.Summary of thermotolerance survival numbers (*hsf-1(sy441)*)*.* Figure 2—source data 3.Unedited SDS-PAGE gel. Figure 2—source data 4.Quantifications of insoluble gels.

After confirming that the four translation inhibitors (Figure 1D) acted as TI or TE inhibitors in *C. elegans* as seen in mammals, we set out to investigate how pharmacologically targeting different steps of the translation cycle will affect stress-induced protein aggregation. We first chose thermal stress to induce protein aggregation and asked if both TI and TE inhibitors improve stress resistance. The animals were treated with either of the four inhibitors on day 1 of adulthood and, after a 72 hr treatment, moved to a non-permissive temperature of 36°C (Figure 2A). Hourly monitoring revealed that all four molecules significantly improved the survival of N2 animals (Figure 2B). These data show that both the inhibition of TI and TE protect from thermal stress.

We next asked if translation inhibitors require the canonical heat shock response (HSR) controlled by the transcription factor HSF-1 to protect from heat stress (HS). Previous work by us and others resulted in contradictory findings on whether protection from heat by translation inhibition depends on HSF-1 (Seo et al., 2013; Solis et al., 2018; Zhou et al., 2014). Therefore, to test if translation inhibition protects from HS in an HSF-1-dependent or -independent manner, we repeated the thermotolerance assay in HSR-deficient *hsf-1(sy441)* mutants. Only the TE inhibitors anisomycin and lycorine protected *hsf-1(sy441)* from HS-induced proteotoxicity, while the TI inhibitors 4E1RCat and salubrinal did not (Figure 2C). These results showed that different modes of translational inhibition protect by genetically separable mechanisms. However, this also reconciles previous contradictions as different groups inhibited translation using inhibitors or RNAi with specificity for either step.

To broadly confirm the ability of TE inhibitors to protect from thermal proteotoxicity through improved proteostasis, we conducted sequential detergent extractions to biochemically isolate and quantify soluble and insoluble proteins in wild-type (N2) animals (David et al., 2010; Reis-Rodrigues et al., 2012; Simonsen et al., 2008). Following a 2 hr HS at 36°C, we observed a substantial increase in insoluble protein compared to non-heat-shocked controls. Furthermore, pre-treatment with the TE inhibitor anisomycin suppressed the increase in protein insolubility (Figure 2D and E), consistent with the observed thermal protection. These results reveal that TI inhibitors trigger an HSR-dependent mechanism to protect from thermal stress, while TE inhibitors trigger an HSR-independent mechanism. We concluded that different modes of translation inhibition protect the proteome by genetically distinct mechanisms.

### Translation elongation but not initiation inhibitors protect from heat shock-induced PolyQ aggregation

**Figure 3.**
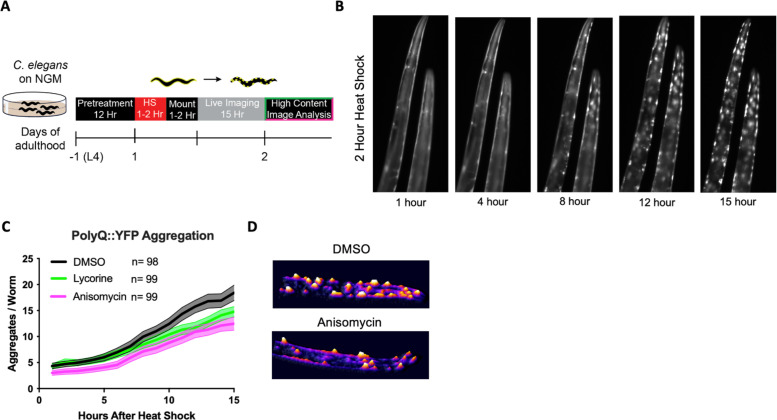
Elongation inhibitors reduce the number of heat shock-induced protein aggregates. (**A**) Day 1 AM140 adult worms expressing the polyglutamine-YFP fusion protein (PolyQ::YFP) in their muscle were subjected to heat stress (HS) on NGM plates for 2 hr at 36°C followed by a 1–2 hr mounting/immobilization procedure in 384-well plates and subsequent live imaging for 15 hr. (**B**) Fluorescent time-lapse images of two animals expressing the PolyQ::YFP fusion protein in the body wall muscle. The animals were embedded in the hydrogel for immobilization. Following a 2 hr HS, animals were imaged over 15 hr; by 8 hr, the YFP signal began to localize into discrete puncta that persisted through the observation time. (**C**) Graph shows the mean number of PolyQ aggregates per worm as a function of time following heat shock. *C. elegans* (PolyQ::YFP) were pre-treated with lycorine, anisomycin (100 μΜ), or DMSO. Lines indicate mean, and shading indicates 95% CI. (**D**) Representative images of control (top) and 100 μΜ anisomycin-treated (bottom) PolyQ animals 15 hr after HS. The representative images shown have been uniformly modified using the ‘3D Surface Plot’ plugin in ImageJ to visualize aggregates. Figure 3—source data 1.Uncropped time-lapse fluorescence micrographs. Figure 3—source data 2.Summary of aggregation numbers. Figure 3—source data 3.Video of aggregation process with ‘3D Surface Plot’ plugin.

TE inhibitors are reported to free up folding capacity by reducing overall protein synthesis. To monitor folding capacity in live imaging, we developed an experimental procedure to examine how translation inhibition will dynamically affect protein aggregation in real time in the PolyQ::YFP strain AM140 (Figure 3A). The AM140 strain expresses a stretch of 35 glutamine residues fused to YFP (PolyQ::YFP) in the muscle, which we used as an orthogonal model of reduced protein folding capacity. Expression of the aggregation-prone PolyQ stretch increases the protein folding load on the proteostasis system. Thus, its aggregation propensity acts as a sensor for protein folding capacity (Brignull et al., 2006; Moronetti Mazzeo et al., 2012). After a 2 hr HS, the initially diffuse PolyQ signal gradually localized into puncta (Figure 3B, Video 1). Aggregation foci formation resulted from a redistribution of the YFP signal into aggregation foci as the total level of YFP fluorescent signal for a given animal remained constant (Figure 3—figure supplement 1A).

**Figure 3—figure supplement 1.**
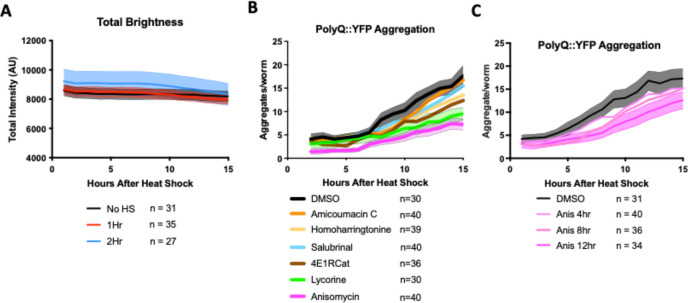
Requirements for protection from heat shock-induced polyglutamine (PolyQ) aggregation. (**A**) Total fluorescent YFP intensity in PolyQ transgenic animals does not change significantly within the 15 hr of imaging of the animals after the heat stress (HS), showing that the aggregate formation is a redistribution of soluble PolyQ::YFP into aggregation foci. Lines indicate mean, and shading indicates 95% CI. (**B**) Transgenic AM140 worms treated with indicated chemicals for 12 hr at the late L4 stage, then subjected to 36°C for 2 hr on day 1 of adulthood. Anisomycin and lycorine had suppressed aggregate formation as measured in our live imaging protocol. In addition, there was a non-significant tendency for 4ER1Cat. Lines indicate mean, and shading indicates 95% CI for DMSO, anisomycin, and lycorine. (**C**) 12 hr pre-treatment of anisomycin in PolyQ *C. elegans* was necessary to inhibit aggregation significantly. Lines indicate means, while DMSO and 12 hr treatment with anisomycin (100 μΜ) includes 95% CI as indicated by shading. Figure 3—figure supplement 1—source data 1.Quantification of heat shock time dependency. Figure 3—figure supplement 1—source data 2.Quantification of translation inhibitor screen HS-induced aggregation. Figure 3—figure supplement 1—source data 3.Quantification of time dependency for inhibitor treatment.

**Video 1.** Anisomycin prevents heat shock-induced polyglutamine (PolyQ) aggregation. AM130 *C. elegans* expressing 35 glutamine residues fused to YFP (PolyQ::YFP) were treated with DMSO or anisomycin for 12 hr before subjecting the animals to a 2 hr heat shock at 36°C. The animals were embedded into a hydrogel physically immobilizing the worm and imaged over 15 hr. The representative images shown have been uniformly modified using the ‘3D Surface Plot’ plugin in ImageJ to visualize aggregates.

Only TE inhibitors lycorine and anisomycin significantly reduced HS-induced PolyQ aggregation, while the TI inhibitors 4E1RCat had a small effect and salubrinal had no effect (Figure 3C, Figure 3—figure supplement 1B). Pre-treatment with anisomycin and lycorine reduced the number of PolyQ aggregates per worm. However, pre-treatment did not change the onset of aggregation, the rate of formation, or the final size of the aggregation foci (Morley et al., 2002; Figure 3D). Furthermore, we found the effect of anisomycin or lycorine to be time-dependent. They strongly inhibited aggregation following a 12 hr preincubation period but less so following a 4 hr preincubation (Figure 3—figure supplement 1C). Starting treatment with anisomycin after the HS did not reduce the number of aggregation foci (not shown). These results suggest that anisomycin and lycorine reduce the early formation of aggregate foci but do not alter the dynamics once aggregation begins.

### Translation elongation but not initiation inhibitors protect from proteasome dysfunction

The data thus far suggest that inhibition of translation protects from folding stress by an HSF-1-dependent and -independent mechanism determined by the inhibited translation step. The proteasomal system is another critical proteostasis mechanism by which cells clear protein aggregates. The proteasomal system ubiquitinylates misfolded proteins by ubiquitin ligases to target them for degradation by the 26S proteasome. Blocking proteasome degradation by bortezomib, a specific inhibitor of the 20S subunit, results in the formation of protein aggregates and proteotoxic stress (Schrader et al., 2016).

**Figure 4.**
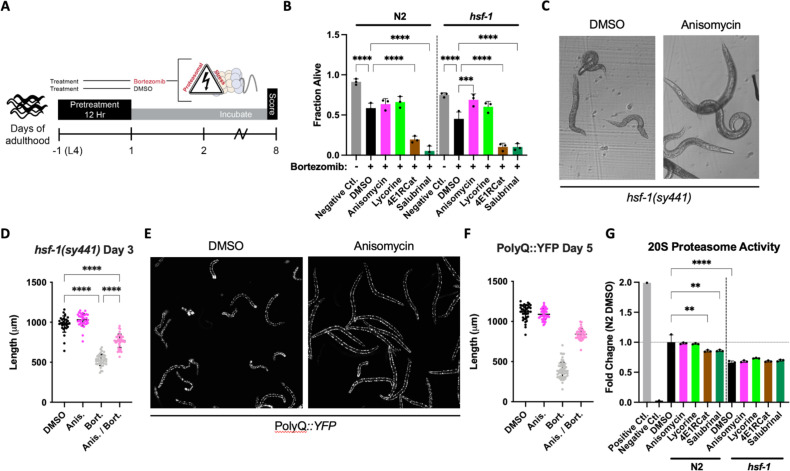
Elongation inhibitors protect *C.* *elegans* from proteasomal stress independent of *hsf-1.* (**A**) Worms were pre-treated for 12 hr with DMSO or indicated inhibitors, followed by bortezomib (75 μM) treatment. The animals were then incubated with the combined treatment for 8 days and scored as alive/dead based on movement. (**B**) TE inhibitor treatment improved morphological features (not shown) and provided limited protection from bortezomib-induced proteotoxicity in N2 animals. TI inhibitor treatment enhanced toxicity. In *hsf-1(sy441)* animals, TE inhibitors protected from bortezomib-induced proteotoxicity, while TI inhibitors continued to sensitize worms to proteotoxicity. Data are displayed as mean ± SD and ****=p < 0.0001 by one-way ANOVA with Dunnet’s multiple comparisons test. Total of three independent experiments. (**C**) Representative brightfield images of day 3 *hsf-1(sy441)* animals show anisomycin pre-treatment prevented the *sma* phenotype observed to be caused by proteasomal inhibition. (**D**) Measured length of *hsf-1(sy441)* worms at day 3 of adulthood. Anisomycin treatment almost completely rescued the *sma* phenotype induced by bortezomib. Data are displayed as mean ± SD and ****=p < 0.0001 by a one-way ANOVA with Šídák multiple comparisons test. 30–42 animals per condition. Total of three independent experiments. (**E**) Representative fluorescent images of PolyQ worms treated with anisomycin at day 5. Bortezomib treatment caused morphological defects in animals (left panel), and anisomycin pre-treatment prevented these pathological defects (right panel). (**F**) Measured length of PolyQ worms at day 5 of adulthood. Anisomycin treatment almost entirely rescued the small (*sma*) phenotype induced by bortezomib. Data are displayed as mean ± SD and ****=p < 0.0001 by one-way ANOVA with Dunnet’s multiple comparisons test. 42–57 animals per condition. Total of three independent experiments. (**G**) TI inhibitor treatment significantly reduced 20S proteasomal activity in N2 lysate. Compared to the wild-type, the proteasomal activity was lower in *hsf-1(sy441)* mutants, but TI inhibitors did not further reduce it. Positive control: 5 µL of 20S proteasome positive control (Chemicon Part No. 90205). Negative control: N2 lysate treated with 25 μM lactacystin, a 20S proteasome inhibitor (Chemicon Part No. 90208). Data are displayed as mean ± SD where **=p < 0.01 and ****=p < 0.0001 by one-way ANOVA with Šídák multiple comparisons test. Three biological replicates. Figure 4—source data 1.Quantification of survival of N2 and *hsf-1(sy441)* treated with bortezomib. Figure 4—source data 2.Uncropped brightfield micrographs. Figure 4—source data 3.Quantification of *hsf-1(sy441)* length. Figure 4—source data 4.Uncropped fluorescence micrographs. Figure 4—source data 5.Quantification of polyglutamine (PolyQ) length. Figure 4—source data 6.Quantification of 20S proteasome assay.

We pre-treated both N2 and *hsf-1(sy441)* L4 animals with either TI or TE inhibitors for 12 hr, followed by the addition of bortezomib, and measured survival on day 8 of adulthood (Figure 4A). We chose to include the *hsf-1(sy441)* background because it serves as a proxy of reduced folding capacity given the defective HSR, allowing us to further investigate the relationship between reduced translation and its conditional dependency on *hsf-1*. Compared to non-treated controls, bortezomib decreased the survival of both genotypes. TE inhibitors improved the survival of the *hsf-1(sy441)* animal back to untreated levels but only showed a non-significant tendency to improve the survival of N2 wild-type animals (Figure 4B). In contrast, TI inhibitors enhanced the proteotoxicity elicited by bortezomib in both backgrounds. We also observed bortezomib-treated *hsf-1(sy441)* animals to shrink in size (*sma* phenotype) (Figure 4C). We quantified this effect after a 12 hr pre-treatment of anisomycin and successive co-incubation of bortezomib at day 3 of adulthood. Anisomycin substantially rescued *sma* phenotypes, almost completely reversing the animals back to normal size (Figure 4D). A similar rescue was observed for lycorine but not quantified.

**Figure 4—figure supplement 1.**
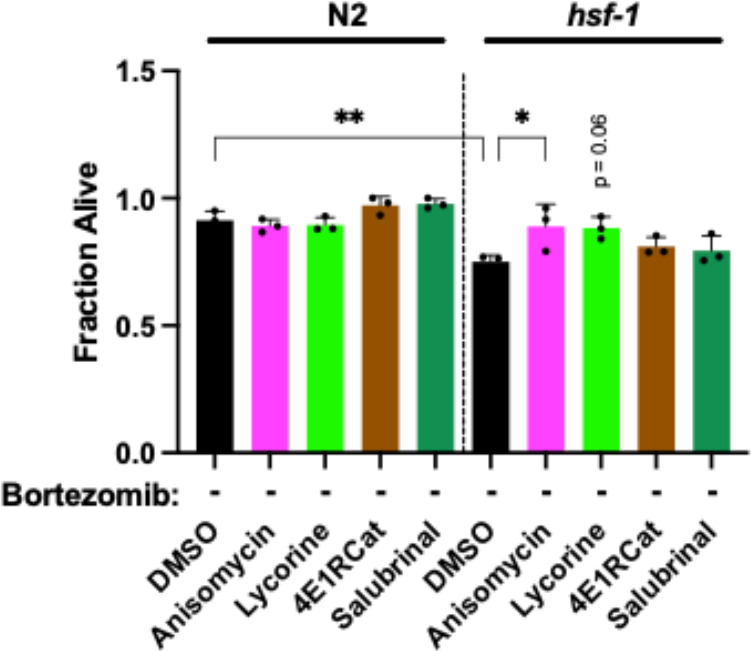
Survival of N2 and *hsf-1(sy441)* animals treated with translation inhibitors. Non-bortezomib-treated controls for Figure 4B. Treatment with inhibitor alone did not affect the lifespan of N2 worms when survival was measured at day 8. Interestingly, in *hsf-1(sy441)* animals, anisomycin alone significantly improves survival without bortezomib treatment, and similarly, lycorine trends toward significance (p=0.06). Data are displayed as mean ± SD and **=p < 0.01 and *=p < 0.05 by one-way ANOVA with Dunnet’s multiple comparisons test. Total of three independent experiments. Figure 4—figure supplement 1—source data 1.Quantification of survival of N2 and *hsf-1(sy441)* not treated with bortezomib.

We next asked if the induction of the *sma* phenotype was specific to *hsf-1(sy441)* animals or if the combination of proteasome toxicity with impaired protein folding capacity caused it. We, therefore, treated PolyQ35::YFP transgenic animals for 12 hr with the four different translation inhibitors, followed by bortezomib. As expected, treating PolyQ::YFP animals with bortezomib caused extensive protein aggregation. As before, the inhibition of TI exacerbated bortezomib toxicity and was not further quantified. As with *hsf-1(sy441),* bortezomib treatment also induced the *sma* phenotype in the PolyQ35::YFP transgenic animals, which was almost entirely rescued by anisomycin (Figure 4E and F).

We then directly tested the effect of each inhibitor on proteasome activity in both N2 and *hsf-1(sy441)* animals. TE inhibitors did not seem to affect proteasome activity in either strain. In contrast, TI inhibitors reduced N2 proteasome activity compared to DMSO-treated controls, providing a rationale for their increased toxicity in our proteasome survival assay (Figure 4G). *hsf-1(sy441)* mutants showed significantly decreased proteasome activity compared to N2 controls, which was not further reduced by TI inhibitors in *hsf-1(sy441)*.

Therefore, inhibition of TI exacerbates bortezomib-induced proteasome stress, while TE inhibition provides limited protection. However, TE inhibitors were highly protective in the context of reduced folding capacity. We concluded that there are distinct protective mechanisms that are employed depending on the way translation is inhibited and the type of stress challenging the animal.

### Lifespan extension by translational elongation and initiation inhibitors is dictated by genetic background

**Figure 5.**
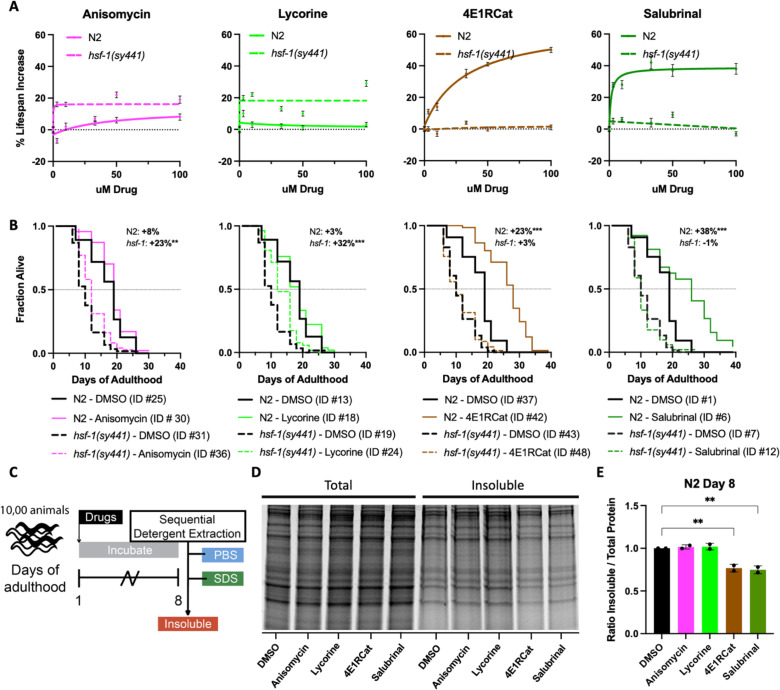
Reciprocal lifespan extension by translation inhibitors in N2 and *hsf-1(sy441)* animals. (**A**) Graphs show mean lifespan as a function of translation inhibitor concentration. TI inhibitors increase the lifespan of N2 but not *hsf-1(sy441)* animals, with a maximum effect at 100 μM. TE inhibitors increase the lifespan of *hsf-1(sy441)* but not N2 animals. Error bars indicate ± SEM. See Supplementary file 1 for the number of animals and repeats. (**B**) Survival curves from representative experiments show the fraction of wild-type (N2, solid line) or *hsf-1* mutant (dashed line) animals when treated with 100 μM of the indicated compound. Black lines indicate DMSO treatment, and colored lines indicate inhibitor treatment. Data are displayed as a Kaplan-Meier survival curve, and significance was determined by the log-rank test. ID # refers to the unique entry within Supplementary file 1. (**C**) Experimental strategy for treating animals and isolating detergent-insoluble fractions. 10,000 animals were treated and allowed to age for 8 days before being washed with M9, frozen in liquid nitrogen, and mechanically lysed. Then proteins were extracted from the total lysate based on solubility, and an aliquot from each fraction was run on an SDS-PAGE gel. (**D**) Representative SDS-PAGE gel stained with Sypro Ruby. 4E1RCat and salubrinal reduce insoluble protein at day 8. (**E**) Quantification of two separate experiments shows 4E1RCat and salubrinal significantly reduce insoluble protein in wild-type (**N2**) animals. Data are displayed as mean ± SEM and **=p < 0.01 by two-tailed Student’s t-test. Figure 5—source data 1.Summary of lifespan data used to construct dose-response graphs. Figure 5—source data 2.Lifespan data used to construct graphs. Figure 5—source data 3.Unedited gels. Figure 5—source data 4.Quantification of insoluble extractions.

Lowering translation is an established mechanism to extend lifespan and delay aging (Steffen and Dillin, 2016; Anisimova et al., 2018; Klaips et al., 2018; Hansen et al., 2007; Pan et al., 2007). Furthermore, aging is a well-known driver of protein aggregation. However, to our knowledge, it has never been investigated if the anti-aggregation and anti-aging effects of translational inhibition can be uncoupled and if the mode of translational inhibition influences these phenotypes. Inhibition of translation in wild-type animals using the two TE inhibitors, anisomycin and lycorine, showed no, or only a minor, lifespan extension in N2 animals (Figure 5A). In contrast, inhibition of TI by the inhibitors 4E1RCat and salubrinal dose-dependently extended lifespan of N2 animals (Figure 5B). This difference was observed despite all four translation inhibitors reducing protein translation to the same extent (Figure 1B). Thus, the difference in the effect on lifespan by TE and TI inhibitors cannot be explained by reducing overall protein synthesis alone.

**Figure 5—figure supplement 1.**
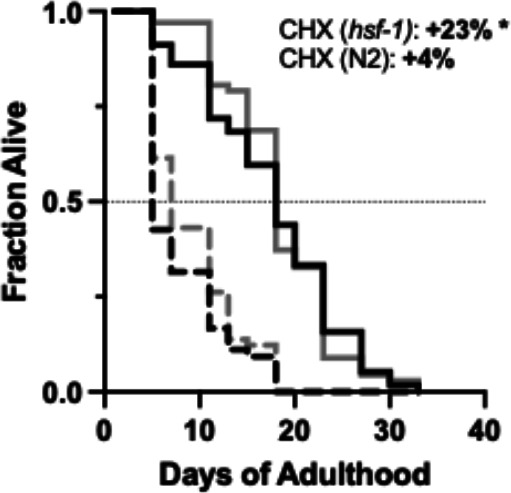
Cycloheximide, an elongation inhibitor, extends lifespan in *hsf-1(sy441)* but not N2. Cycloheximide phenocopies other translation elongation (TE) inhibitors where it does not extend lifespan in N2 at 100 μΜ while increasing lifespan in *hsf-1(sy441)* mutant animals. We note the p-value is less significant than when anisomycin or lycorine are used. See Supplementary File IDs: #95, #98, #103, #108. Figure 5—figure supplement 1—source data 1.Lifespan data used to construct graphs.

While investigating the effect of inhibiting different modes of translation in our proteasome survival assay, we noticed that anisomycin and lycorine appeared to improve the survival of *hsf-1(sy441)* animals (Figure 4—figure supplement 1). We, therefore, tested the ability of all four translation inhibitors to extend lifespan in *hsf-1(sy441)* mutants. As expected, both 4E1RCat and salubrinal failed to extend lifespan in *hsf-1(sy441)* mutants significantly. This result is consistent with genetic work by both the Rodgers and Tavernarakis labs, which showed inhibition of TI extends lifespan dependent on HSF-1 (through *ifg-1* and *ife-2*, respectively) (Howard et al., 2016; Rieckher et al., 2018).

As Figure 4—figure supplement 1 hinted, treatment with the TE inhibitors anisomycin and lycorine significantly extended the lifespan of *hsf-1(sy441*) mutants by ~20% (Figure 5B). Similar results were obtained for the TE inhibitor cycloheximide (Figure 5—figure supplement 1). We interpret this lifespan extension as a partial rescue of the protein folding defect in *hsf-1(sy441*) mutants, as the increase in lifespan did not reach the lifespan of wild-type animals. Taken together, however, our data demonstrate that longevity induced by translation inhibition, whether through targeting TE or TI, subsumes several different mechanisms that lead to longevity.

We next tested the ability of all four inhibitors to reduce age-associated protein aggregation. If inhibition of translation alone is sufficient to reduce protein aggregation independently of any downstream mechanisms, all four inhibitors should reduce age-associated protein aggregation. Conversely, if the reduction of age-associated protein aggregation is closely linked to longevity, then only the TI inhibitors should reduce protein aggregation. We treated day 1, N2 animals with 100 μΜ of each of the four inhibitors and allowed the animals to age for 8 days, after which we separated proteins based on solubility (Figure 5C). We found that only the two TI inhibitors that extended lifespan caused significant decreases in the amount of SDS-insoluble aggregates and that the TE inhibitors failed to do so (Figure 5D and E).

Taken together, our data suggest that inhibition of TE rescues proteostasis-compromised animals by the *reduced folding load model* without generating additional protein folding capacity. Separately, inhibition of TI protects and improves longevity by the *selective translation model* that depends on HSF-1.

### Inhibition of translation initiation increases protein concentration in *hsf-1(sy441)* mutants

The canonical model that explains the requirement of HSF-1 to induce longevity by inhibiting TI suggests that blocking TI activates the HSR via HSF-1 that then initiates the transcription of HSP chaperones to increase folding capacity (Rogers et al., 2011; Howard et al., 2016). In contrast, work in cancer cells showed inhibition of translation to block HSF-1 binding to DNA and to specifically reduce the expression of HSP70 and DNAJA chaperones (Santagata et al., 2013), making it difficult to envision how HSF-1 could act as a downstream effector of TI.

**Figure 6.**
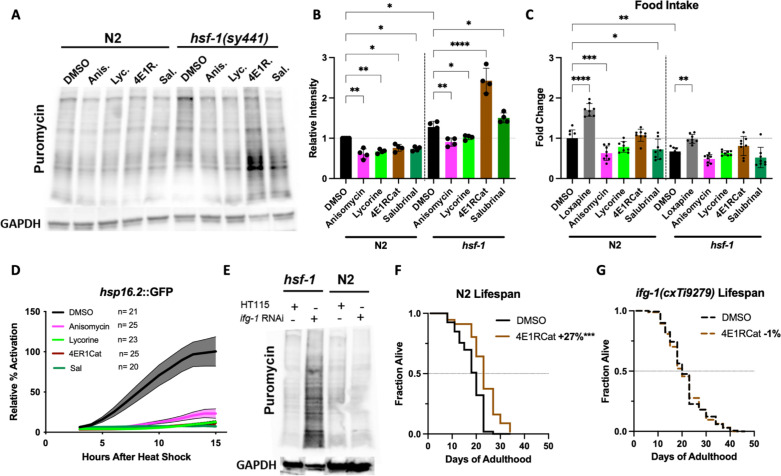
Inhibition of eIF4G/IFG-1 requires HSF-1 to lower the concentration of newly synthesized proteins. (**A**) *C. elegans* were treated with solvent (DMSO) or the indicated inhibitors (100 μM) for 12 hr, followed by a 4 hr puromycin incorporation in both N2 and *hsf-1(sy441)* background, which was immunoblotted against using an anti-puromycin antibody. *hsf-1(sy441)* mutant animals exhibited increased puromycin incorporation compared to N2 animals, which was further increased by both TI inhibitors 4E1RCat and salubrinal. (**B**) Quantification of four independent SUrface SEnsing of Translation (SUnSET) experiments in (A). Significance was determined by one-way ANOVA with Šídák multiple comparisons test where *=p ≤ 0.05, **=p ≤ 0.01, and **** p≤0.0001. Error bars indicate mean ± SD from four independent trials. (**C**) Food intake was quantified relative to DMSO-treated N2 controls measuring bacterial clearance from day 1 to day 4 of adulthood. Anisomycin and salubrinal significantly decrease food intake in wild-type N2. In *hsf-1(sy441)*, no inhibitor statistically changes food intake. Loxapine was used as a positive control in both genotypes. Significance was determined by one-way ANOVA with Šídák multiple comparisons test where *=p ≤ 0.05, **=p ≤ 0.01, and ****p≤0.0001. Error bars indicate mean ± SD from four independent trials. Total of eight independent experiments. (**D**) Translation inhibitors suppressed the heat shock response (HSR) as measured by *hsp-16.2*::GFP fluorescence assay. After incubation with the inhibitors for 4 days, followed by a 1 hr heat shock (HS) at 36°C, little to no increase in GFP expression was observed for each inhibitor, indicating that all inhibitors block HSR activation at the tested concentration (100 μM). Lines indicate mean, and shading indicates 95% CI. Representative of three independent experiments. (**E**) N2 and *hsf-1(sy441) C. elegans* were fed HT115 empty vector or RNAi against *ifg-1. ifg-1* depletion increased puromycin incorporation in *hsf-1* mutants but decreased incorporation in N2 animals, similar to 4E1RCat treatment. GAPDH was used as a loading control. Immunoblot is representative of three independent experiments. (**F**) 4E1RCat significantly increases lifespan in N2 animals. Data are displayed as a Kaplan-Meier survival curve, and significance is determined by the log-rank test. See ID #111 and #112 for details in Supplementary file 1. (**G**) 4E1RCat does not increase lifespan in *ifg-1(cxTi9279)* animals. See ID #93 and #94 for details in Supplementary file 1. Data are displayed as a Kaplan-Meier survival curve, and significance is determined by the log-rank test. Figure 6—source data 1.Unedited western blots. Figure 6—source data 2.Quantification of western blots. Figure 6—source data 3.Quantification of food intake. Figure 6—source data 4.Quantification of *hsp-16.2::GFP* reporter activation. Figure 6—source data 5.Unedited western blots with Coomassie staining of the membrane. Figure 6—source data 6.Lifespan data used to construct graphs. Figure 6—source data 7.Lifespan data used to construct graphs.

We, therefore, tested if HSF-1 could act upstream of TI either by controlling the expression of transcripts that become translated upon inhibition of translation or that HSF-1 (or one of its targets) directly contributes to the proper function of the TI machinery. We treated N2 or *hsf-1(sy441)* animals with the four inhibitors and measured the resulting concentration of newly synthesized proteins. As before, all four inhibitors reduced the concentration of newly synthesized proteins in N2. In contrast, TI inhibitors increased the concentration of newly synthesized proteins by 1.5- to 2-fold in the *hsf-1(sy441)* animals (Figure 6A and B). We confirmed that this effect is not due to increased intake of puromycin, as inhibitor treatment did not affect food intake (Figure 6C). Furthermore, none of the translation inhibitors induced the HSF-1-activated heat shock reporter *hsp-16.2::GFP* (Figure 6D).

To ensure that these results are indeed caused by the inhibition of TI and not an off-target effect, we knocked down the target of 4E1RCat, eIF4G/*ifg-1,* by RNAi in N2 and *hsf-1(sy441)* animals and measured the concentration of newly synthesized proteins. As seen for 4E1RCat, the knockdown of eIF4G/*ifg-1* reduced the protein concentration in N2 but dramatically increased it in *hsf-1(sy441)* mutants (Figure 6E). Unfortunately, we could not determine the exact target for salubrinal in *C. elegans*.

To further confirm that the lifespan extension of 4E1RCat is caused by on-target action, we measured lifespan extension in N2 and *ifg-1(cxTi9279)* mutants which carry a splicing defect that results in an overall reduction of eIF4G/*ifg-1* protein (Morrison et al., 2014). As we previously observed (Figure 5A and B) 4E1RCat treatment of N2 animals significantly extended lifespan (Figure 6F). However, no lifespan extension was observed in *ifg-1(cxTi9279)* animals (Figure 6G), showing that 4E1RCat extends lifespan by on-target action. Therefore, we concluded that inhibition of eIF4G/*ifg-1* requires HSF-1 to lower the concentration of newly synthesized proteins. Consequently, the beneficial effects of 4E1RCat treatment are not observed in *hsf-1(sy441)* mutants as inhibition of eIF4G/*ifg-1* fails to lower protein concentrations in this strain.

## Discussion

A substantial body of genetic and biochemical work suggests that lowering the concentration of newly synthesized proteins reduces the load on the proteostasis machinery. We asked if these effects could be pharmacologically replicated to exploit them for therapeutic purposes in disease models (Solis et al., 2018; Schubert et al., 2018).

We set out to study how pharmacological inhibition of TI or TE improves proteostasis and increases longevity in adult *C. elegans*. We intended to compare the *selective translation* model, in which the selective reduction of specific proteins and the selective translation of others protect from proteotoxicity to the *reduced folding load model*, in which the non-specific reduction of the concentration of newly synthesized proteins is protective.

We compared the non-selective lowering of protein concentration by TE inhibitors to the selective lowering of initiation-dependent proteins by TI inhibitors. Our studies reveal that pre-existing proteotoxicity, as might be observed in a disease state, dictates the mode of translation inhibition necessary to restore proteostasis. As a result, the mode of translation inhibition, either inhibiting TI or TE, results in distinct and sometimes opposing outcomes for the health of the animals.

The most striking dichotomy was seen when pre-existing proteotoxicity—caused by the lack of either HSF-1 or the expression of the aggregation-prone PolyQ protein—was combined with the inhibition of the proteasome. These combined insults led to increased protein aggregation, shrinking body size, and death of the animals (Figure 4). Inhibition of TE almost entirely rescued these phenotypes. In sharp contrast, inhibition of TI exacerbated proteotoxicity and increased mortality. The effects were consistent for both TE and TI inhibitors despite structural differences and different targets of the TI inhibitors.

The second example of this dichotomy was the reciprocal ability of TI and TE inhibitors to extend the lifespan of N2 wild-type animals or *hsf-1(sy441)* mutant animals, with each mode of inhibition only able to extend the lifespan in one of the strains. Overall, TE inhibition effectively rescued proteostasis in animals with pre-existing proteotoxicities, such as a compromised HSR, reduced proteasome activity, or the expression of aggregation-prone proteins. Conversely, inhibition of TI did little to rescue pre-existing proteotoxicity or worsen it but proved effective at preventing emerging proteotoxicity, for example, with age. Notably, both anisomycin and cycloheximide have previously been reported to extend lifespan in wild type animals (Tarkhov et al., 2019; Takauji et al., 2016) but these studies were conducted in either non-fully reproductive adults or the exact conditions were not specified (i.e. temperature, whether OP50 feeding bacteria was alive/dead and developmental stage; Takauji et al., 2016). In contrast, we focused on the effects of inhibitor treatment on the lifespan of fully reproductive adult animals—thereby eliminating any partial dependency on developmental effects. Overall, our findings establish that inhibition of TI or TE has distinct consequences for proteostasis and that their mechanisms to control the concentration of newly synthesized proteins can be genetically separated.

We considered the following reasons to explain the different consequences for proteostasis. The increased mortality observed after combining bortezomib with TI inhibitors is likely to be the result of the lower expression of HSP90/*daf-21* and HSP70/*hsp-1*, whose translation depends on eIF4G/*ifg-1* (Rogers et al., 2011) and the ability of both treatments to lower proteasome activity independently (Figure 4). The lower proteasome activity induced by the TI inhibitors is likely to enhance the proteotoxicity of bortezomib. The lowered proteasome activity observed upon inhibition of TI was surprising as the short-term inhibition of translation increases proteasome activity (Zhao et al., 2015). However, compared to most other studies, we chose to reduce translation by only ~40%, as such a reduction is more likely to be tolerated chronically if translation inhibitors could be developed into therapeutics. In contrast, TE inhibitor treatment alone did not lower proteasome activity. More importantly, lowering the concentration of newly synthesized proteins by inhibiting TE rescued most bortezomib-induced proteotoxic effects. Thus, long-term inhibition of TI or TE differentially affects proteasomal activity and its proteotoxic effects.

The most unequivocal difference was the dependency of TI inhibitors on HSF-1, which was not observed for TE inhibitors. This dependency of the TI inhibitors on HSF-1 was initially explained by a model in which inhibition of TI activates the HSR via HSF-1. This model has been previously proposed for eIF4G/*ifg-1* (Rogers et al., 2011; Howard et al., 2016).

This initial model is still likely valid, but our study shows that HSF-1 has a more immediate role. HSF-1, or one of its targets, is necessary for the TI machinery to control protein synthesis. *Selective translation* can only occur if TI itself is inhibited. As seen in Figure 6, inhibition of TI in the absence of HSF-1 increases, rather than decreases, the amount of newly synthesized proteins. This surprising increase in newly synthesized proteins upon 4ER1Cat treatment was not an off-target effect as it was replicated by RNAi-mediated knockdown of eIF4G/*ifg-1* in an HSF-1 mutant background. Hence, HSF-1 activity directly or indirectly modulates the ability of TI components to control the concentration of newly synthesized proteins.

At the outset, we intended to compare the *selective translation* model to the *reduced folding load model* by comparing their effects on proteostasis under conditions in which inhibition of translation resulted in the same concentration of newly synthesized proteins, irrespective of the mode of translation inhibition. We observed apparent differences in proteostasis effects, supporting the *reduced folding load model* for inhibiting TE and the *selective translation model* for inhibiting TI.

The inability of TI inhibitors to reduce the concentration of newly synthesized proteins in *hsf-1(sy441)* mutants and the inability to extend their lifespan shows that lowering the concentration of newly synthesized proteins is necessary for the beneficial effects. On the other hand, the finding that TE inhibitors protect from proteotoxic stress but do not extend lifespan shows that lowering the concentration of newly synthesized proteins is sufficient to protect from proteotoxic stress but is not sufficient to extend lifespan in wild-type, which appears to require *selective translation*.

### Ideas and speculation

Our data show that HSF-1 or one of its targets controls the ability of TI to change the concentration of newly synthesized proteins. Previous work noted a surprising requirement for HSF-1 in the protection from ER stress elicited by the RNAi-mediated knockdown of eIF4G/*ifg-1* (Howard et al., 2016). The authors note that further investigations will be required to determine the direct link between enhanced HSR amelioration of proteotoxicity and the endoplasmic reticulum. Our results provide a compelling explanation for their findings, in that the lack of HSF-1 abolishes the ability of *ifg-1* RNAi to lower the concentration of newly synthesized proteins and thus the protection from tunicamycin toxicity.

In the context of TI inhibition, HSF-1 (or one of its targets) may influence the concentration of newly synthesized proteins by either modulating protein degradation or synthesis. Our data show that proteasome activity and, by extension, degradation are lowered by both inhibition of TI and lack of HSF-1. Hence, combining both may indirectly increase the concentration of newly synthesized proteins through reduced degradation. However, inhibition of TI did not further decrease the already low proteasome activity in HSF-1 mutants (Figure 4). Furthermore, salubrinal and 4ER1Cat lowered proteasome activity by the same amount, yet 4ER1Cat treatment increased the concentration of newly synthesized proteins much more in the HSF-1 background (Figure 6B and E). Thus, we concluded a model in which HSF-1 promotes degradation in response to the inhibition of the TI machinery less likely.

Alternatively, HSF-1 (or one of its targets) cooperates with TI factors to form a checkpoint that prevents random unregulated translation. The TI factors are part of the checkpoint but also initiate regulated translation by giving ribosomes access to mRNA. Thus, when TI factors are inhibited or knocked down, HSF-1 or one of its targets maintains the checkpoint preventing uncontrolled translation. Without any TI activity, maintaining the checkpoint reduces protein synthesis. In the *hsf-1(sy441)* mutant, the TI machinery still prevents uncontrolled translation but less effectively, leading to a slight but detectable increase in newly synthesized proteins in HSF-1 mutants (Figure 6A). Once the TI and HSF-1 are removed, ribosomes gain access to many mRNAs leading to unregulated translation and a dramatic increase in newly synthesized proteins.

Both the degradation and the cooperative checkpoint model explain the observed results but need to invoke a speculative connection between HSF-1 to either TI or protein degradation. Our results unexpectantly couple the ability of the TI machinery to control the concentration of newly synthesized proteins to HSF-1, thereby explaining its requirement. However, our results do not provide a mechanism by which HSF-1 couples the concentration of newly synthesized proteins to TI.

## Materials and methods

**Key resources table:** 

Reagent type (species) or resource	Designation	Source or reference	Identifiers	Additional information
Strain, strain background (*Caenorhabditis elegans*)	N2	*Caenorhabditis* Genetics Center (CGC)	RRID:WB-STRAIN:WBStrain00000003	Wild-type (Bristol)
Strain, strain background (*Caenorhabditis elegans*)	CL2070	CGC	RRID:WB-STRAIN:WBStrain00005096	*dvIs70 [hsp-16.2p::GFP+rol-6(su1006)]*
Strain, strain background (*Caenorhabditis elegans*)	AM140	CGC	RRID:WB-STRAIN:WBStrain00000182	*rmIs132Punc-54::q35::yfp*
Strain, strain background (*Caenorhabditis elegans*)	KX54	CGC	RRID:WB-STRAIN:WBStrain00024080	*ifg-1(cxTi9279*)
Strain, strain background (*Caenorhabditis elegans*)	PS3551	CGC	RRID:WB-STRAIN:WBStrain00007673	*hsf-1(sy441*)
Genetic reagent (*Caenorhabditis elegans*)	*myo3p::*GFP-IRES*-*tdTomato	This paper		Adapted from: DOI: 10.2144/000113821 See Materials and methods, Method for making bi-cistronic vector
Antibody	Anti-puromycin (Mouse monoclonal)	MilliporeSigma	Cat#: MABE343 RRID:AB_2566826	1:5000
Antibody	Anti-GAPDH (Rabbit polyclonal)	Proteintech	Cat#: 1094-1-AP RRID:AB_2263076	1:5000
Antibody	Anti-myc (Mouse Monoclonal)	Cell Signaling	Cat#: 2276S RRID:AB_331783	1:2000
Antibody	Anti-mouse—HRP (secondary)	Cell Signaling	Cat#: 7076S, RRID:AB_330924	1:5000
Antibody	Anti-rabbit—HRP (secondary)	Cell Signaling	Cat#: 7074S, RRID:AB_2099233	1:5000
Commercial Assay or kit	20S Proteasome Activity Assay Kit	Sigma-Aldrich	Cat#: APT280	
Commercial Assay or kit	SYPRO Ruby Protein Gel Stain 1×	Bio-Rad	Cat#: 1703125	
Chemical compound, drug	Anisomycin	MedChemExpress	Cat#: HY-18982	
Chemical compound, drug	Lycorine hydrochloride	Combi-Blocks	Cat#: QW-2476	
Chemical compound, drug	4E1RCat	MedChemExpress	Cat#: HY-14427	
Chemical compound, drug	Puromycin	Sigma-Aldrich	Cat#: P8833	
Chemical compound, drug	Salubrinal	MedChemExpress	Cat#: HY-15486	
Chemical compound, drug	Bortezomib, free base	LC Laboratories	Cat#: B-1408	

### Lead contact and materials availability

Michael Petrascheck is the Lead Contact and may be contacted at pscheck@scripps.edu. This study did not generate new unique reagents; however, the natural product Amicoumacin C was obtained as a gift from Dr Shigefumi Kuwahara, Ph.D. (Tohoku University). This reagent is not available without total chemical synthesis.

### *C. elegans* strains

The Bristol strain (N2) was used as the wild-type strain. In addition, the following worm strains used in this study were obtained from the Caenorhabditis Genetics Center (CGC; Minneapolis, MN, USA): CL2070 [dvIs70 [*hsp*-16.2p::GFP+*rol-6(su1006)*]], AM140 [rmIs132[P*unc-54*::q35::yfp]], KX54 [*ifg-1(cxTi9279*)] and PS3551 [*hsf-1(sy441*)].

### Method details

#### Worm maintenance

1000–2000 age-synchronized animals were plated into 6 cm culture plates with liquid medium (S-complete medium with 50 mg/mL carbenicillin and 0.1 mg/mL fungizone [amphotericin B]) containing 6 mg/mL X-ray irradiated *Escherichia coli* OP50 (1.5×10^8^ colony-forming units [cfu]/mL, carbenicillin resistant to exclude growth of other bacteria), freshly prepared 4 days in advance, as previously described (Solis and Petrascheck, 2011), and were maintained at 20°C. The final volume in each plate was 7 mL. To prevent self-fertilization, FUDR (5-fluoro-2’-deoxyuridine, 0.12 mM final) (Sigma-Aldrich, Cat#: 856657) was added 42—45 hr after seeding. At the late L4 stage, either DMSO/drug treatment (100 μΜ unless otherwise stated) was added to each strain.

#### RNAi

RNAi of *ifg-1* was preformed using the feeding vector L4440 to express dsRNA of the respective genes, received as a gift from the Hansen lab. HT115 bacteria were as OP50 bacteria above, with the exception that prior to harvesting, 2 mM IPTG (isopropyl β-D-1-thiogalactopyranoside) was added, and the suspension was allowed to grow for 4 additional hours to induce dsRNA expression. Animals were grown and synchronized in liquid culture as previously described, being fed the relevant RNAi bacteria.

#### SUnSET to analyze the effectiveness of translation inhibitors in *C. elegans*

Day 1 adult N2 worms were bleached, and eggs were allowed to hatch in S-complete by shaking overnight. On the next day, 12,000 L1 worms were seeded in a 15 cm plate containing a total volume of 30 mL S-complete with 6 mg/mL OP50 bacteria, 50 μg/mL carbenicillin, and 0.1 μg/mL amphotericin B. Six mL of 0.6 mM FUDR were added to worms at L4 stage in each plate. 100 μΜ translation inhibitor was added to worms 2 hr after adding FUDR. After 12 hr, worms were transferred into a 15 mL corning tube containing a total volume of 5 mL S-complete with 750 µL 6 mg/mL OP50 bacteria, 0.5 mg/mL puromycin, and 100 μΜ translation inhibitors. After rotating the corning tubes for 4 hr, worms were collected into 2 mL cryotubes by washing them with M9 once and with cold PBS three times. Worms were flash-frozen in liquid nitrogen and subsequently broken with a beak mill homogenizer (Fisherbrand). Protein concentrations were determined by the Bradford protein assay. 50 mg protein from each sample was loaded for western blot analysis using antibodies against puromycin (Millipore, MABE343) and GAPDH (Proteintech, 10494-1-AP). Antibodies were diluted 1:5000 in 5% non-fat milk in TBST.

#### Method for making bi-cistronic vector

The body wall muscle-specific promotor of the bi-cistronic vector is from 2 kb upstream of the start codon of gene myo-3. GFP coding sequence is after the promotor. The IRES element is from 285 bp upstream of the hsp-3 start codon (Li and Wang, 2012). The Tdtomato coding sequence is after the IRES fragment. A 3Xmyc fragment is inserted in front of the tdtomato sequence. All fragments were ligated together by Gibson Assembly method (New England Biolabs).

#### Determination of GFP and tdTomato fluorescence

Day 1 adult bi-cistronic reporter worms were bleached, and eggs were allowed to hatch in S-complete by shaking overnight. On the next day, 12,000 L1 worms were seeded in a 15 cm plate containing a total volume of 30 mL S-complete with 6 mg/mL OP50 bacteria, 50 μg/mL carbenicillin, and 0.1 μg/mL amphotericin B. Six mL of 0.6mM FUDR was added, and two hours later, the animals were treated with the indicated translation inhibitor at a final concentration of 100 μM or DMSO only. At the L4 stage, 500 GFP-positive animals per condition were sorted using the COPAS Biosorter (Union Biometric). The integral values for fluorescence on a corresponding channel (i.e. green or red) were used to determine simultaneous red and green measurements for each animal using the standard BioSorter software. Integral values are determined automatically by the BioSorter by integrating each signal when the threshold signal is above the threshold value. In essence, it determines intensity over the length of the worm to account for variations in size.

#### Thermotolerance

Age-synchronized N2 or PS3551 [*hsf-1(sy441*)] animals were prepared as above in 6 cm culture plates and treated with water or 100 μM lycorine/anisomycin on day 1. On day 4, 25–35 animals were transferred to 6 cm NGM plates in triplicate for each condition and were transferred to the non-permissive temperature of 36°C. Every hour, survival was scored by lightly touching animals with a worm pick and scoring for movement.

#### *C. elegans* insoluble protein extraction

10,000 N2 worms were sorted into a 15 cm liquid culture dish using the COPAS Biosorter (Union Biometrica). For heat shock-induced aggregation experiments, worms were treated with either DMSO or anisomycin (100 μΜ) for 12 hr on day 1 of adulthood, then subjected to a 2 hr heat shock at 36°C. After 12 hr of recovery, the animals were washed three times with S-complete buffer, once with PBS, and then flash-frozen in liquid nitrogen. 500 µL of cold lysis buffer (20 mM Tris base, 100 mM NaCl, 1 mM MgCl_2_, pH = 7.4, with protease inhibitors [Roche, 11836153001]) was added, and animals homogenized mechanically. An aliquot of this total lysate was saved. In an ultracentrifuge tube, two volumes of SDS Extraction buffer (20 mM Tris base, 100 mM NaCl, 1 mM MgCl_2_, pH = 7.4, with protease inhibitors, and 1% SDS) were added to 1 volume of total lysate and was centrifuged at 20,000 × *g* for 30 min. The extraction was repeated two times to remove all SDS-soluble proteins. The remaining insoluble pellet was suspended briefly in 20 µL urea buffer (8 M urea, 50 mM DTT, 2% SDS, 20 mM Tris base, pH = 7.4) and sonicated. 18 µL of the insoluble suspension was added to 6 µL 4× Laemmli buffer (Bio-Rad, #161-0747) supplemented with 10% 2-mercaptoethanol (Sigma, 60-24-2) and boiled for 5 min, then directly loaded onto SDS-PAGE gel (Bio-Rad, 4569033). Gels were stained with Sypro Ruby according to the manufacturer’s directions. Gels were quantified in ImageJ by dividing the integrated intensity of each full insoluble lane by the integrated intensity of the corresponding full total protein lane after subtracting a similar area background lane, then normalizing to the DMSO control.

For age-associated protein aggregation experiments, the above was repeated with the following changes: Day 1 worms were treated with 100 μΜ of each compound and allowed to age in liquid culture until day 8 of adulthood. Following lysis, worms were washed several times with PBS to remove soluble protein.

#### Proteasome dysfunction assay—survival

Animals were prepared as above in 96-well plates. At the late L4 stage, animals were pre-treated with DMSO or 100 μΜ anisomycin. After 12 hr, the animals were treated with 75 μΜ bortezomib. On day 8 of adulthood, the percentage of animals alive was determined by movement in liquid culture.

#### Proteasome dysfunction assay—worm length

Animals were prepared as above in 6 cm liquid culture dishes. At the late L4 stage, animals were pre-treated with DMSO or 100 μΜ anisomycin. After 12 hr, the animals were treated with 75 μΜ bortezomib. Body length was measured on day 3 for *hsf-1(sy441)* or day 5 for PolyQ using the 10× objective with the ImageXpress Micro XL and Metaexpress microscopy software.

#### 20S proteasome activity assay

The Chemicon 20S Proteasome Activity Assay Kit (Cat. No APT280) was used according to the manufacturer’s instructions. In short, 10,000 age-synchronized animals were grown in liquid culture on 10 cm plates. DMSO or inhibitors were added on day 1. On day 5, animals were collected and washed 3× with cold DPBS, then once with 1× assay buffer. Worms were flash-frozen in liquid nitrogen. After three biological replicates were harvested in this way, the frozen animals were broken open with a beak mill homogenizer (Fisherbrand). Protein concentrations were determined by the Bradford protein assay. 200 μg of sample was loaded with assay mixture into a 96-well plate and incubated for 1 hr at 37°C. Fluorescence was measured on the Tecan Safire II with a 380/460 nm filter set. Positive control: 5 µL of 20S proteasome positive control (Chemicon Part No. 90205). Negative control: N2 lysate treated with 25 mM lactacystin, a 20S proteasome inhibitor (Chemicon Part No. 90208).

#### Heat shock-induced aggregation and stress response

CL2070 [dvIs70 [*hsp-16.2p*::GFP+*rol-6*(su1006)]] or AM140 [rmIs132[Punc-54::q35::yfp]] age-synchronized animals were treated with DMSO or 100 μΜ drug at late L4. 4–12 hr later, on day 1 of adulthood, 1.5 mL of the treated animals were transferred from liquid culture into an Eppendorf tube, washed twice with S-complete, pelleted, and then transferred to 6 cm NGM plates using S-complete. Once the animals were completely dry on the NGM plate, they were transferred to a 36°C incubator, plates upside down, for 1–2 hr.

#### Hydrogel mounting

Animals were washed from NGM plates using 0.2% HHPPA (2-hydroxy-4’-(2-hydroxyethoxy)-2-methylpropiophenone) (CAS[106797-53-9]) dissolved in S-complete into a 2 mL Eppendorf tube. Animals were washed twice with 0.2% HHPPA, then suspended in 0.3 mL 0.2% HHPPA. 2.5 µL of this solution was seeded into a single well of a 384-well plate containing 2.5 µL 30% PEG-DA (polyethylene glycol diacrylate, MW = 4000, Polysciences, Cat#: 15246-1) in S-complete. After 5 min, to allow the solutions to diffuse, the animals were immobilized by subjecting the 384-well plate UV light using a routine laboratory gel viewer (UVP Dual-Intensity Ultraviolet Transilluminator, high intensity) for 30 s. 45 µL S-complete buffer was added on top to prevent desiccation. In general, each well contained 5–10 worms.

#### Imaging and analysis

Time-lapse brightfield and fluorescence images were taken with a 10× objective using the ImageXpress Micro XL over 15 hr. The number of PolyQ aggregates, or total YFP fluorescence, in the whole worm, was determined by analyzing images using a custom pipeline created in CellProfiler.

#### Lifespan assay

Age-synchronized *C. elegans* were prepared in a liquid medium, as described above, and seeded into flat-bottom, optically clear 96-well plates (Corning, 351172) containing 150 µL total volume per well, as previously described (Clay and Petrascheck, 2020). Plates contained ~10 animals per well in 6 mg/mL γ-irradiated OP50. Age-synchronized animals were seeded as L1 larvae and grown at 20°C. Plates were covered with sealers to prevent evaporation. To prevent self-fertilization, FUDR (0.12 mM final) was added 42—45 hr after seeding. Drugs were added on day 1 of adulthood. DMSO was kept to a final concentration of 0.33% vol/vol when used.

### Quantification and statistical analysis

#### Aggregation and induction of the HSR

The number of n represents the total number of animals over three individual experiments. For the paired-time-lapse data generated, we chose to depict the 95% confidence interval, calculated by GraphPad Prism, to show differences in treatment.

#### Quantification of western blot

To determine the relative intensities of each blot, the integrated intensity (pixel intensity divided by pixel area) was measured for each full lane using ImageJ. A similar-sized band with no signal was used to subtract the background, and then each intensity normalized to its corresponding GAPDH loading control. Finally, each band’s integrated intensity was normalized to the wild-type DMSO control for quantification and statistics. Significance was determined by one-way ANOVA with Šídák multiple comparisons test.

#### Percent survival—thermotolerance assay

The number of n represents the total number of animals over the three individual replicates shown as the average percentage survival and SEM, calculated using GraphPad Prism. Significance was determined by using a row-matched two-way ANOVA with Šídák multiple comparisons test.

#### Worm length—proteasome dysfunction assay

The number of n represents the total number of animals whose length was measured in one experiment. Depicted are the mean and standard deviation calculated using GraphPad Prism. Significance was determined by the two-tailed unpaired t-test. Similar results were observed across three independent experiments.

#### Lifespan assay

Survival was scored manually by visually monitoring worm movement using an inverted microscope thrice weekly. Statistical analysis was performed using the Mantel-Haenzel version of the log-rank test as outlined in Petrascheck and Miller, 2017.

### Code availability

The software used in this study (Cell Profiler) is available at https://cellprofiler.org/.

## Data Availability

All data generated or analysed during this study are included in the manuscript and supporting file.

## Additional files

Supplementary file 1. Summary of lifespan studies. All experiments were conducted with dead, γ-irradiated bacteria (OP50) and conducted in a 96-well liquid culture.

Transparent reporting form

## References

[bib1] Anisimova AS, Alexandrov AI, Makarova NE, Gladyshev VN, Dmitriev SE (2018). Protein synthesis and quality control in aging. Aging.

[bib2] Arnold A, Rahman MM, Lee MC, Muehlhaeusser S, Katic I, Gaidatzis D, Hess D, Scheckel C, Wright JE, Stetak A, Boag PR, Ciosk R (2014). Functional characterization of *C. elegans* Y-box-binding proteins reveals tissue-specific functions and a critical role in the formation of polysomes. Nucleic Acids Research.

[bib3] Balch WE, Morimoto RI, Dillin A, Kelly JW (2008). Adapting proteostasis for disease intervention. Science.

[bib4] Brignull HR, Morley JF, Garcia SM, Morimoto RI (2006). Modeling polyglutamine pathogenesis in *C. elegans*. Methods in Enzymology.

[bib5] Choe Y-J, Park S-H, Hassemer T, Körner R, Vincenz-Donnelly L, Hayer-Hartl M, Hartl FU (2016). Failure of RQC machinery causes protein aggregation and proteotoxic stress. Nature.

[bib6] Chu J, Hong NA, Masuda CA, Jenkins BV, Nelms KA, Goodnow CC, Glynne RJ, Wu H, Masliah E, Joazeiro CAP, Kay SA (2009). A mouse forward genetics screen identifies LISTERIN as an E3 ubiquitin ligase involved in neurodegeneration. PNAS.

[bib7] Clay KJ, Petrascheck M, Curran SP (2020). Aging: Methods and Protocols.

[bib8] David DC, Ollikainen N, Trinidad JC, Cary MP, Burlingame AL, Kenyon C (2010). Widespread protein aggregation as an inherent part of aging in *C. elegans*. PLOS Biology.

[bib9] Dmitriev SE, Vladimirov DO, Lashkevich KA (2020). A quick guide to small-molecule inhibitors of eukaryotic protein synthesis. Biochemistry. Biokhimiia.

[bib10] Hansen M, Taubert S, Crawford D, Libina N, Lee SJ, Kenyon C (2007). Lifespan extension by conditions that inhibit translation in *Caenorhabditis elegans*. Aging Cell.

[bib11] Howard AC, Rollins J, Snow S, Castor S, Rogers AN (2016). Reducing translation through eIF4G/IFG-1 improves survival under ER stress that depends on heat shock factor HSF-1 in *Caenorhabditis elegans*. Aging Cell.

[bib12] Klaips CL, Jayaraj GG, Hartl FU (2018). Pathways of cellular proteostasis in aging and disease. The Journal of Cell Biology.

[bib13] Lan J, Rollins JA, Zang X, Wu D, Zou L, Wang Z, Ye C, Wu Z, Kapahi P, Rogers AN, Chen D (2019). Translational regulation of non-autonomous mitochondrial stress response promotes longevity. Cell Reports.

[bib14] Li D, Wang M (2012). Construction of a bicistronic vector for the co-expression of two genes in *Caenorhabditis elegans* using a newly identified IRES. BioTechniques.

[bib15] McQuary PR, Liao C-Y, Chang JT, Kumsta C, She X, Davis A, Chu C-C, Gelino S, Gomez-Amaro RL, Petrascheck M, Brill LM, Ladiges WC, Kennedy BK, Hansen M (2016). *C. elegans* S6K Mutants require a Creatine-Kinase-like effector for lifespan extension. Cell Reports.

[bib16] Medicherla B, Goldberg AL (2008). Heat shock and oxygen radicals stimulate ubiquitin-dependent degradation mainly of newly synthesized proteins. The Journal of Cell Biology.

[bib17] Morley JF, Brignull HR, Weyers JJ, Morimoto RI (2002). The threshold for polyglutamine-expansion protein aggregation and cellular toxicity is dynamic and influenced by aging in *Caenorhabditis elegans*. PNAS.

[bib18] Moronetti Mazzeo LE, Dersh D, Boccitto M, Kalb RG, Lamitina T (2012). Stress and aging induce distinct polyQ protein aggregation states. PNAS.

[bib19] Morrison JK, Friday AJ, Henderson MA, Hao E, Keiper BD (2014). Induction of cap-independent BiP (hsp-3) and Bcl-2 (ced-9) translation in response to eIF4G (IFG-1) depletion in *C. elegans*. Translation.

[bib20] Nedialkova DD, Leidel SA (2015). Optimization of codon translation rates via tRNA modifications maintains proteome integrity. Cell.

[bib21] Nollen EAA, Garcia SM, van Haaften G, Kim S, Chavez A, Morimoto RI, Plasterk RHA (2004). Genome-wide RNA interference screen identifies previously undescribed regulators of polyglutamine aggregation. PNAS.

[bib22] Pan KZ, Palter JE, Rogers AN, Olsen A, Chen D, Lithgow GJ, Kapahi P (2007). Inhibition of mRNA translation extends lifespan in *Caenorhabditis elegans*. Aging Cell.

[bib23] Petrascheck M, Miller DL (2017). Computational analysis of lifespan experiment reproducibility. Frontiers in Genetics.

[bib24] Reis-Rodrigues P, Czerwieniec G, Peters TW, Evani US, Alavez S, Gaman EA, Vantipalli M, Mooney SD, Gibson BW, Lithgow GJ, Hughes RE (2012). Proteomic analysis of age-dependent changes in protein solubility identifies genes that modulate lifespan. Aging Cell.

[bib25] Riback JA, Katanski CD, Kear-Scott JL, Pilipenko EV, Rojek AE, Sosnick TR, Drummond DA (2017). Stress-triggered phase separation is an adaptive, evolutionarily tuned response. Cell.

[bib26] Rieckher M, Markaki M, Princz A, Schumacher B, Tavernarakis N (2018). Maintenance of Proteostasis by P Body-Mediated Regulation of eIF4E Availability during Aging in *Caenorhabditis elegans*. Cell Reports.

[bib27] Rogers AN, Chen D, McColl G, Czerwieniec G, Felkey K, Gibson BW, Hubbard A, Melov S, Lithgow GJ, Kapahi P (2011). Life span extension via eIF4G inhibition is mediated by posttranscriptional remodeling of stress response gene expression in *C. elegans*. Cell Metabolism.

[bib28] Santagata S, Mendillo ML, Tang Y, Subramanian A, Perley CC, Roche SP, Wong B, Narayan R, Kwon H, Koeva M, Amon A, Golub TR, Porco JA, Whitesell L, Lindquist S (2013). Tight coordination of protein translation and HSF1 activation supports the anabolic malignant state. Science.

[bib29] Schrader J, Henneberg F, Mata RA, Tittmann K, Schneider TR, Stark H, Bourenkov G, Chari A (2016). The inhibition mechanism of human 20S proteasomes enables next-generation inhibitor design. Science.

[bib30] Schubert D, Currais A, Goldberg J, Finley K, Petrascheck M, Maher P (2018). Geroneuroprotectors: Effective geroprotectors for the brain. Trends in Pharmacological Sciences.

[bib31] Seo K, Choi E, Lee D, Jeong DE, Jang SK, Lee SJ (2013). Heat shock factor 1 mediates the longevity conferred by inhibition of TOR and insulin/IGF-1 signaling pathways in *C. elegans*. Aging Cell.

[bib32] Simonsen A, Cumming RC, Brech A, Isakson P, Schubert DR, Finley KD (2008). Promoting basal levels of autophagy in the nervous system enhances longevity and oxidant resistance in adult *Drosophila*. Autophagy.

[bib33] Solis GM, Petrascheck M (2011). Measuring *Caenorhabditis elegans* life span in 96 well microtiter plates. Journal of Visualized Experiments.

[bib34] Solis GM, Kardakaris R, Valentine ER, Bar-Peled L, Chen AL, Blewett MM, McCormick MA, Williamson JR, Kennedy B, Cravatt BF, Petrascheck M (2018). Translation attenuation by minocycline enhances longevity and proteostasis in old post-stress-responsive organisms. eLife.

[bib35] Steffen KK, Dillin A (2016). A ribosomal perspective on proteostasis and aging. Cell Metabolism.

[bib36] Takauji Y, Wada T, Takeda A, Kudo I, Miki K, Fujii M, Ayusawa D (2016). Restriction of protein synthesis abolishes senescence features at cellular and organismal levels. Scientific Reports.

[bib37] Tarkhov AE, Alla R, Ayyadevara S, Pyatnitskiy M, Menshikov LI, Shmookler Reis RJ, Fedichev PO (2019). A universal transcriptomic signature of age reveals the temporal scaling of *Caenorhabditis elegans* aging trajectories. Scientific Reports.

[bib38] Tye BW, Churchman LS (2021). Hsf1 activation by proteotoxic stress requires concurrent protein synthesis. Molecular Biology of the Cell.

[bib39] Vo M-N, Terrey M, Lee JW, Roy B, Moresco JJ, Sun L, Fu H, Liu Q, Weber TG, Yates JR, Fredrick K, Schimmel P, Ackerman SL (2018). ANKRD16 prevents neuron loss caused by an editing-defective tRNA synthetase. Nature.

[bib40] Xu G, Pattamatta A, Hildago R, Pace MC, Brown H, Borchelt DR (2016). Vulnerability of newly synthesized proteins to proteostasis stress. Journal of Cell Science.

[bib41] Yonashiro R, Tahara EB, Bengtson MH, Khokhrina M, Lorenz H, Chen K-C, Kigoshi-Tansho Y, Savas JN, Yates JR, Kay SA, Craig EA, Mogk A, Bukau B, Joazeiro CAP (2016). The Rqc2/Tae2 subunit of the ribosome-associated quality control (RQC) complex marks ribosome-stalled nascent polypeptide chains for aggregation. eLife.

[bib42] Zhao J, Zhai B, Gygi SP, Goldberg AL (2015). mTOR inhibition activates overall protein degradation by the ubiquitin proteasome system as well as by autophagy. PNAS.

[bib43] Zhou C, Slaughter BD, Unruh JR, Guo F, Yu Z, Mickey K, Narkar A, Ross RT, McClain M, Li R (2014). Organelle-based aggregation and retention of damaged proteins in asymmetrically dividing cells. Cell.

