# Supplementary material for: Proteostasis is differentially modulated by inhibition of translation initiation or elongation
Source: eLife. 2023 Oct 5;12:e76465. doi: 10.7554/eLife.76465 (PMC10581687; doi:10.7554/eLife.76465)
Supplement: Figure 1—source data 1. [file elife-76465-fig1-data1.zip › Figure 1B_source_data/Figure 1B-source data2.pptx]

## Slide 1
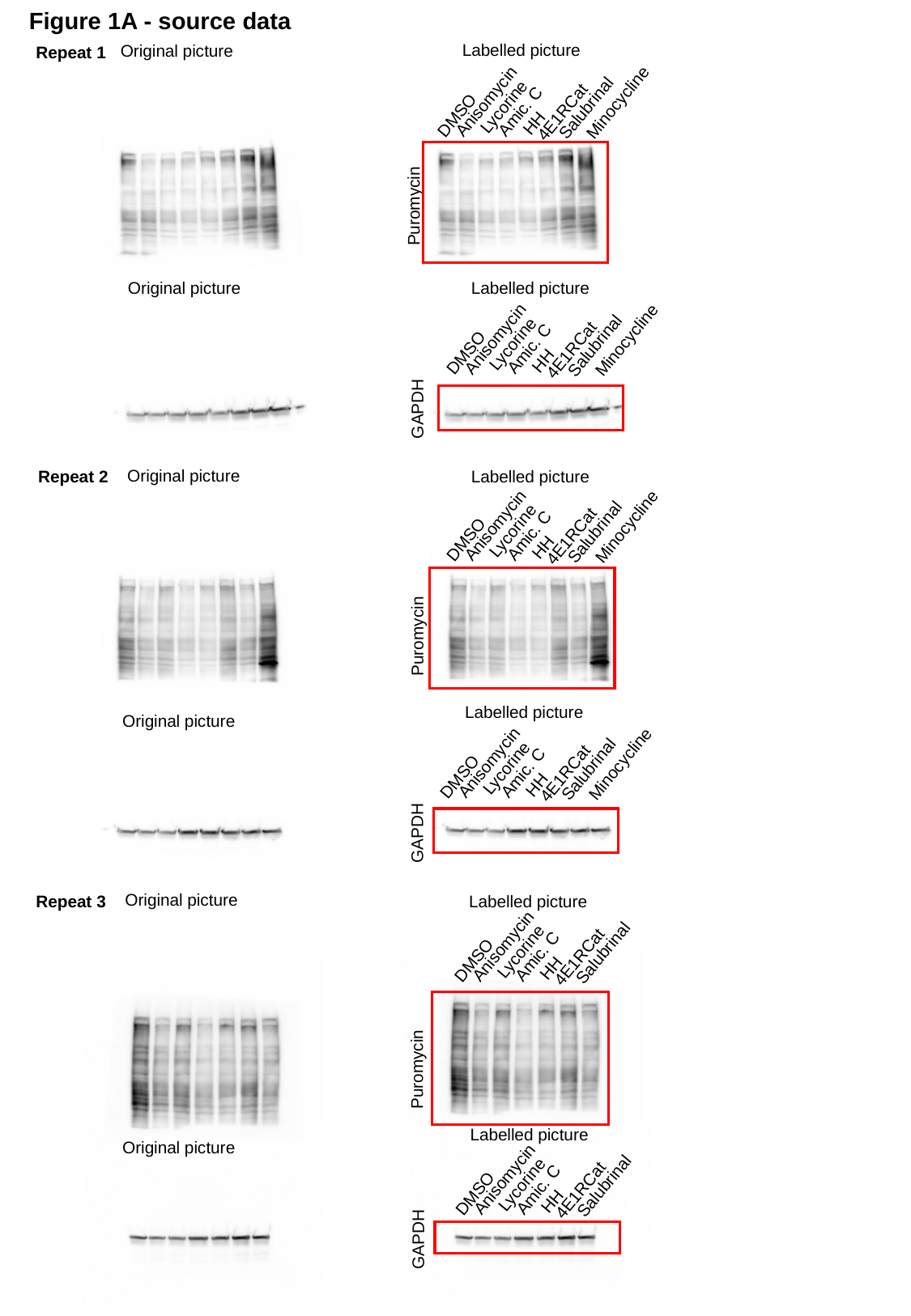

Figure 1A - source data
Labelled picture
Original picture
Repeat 1
Minocycline
Anisomycin
Salubrinal
Lycorine
Amic. C
DMSO
4E1RCat
HH
Puromycin
Original picture
Labelled picture
Minocycline
Anisomycin
Salubrinal
Lycorine
Amic. C
DMSO
4E1RCat
HH
GAPDH
Original picture
Repeat 2
Labelled picture
Minocycline
Anisomycin
Salubrinal
Lycorine
Amic. C
DMSO
4E1RCat
HH
Puromycin
Labelled picture
Original picture
Minocycline
Anisomycin
Salubrinal
Lycorine
Amic. C
DMSO
4E1RCat
HH
GAPDH
Original picture
Repeat 3
Labelled picture
Anisomycin
Salubrinal
Lycorine
Amic. C
DMSO
4E1RCat
HH
Puromycin
Labelled picture
Original picture
Anisomycin
Salubrinal
Lycorine
Amic. C
DMSO
4E1RCat
HH
GAPDH
