# Supplementary material for: Proteostasis is differentially modulated by inhibition of translation initiation or elongation
Source: eLife. 2023 Oct 5;12:e76465. doi: 10.7554/eLife.76465 (PMC10581687; doi:10.7554/eLife.76465)

Figure 1A - source data

Repeat 1 Original picture

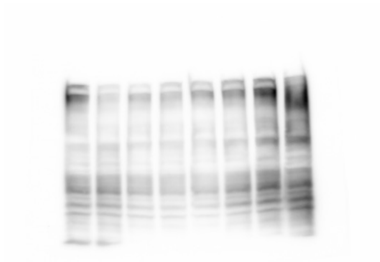

Labelled picture

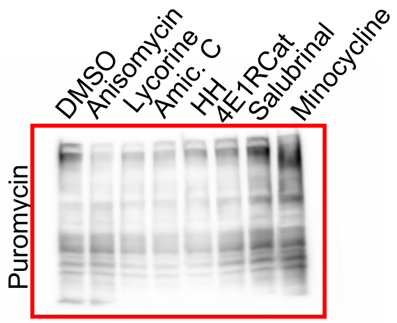

Original picture

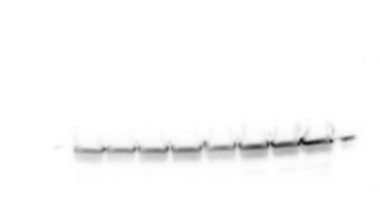

Labelled picture

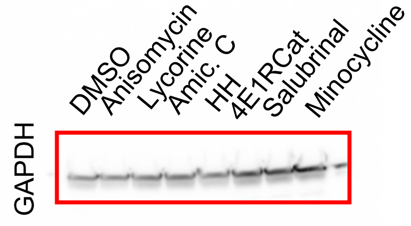

Repeat 2 Original picture

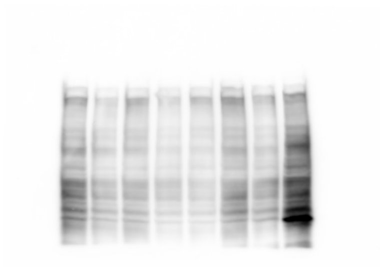

Labelled picture

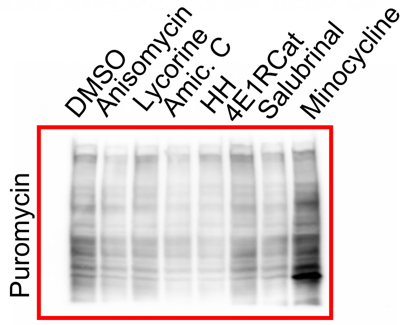

Original picture

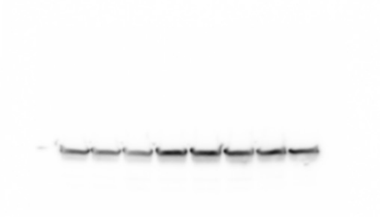

Labelled picture

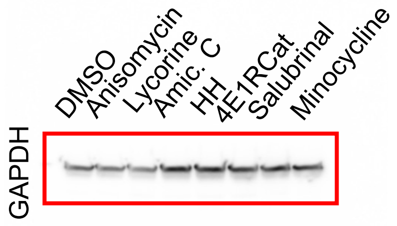

Repeat 3 Original picture

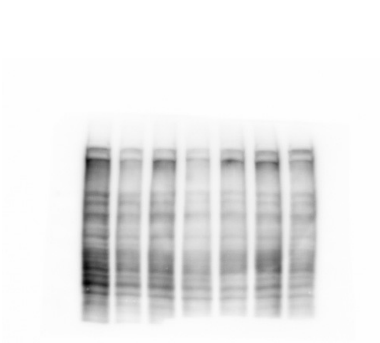

Labelled picture

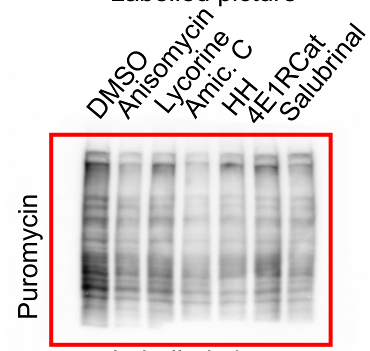

Original picture

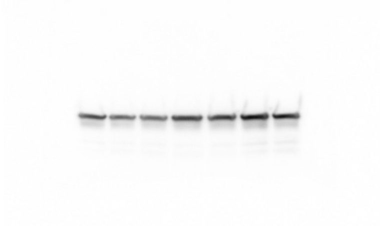

Labelled picture

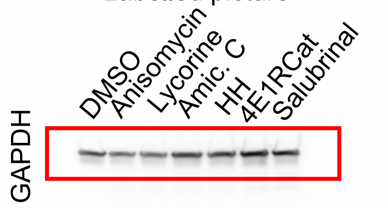

Supplement: Figure 1—source data 1. [file elife-76465-fig1-data1.zip › Figure 1B_source_data/Figure 1B-source data1.pdf]
