# Supplementary figures and images for: Proteostasis is differentially modulated by inhibition of translation initiation or elongation
Source: eLife. 2023 Oct 5;12:e76465. doi: 10.7554/eLife.76465 (PMC10581687; doi:10.7554/eLife.76465)

## Slide 1
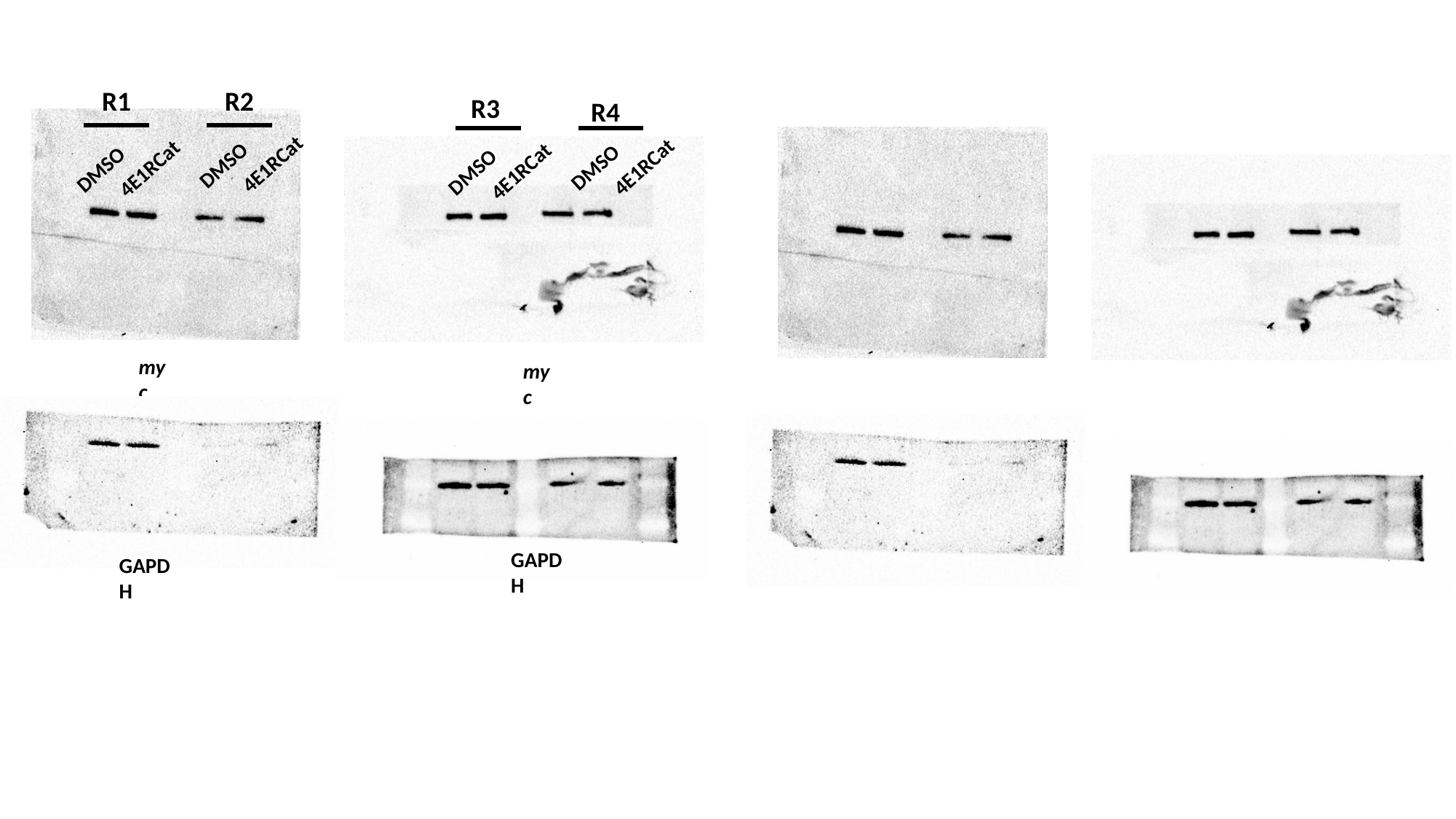

R1
R2
R3
R4
4E1RCat
4E1RCat
DMSO
DMSO
4E1RCat
DMSO
4E1RCat
DMSO
myc
myc
GAPDH
GAPDH

Supplement: Figure 1—source data 4. [file elife-76465-fig1-data4.zip › Figure 1F_source_data/Figure 1F_source_data3/Inset.pptx]

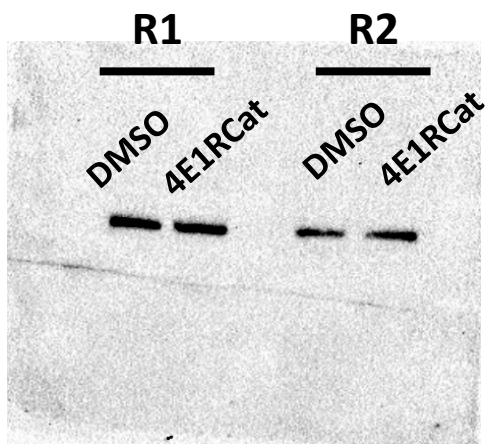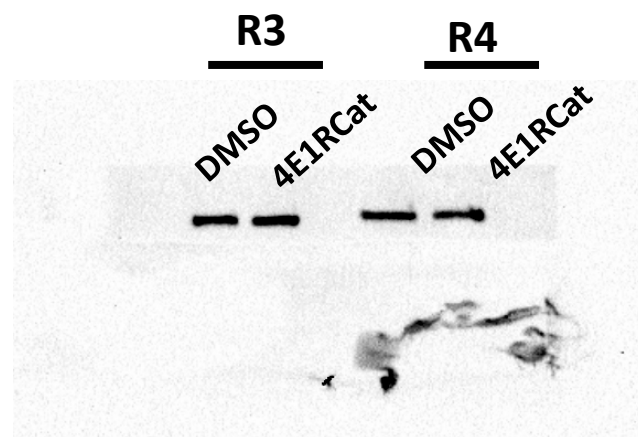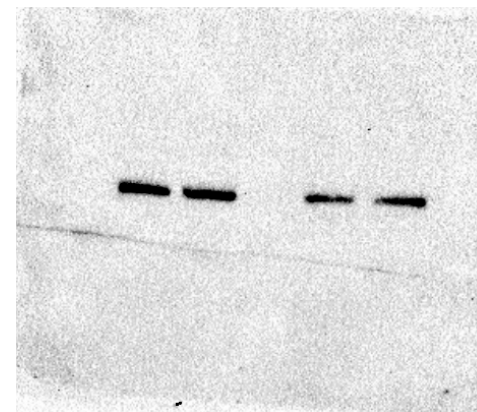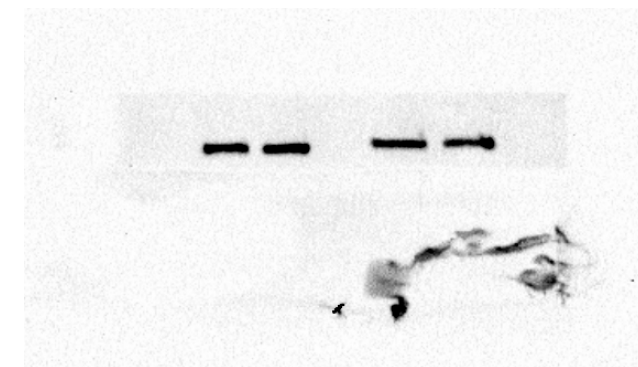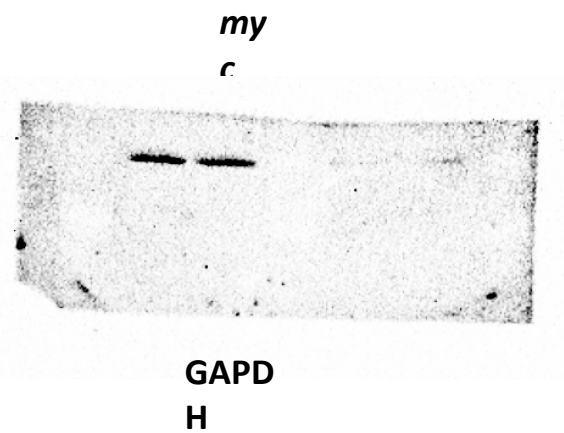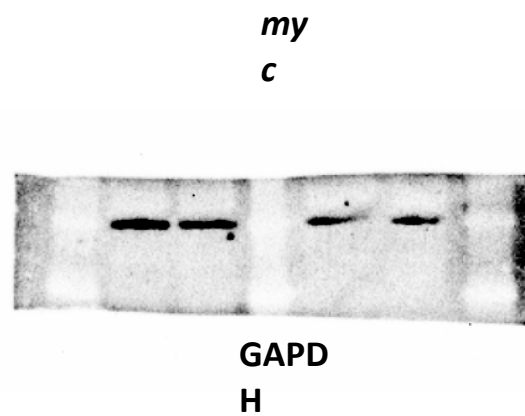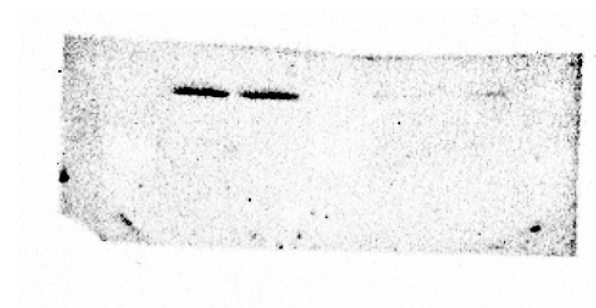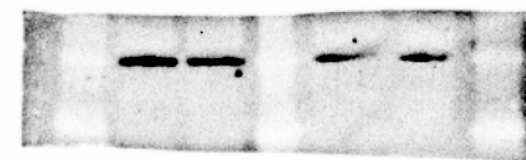

Supplement: Figure 1—source data 4. [file elife-76465-fig1-data4.zip › Figure 1F_source_data/Figure 1F_source_data3/Inset.pdf]

kc-033023\_chx\_puromycin

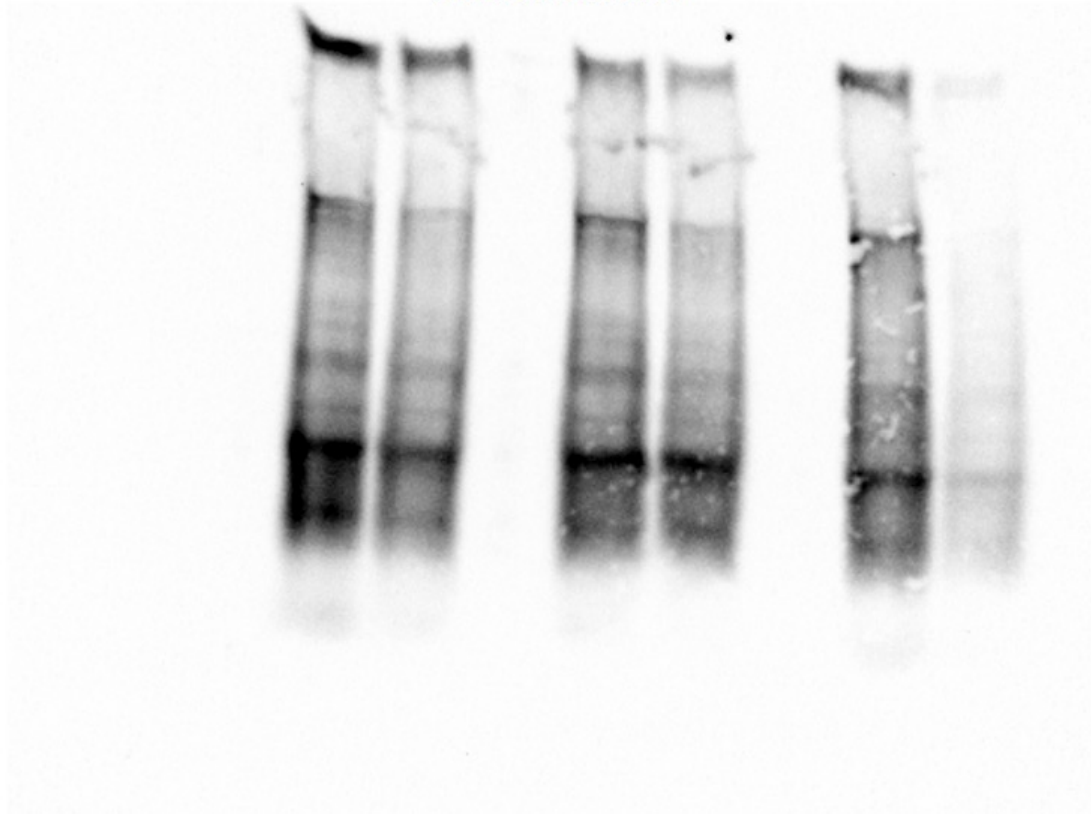

kc\_032923\_chx\_GAPDH

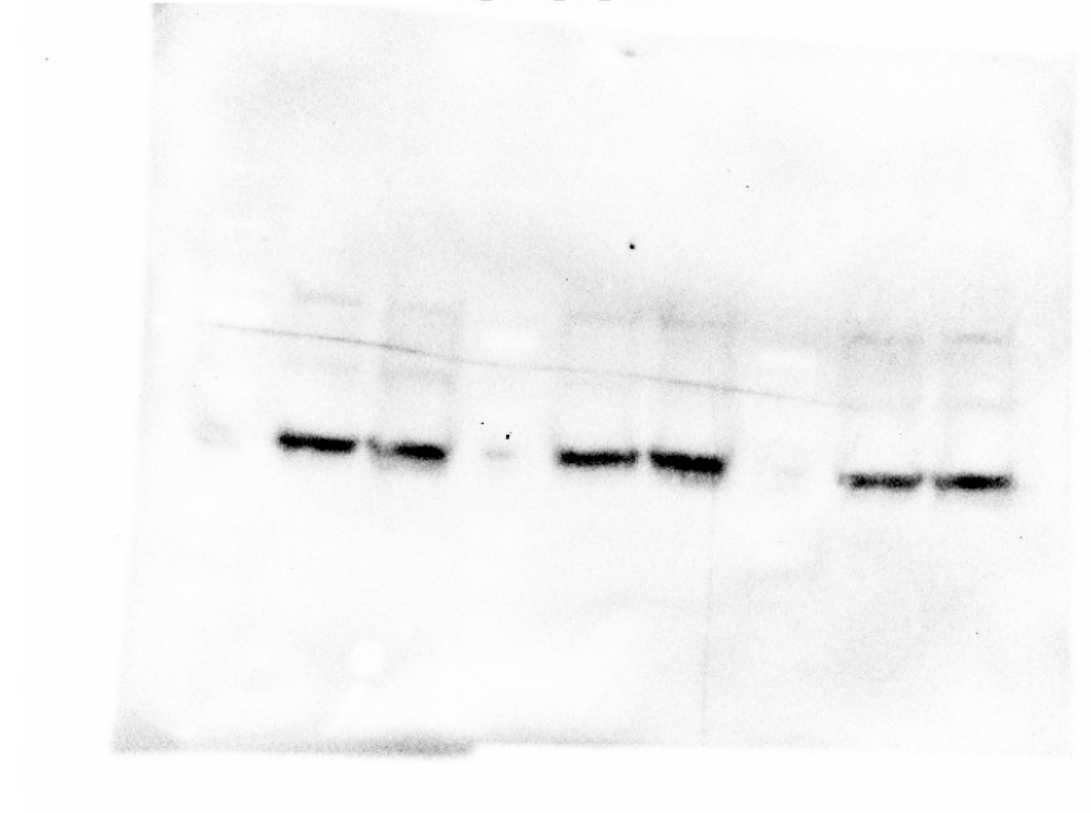

Supplement: Figure 1—figure supplement 1—source data 1. [file elife-76465-fig1-figsupp1-data1.zip › Figure 1ΓÇöFigure Supplement 1A-source data/Figure1ΓÇöFigure Supplement A-source data1.pdf]

## Slide 1
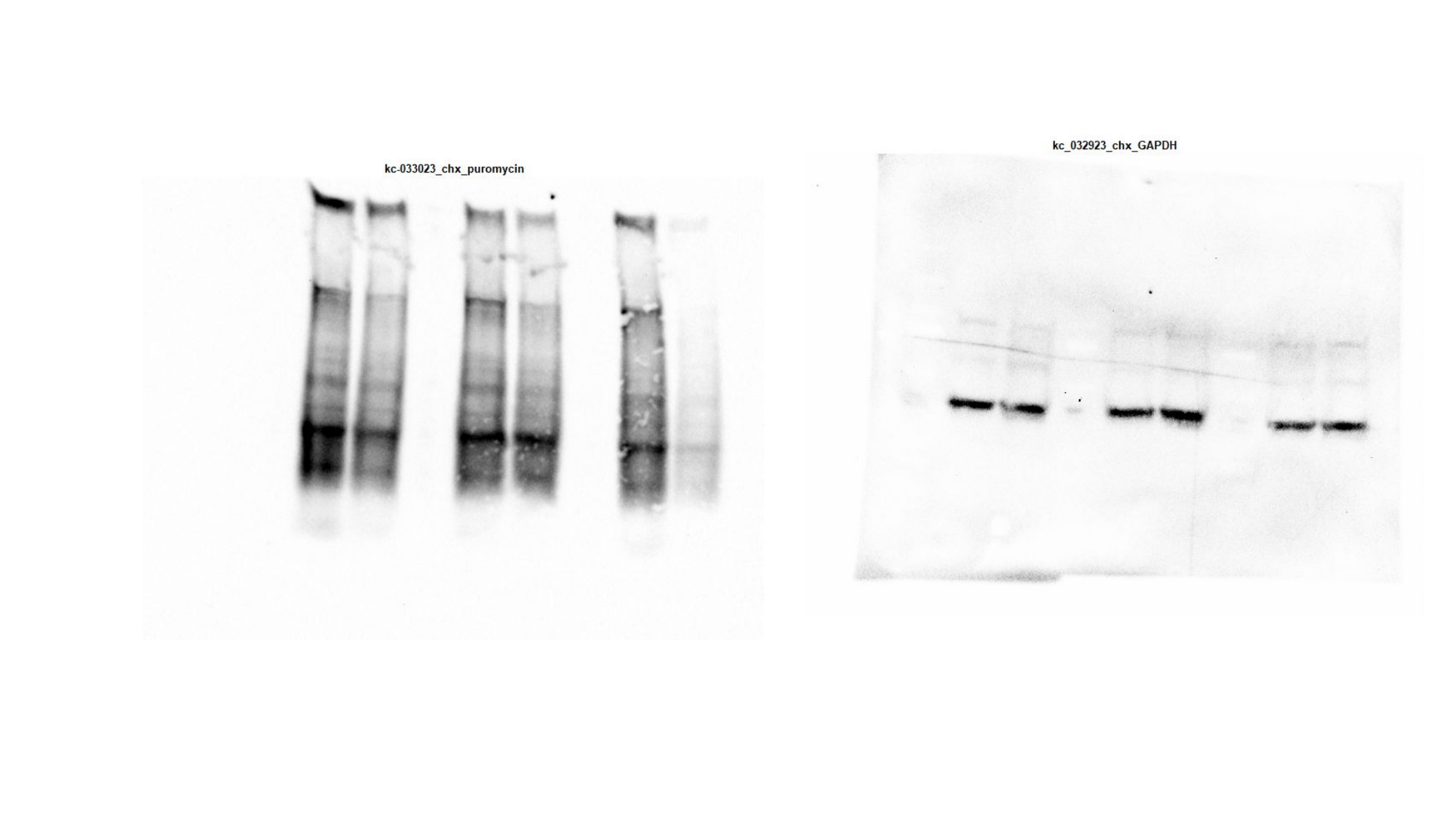

Supplement: Figure 1—figure supplement 1—source data 1. [file elife-76465-fig1-figsupp1-data1.zip › Figure 1ΓÇöFigure Supplement 1A-source data/Figure1ΓÇöFigure Supplement A-source data2.pptx]

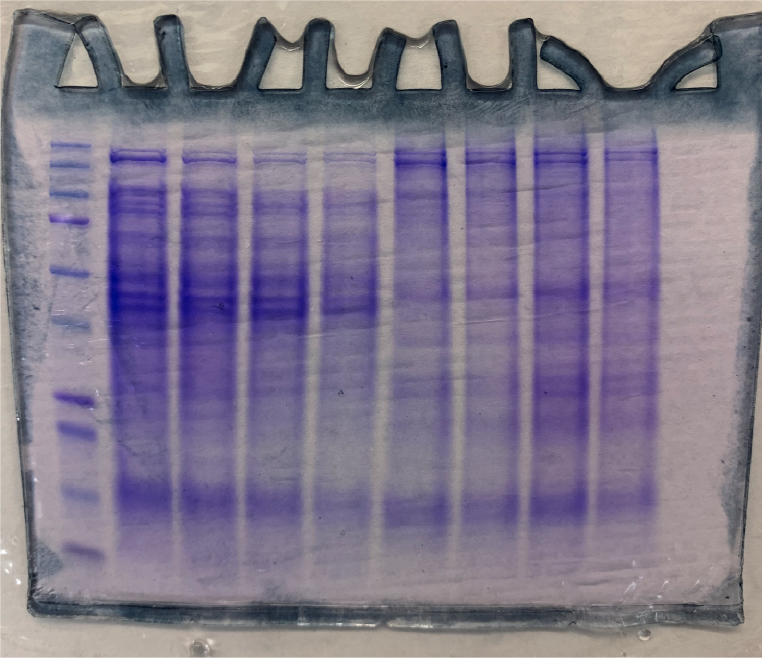

Supplement: Figure 2—source data 3. [file elife-76465-fig2-data3.zip › Figure 2D_source_data/Figure 2D_uncropped.png]

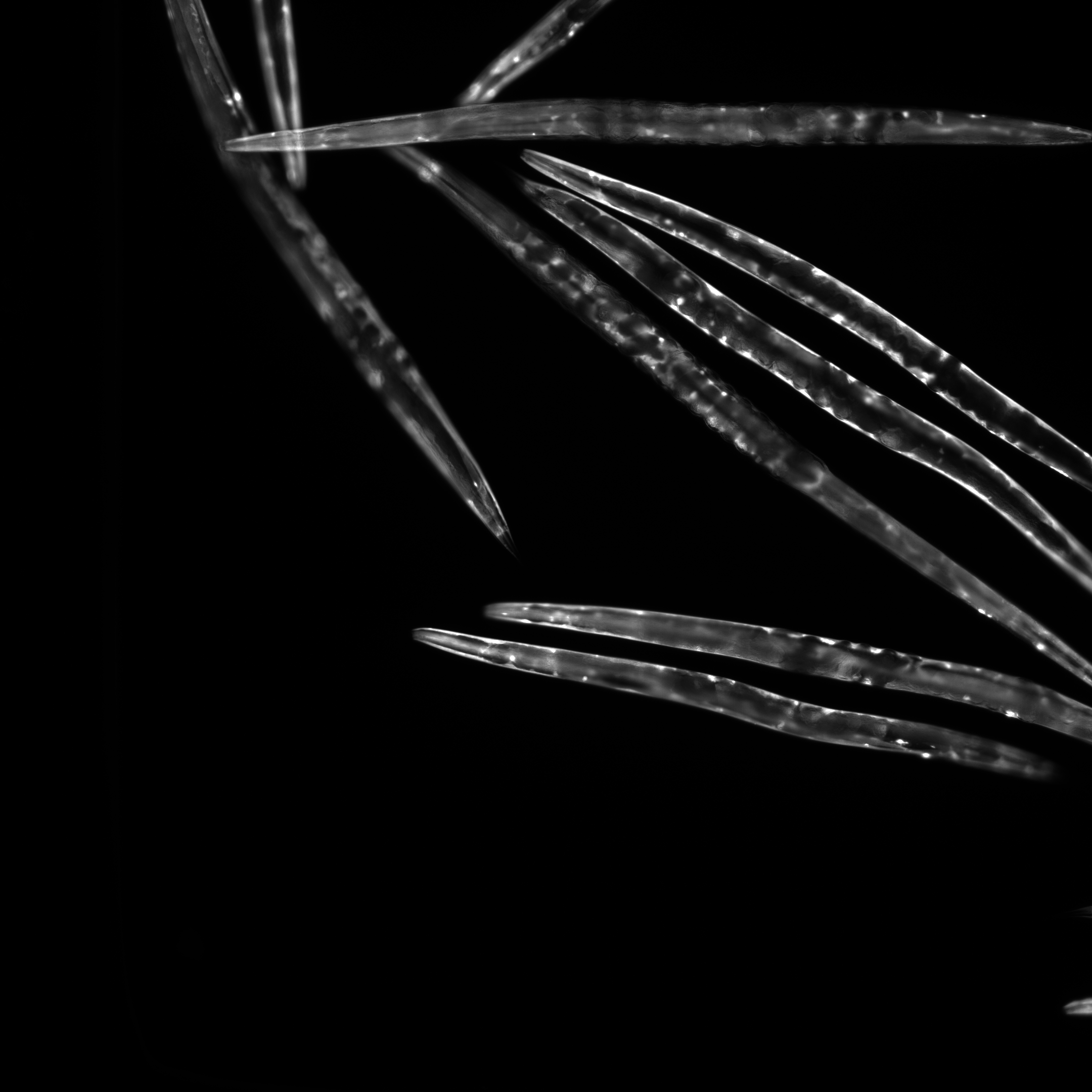

Supplement: Figure 3—source data 1. [file elife-76465-fig3-data1.zip › Figure 3B_source_data/D05_S3_5.tif]

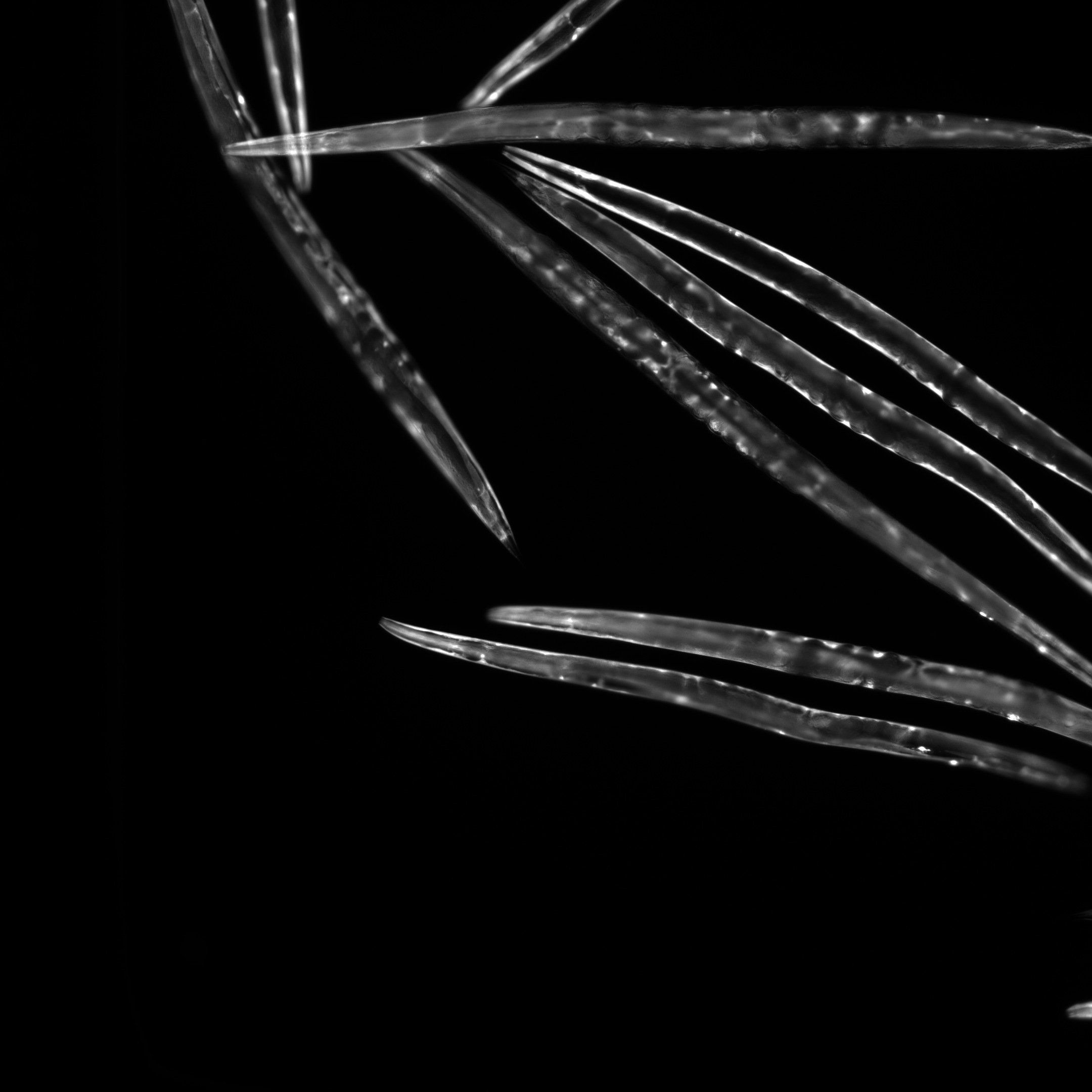

Supplement: Figure 3—source data 1. [file elife-76465-fig3-data1.zip › Figure 3B_source_data/D05_S3_1.tif]

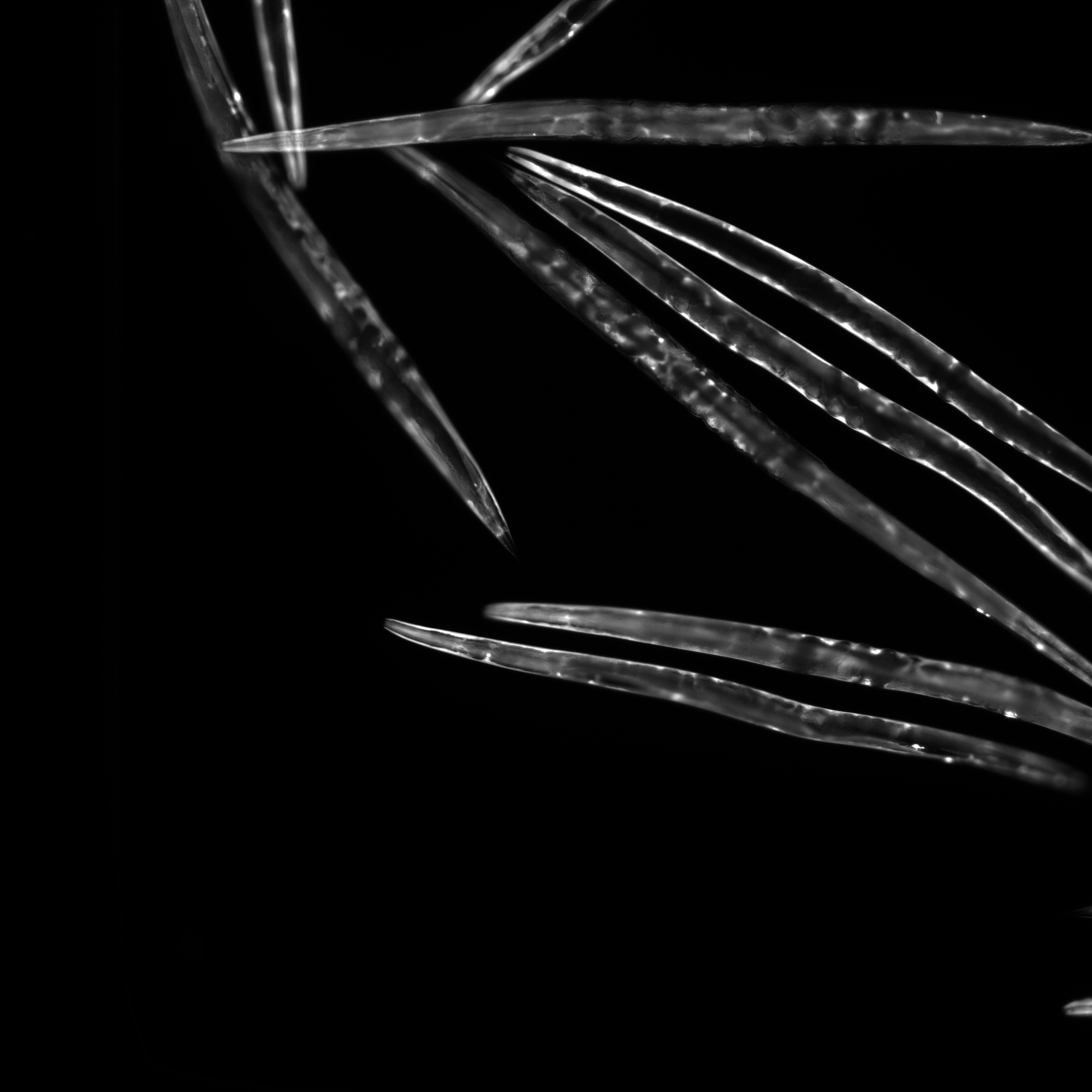

Supplement: Figure 3—source data 1. [file elife-76465-fig3-data1.zip › Figure 3B_source_data/D05_S3_2.tif]

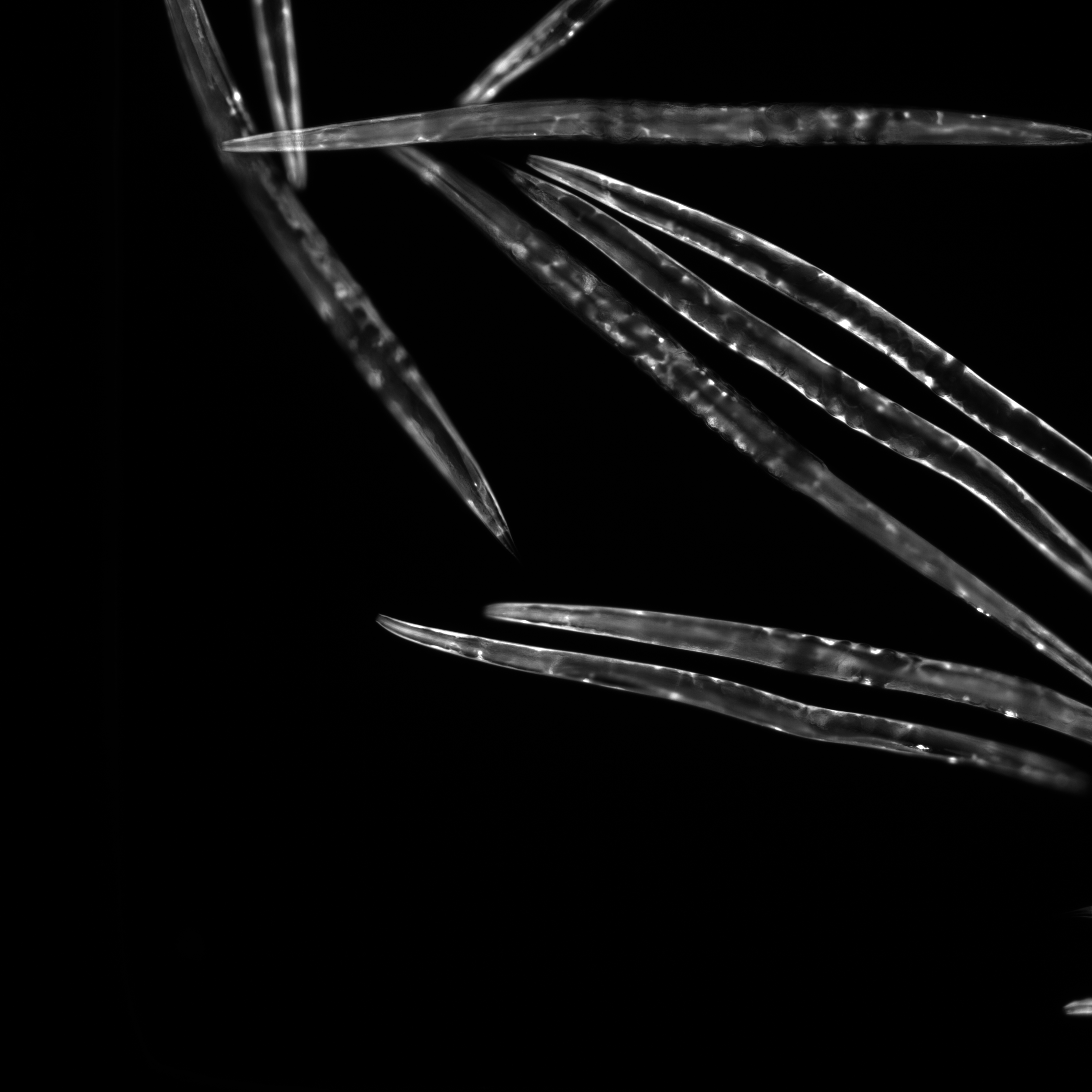

Supplement: Figure 3—source data 1. [file elife-76465-fig3-data1.zip › Figure 3B_source_data/D05_S3_3.tif]

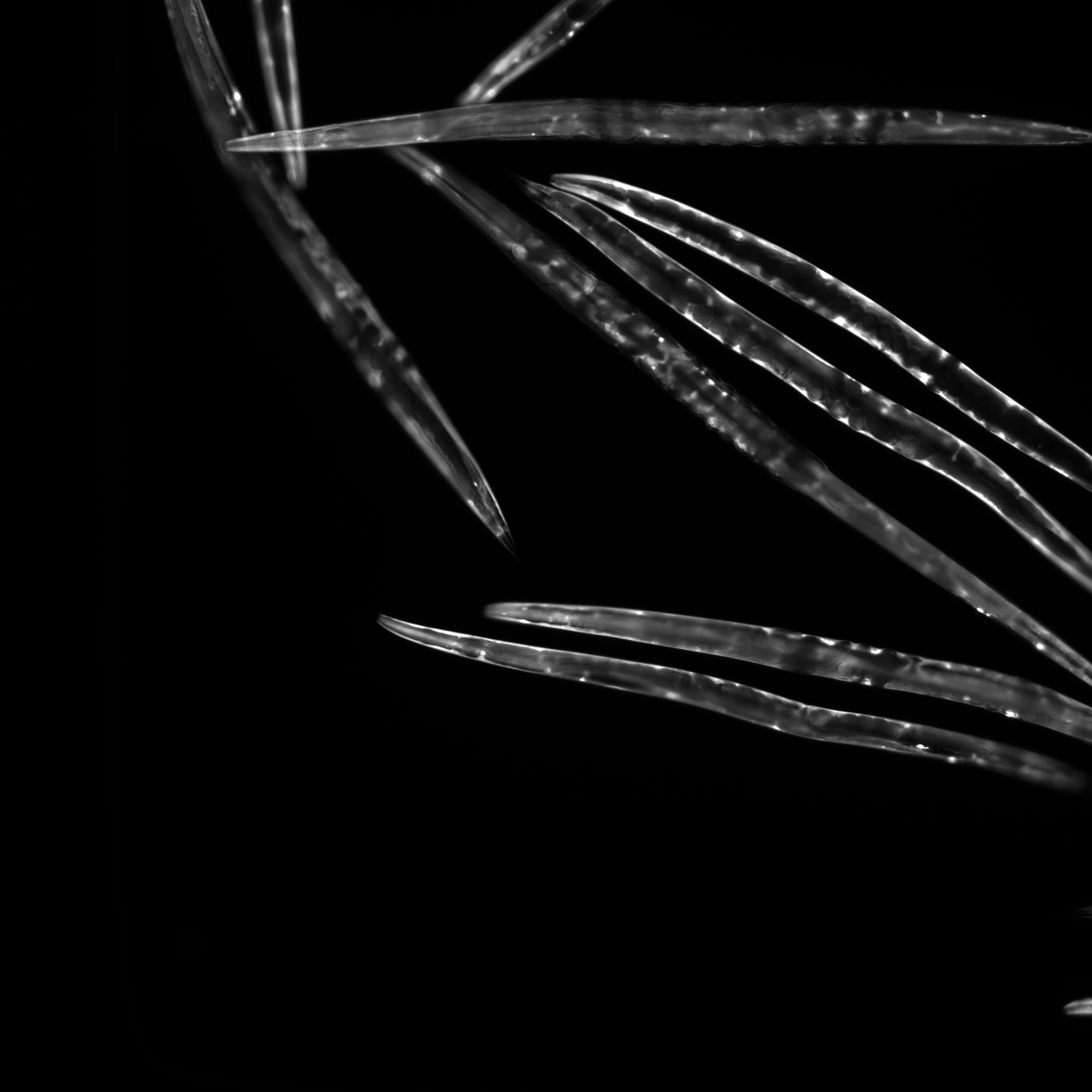

Supplement: Figure 3—source data 1. [file elife-76465-fig3-data1.zip › Figure 3B_source_data/D05_S3_4.tif]

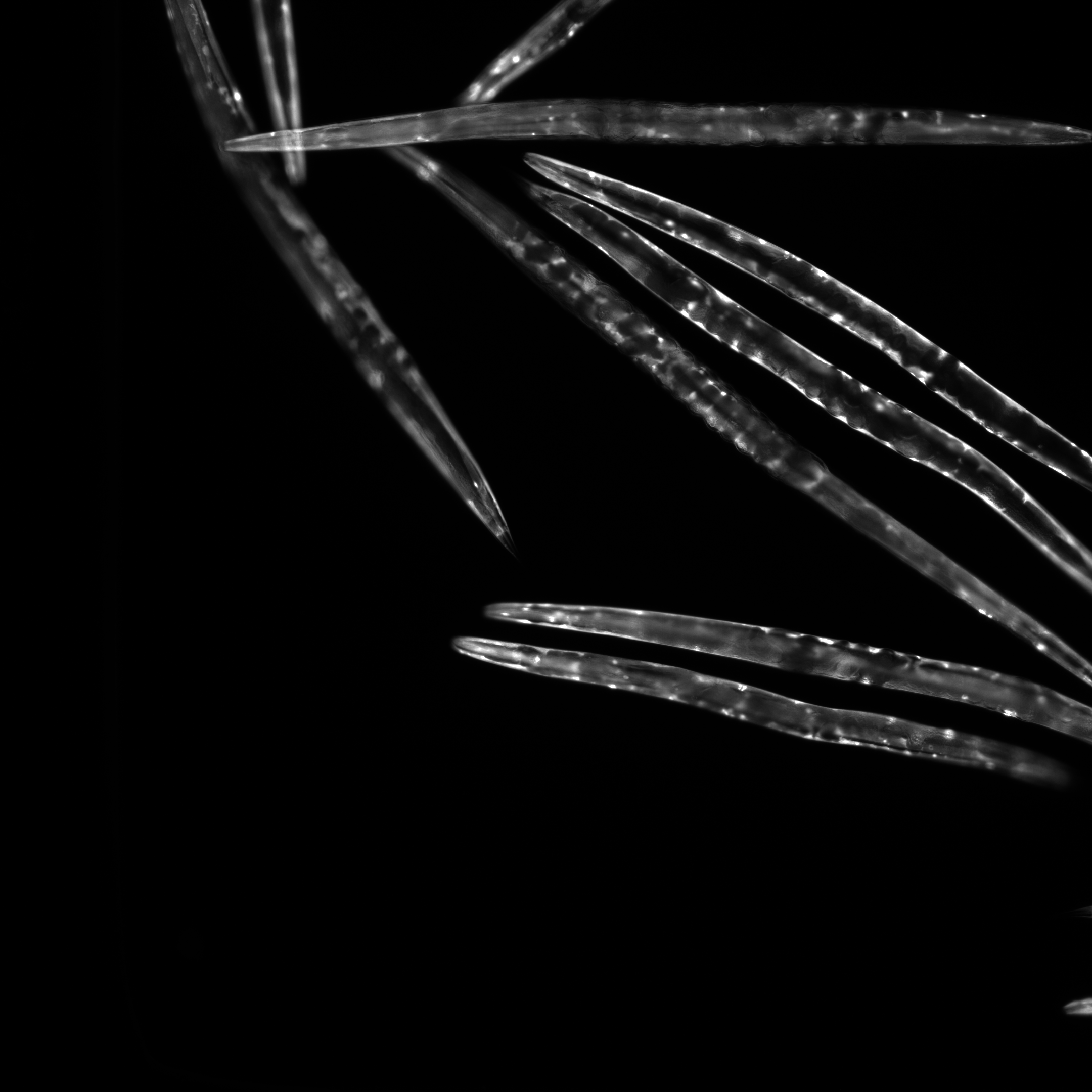

Supplement: Figure 3—source data 1. [file elife-76465-fig3-data1.zip › Figure 3B_source_data/D05_S3_6.tif]

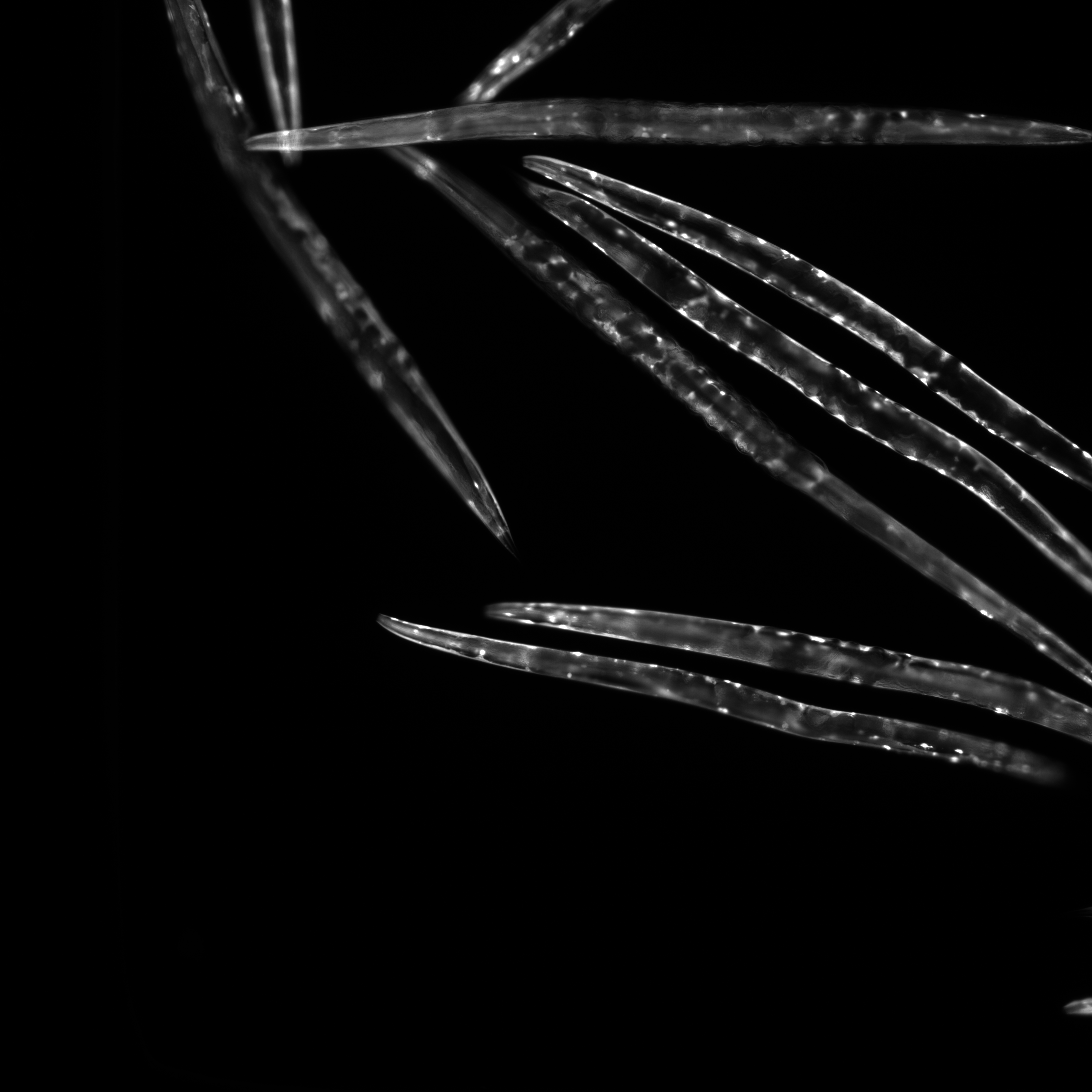

Supplement: Figure 3—source data 1. [file elife-76465-fig3-data1.zip › Figure 3B_source_data/D05_S3_7.tif]

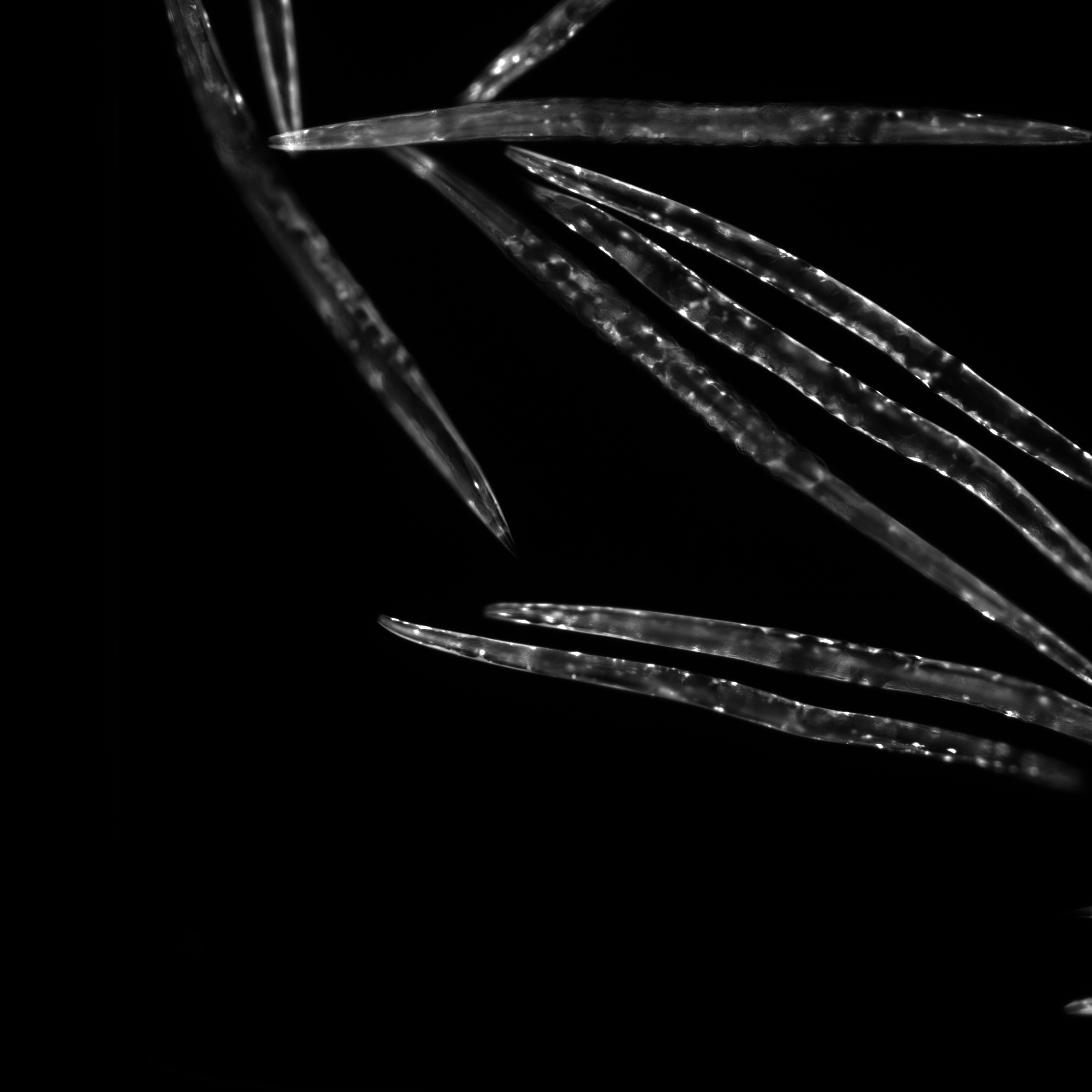

Supplement: Figure 3—source data 1. [file elife-76465-fig3-data1.zip › Figure 3B_source_data/D05_S3_8.tif]

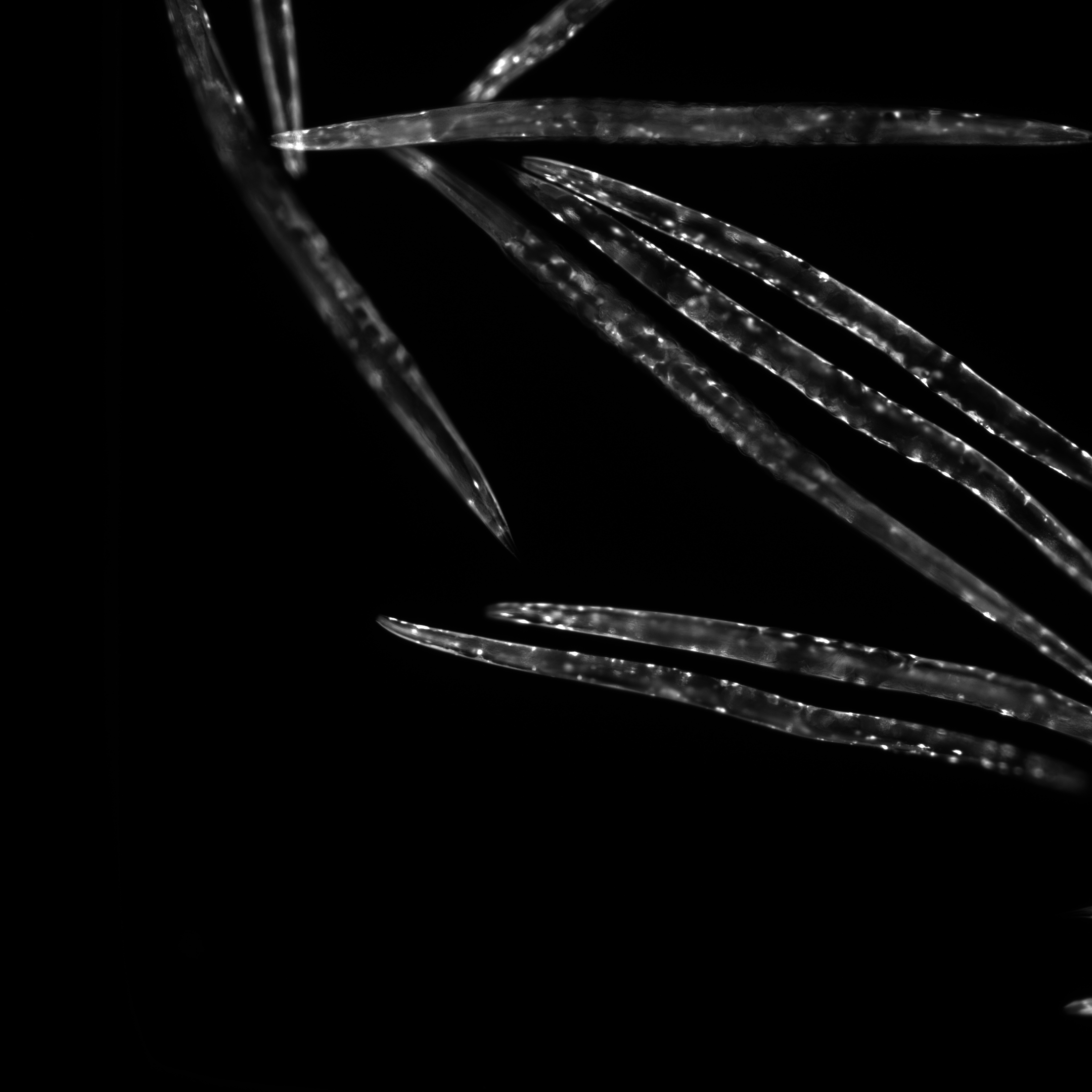

Supplement: Figure 3—source data 1. [file elife-76465-fig3-data1.zip › Figure 3B_source_data/D05_S3_9.tif]

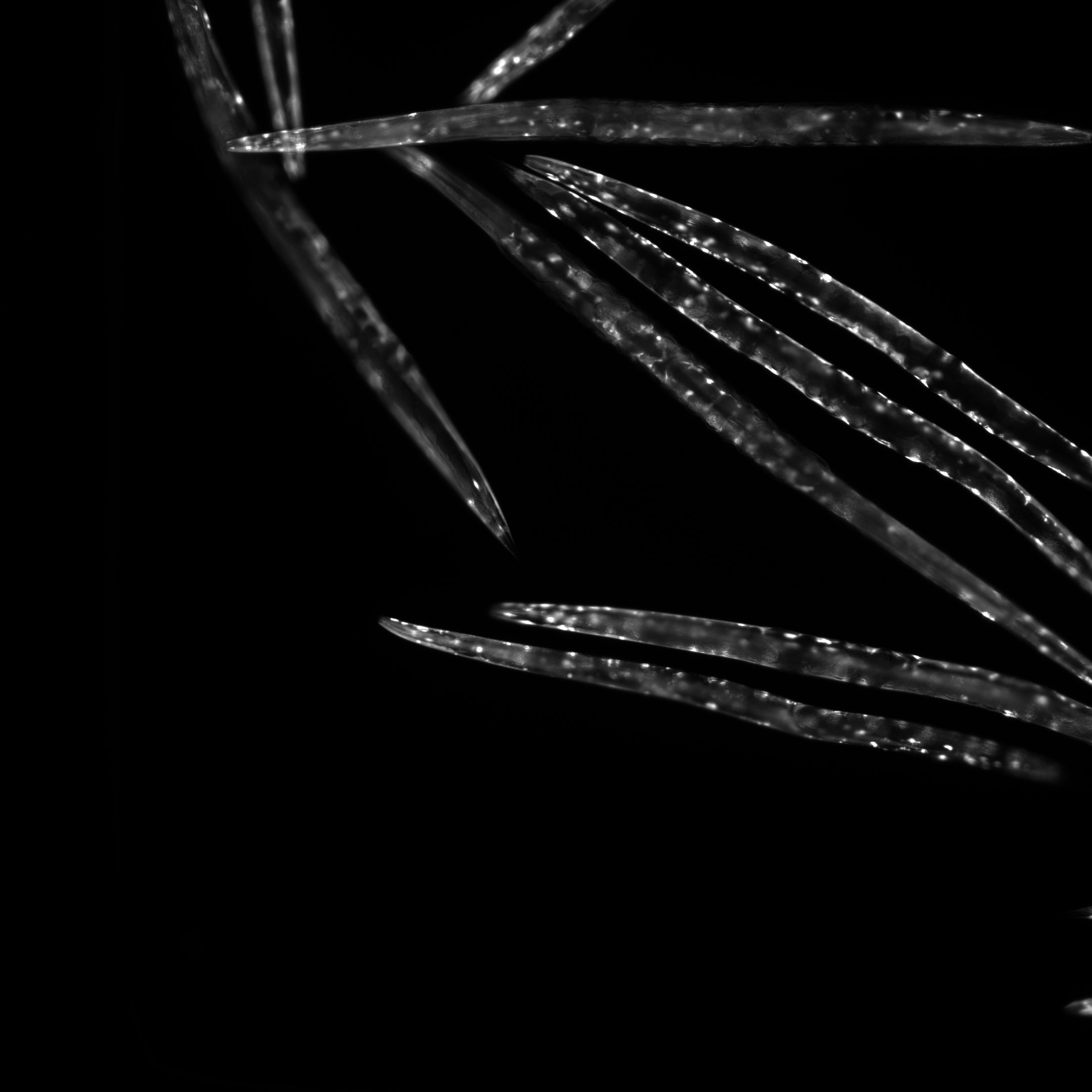

Supplement: Figure 3—source data 1. [file elife-76465-fig3-data1.zip › Figure 3B_source_data/D05_S3_10.tif]

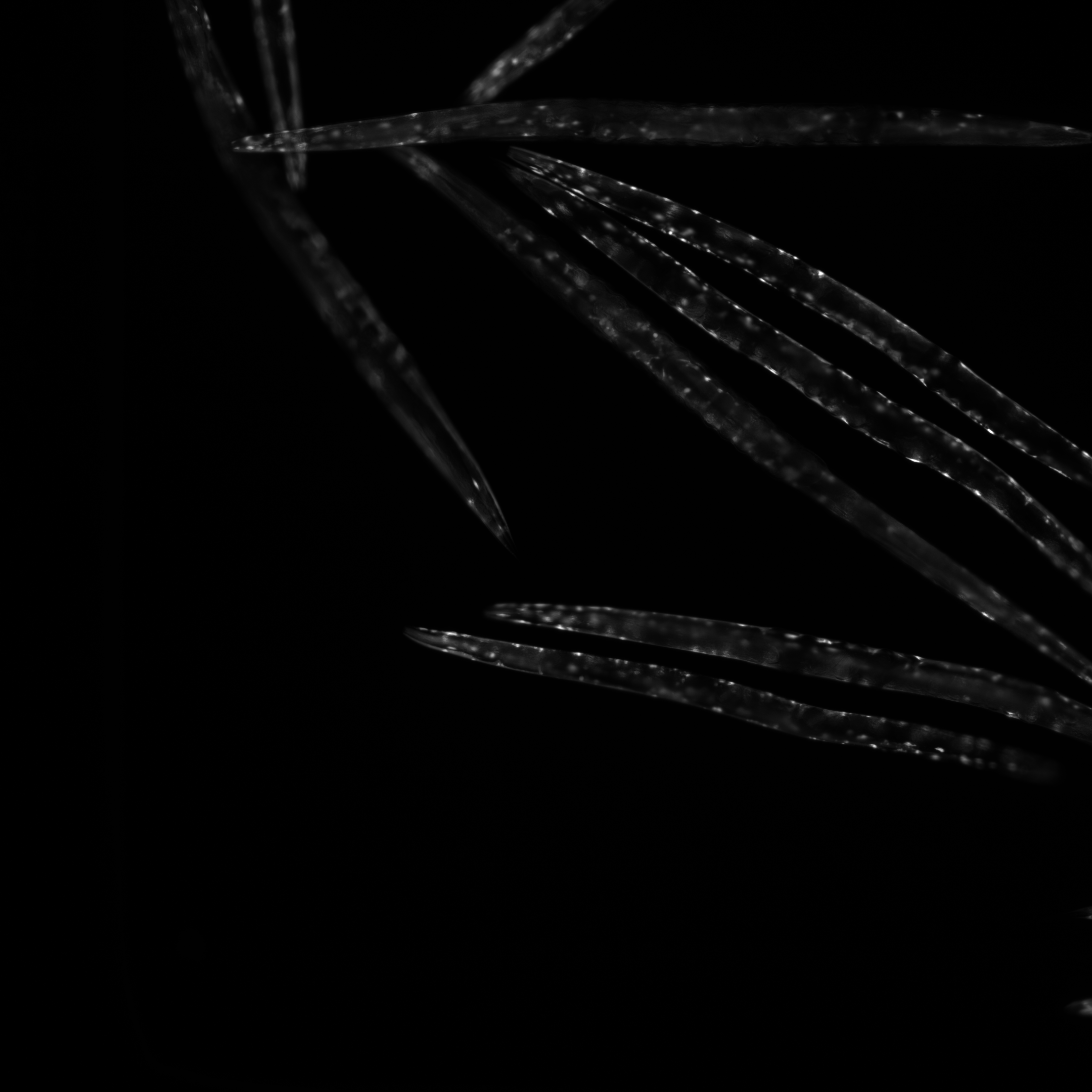

Supplement: Figure 3—source data 1. [file elife-76465-fig3-data1.zip › Figure 3B_source_data/D05_S3_11.tif]

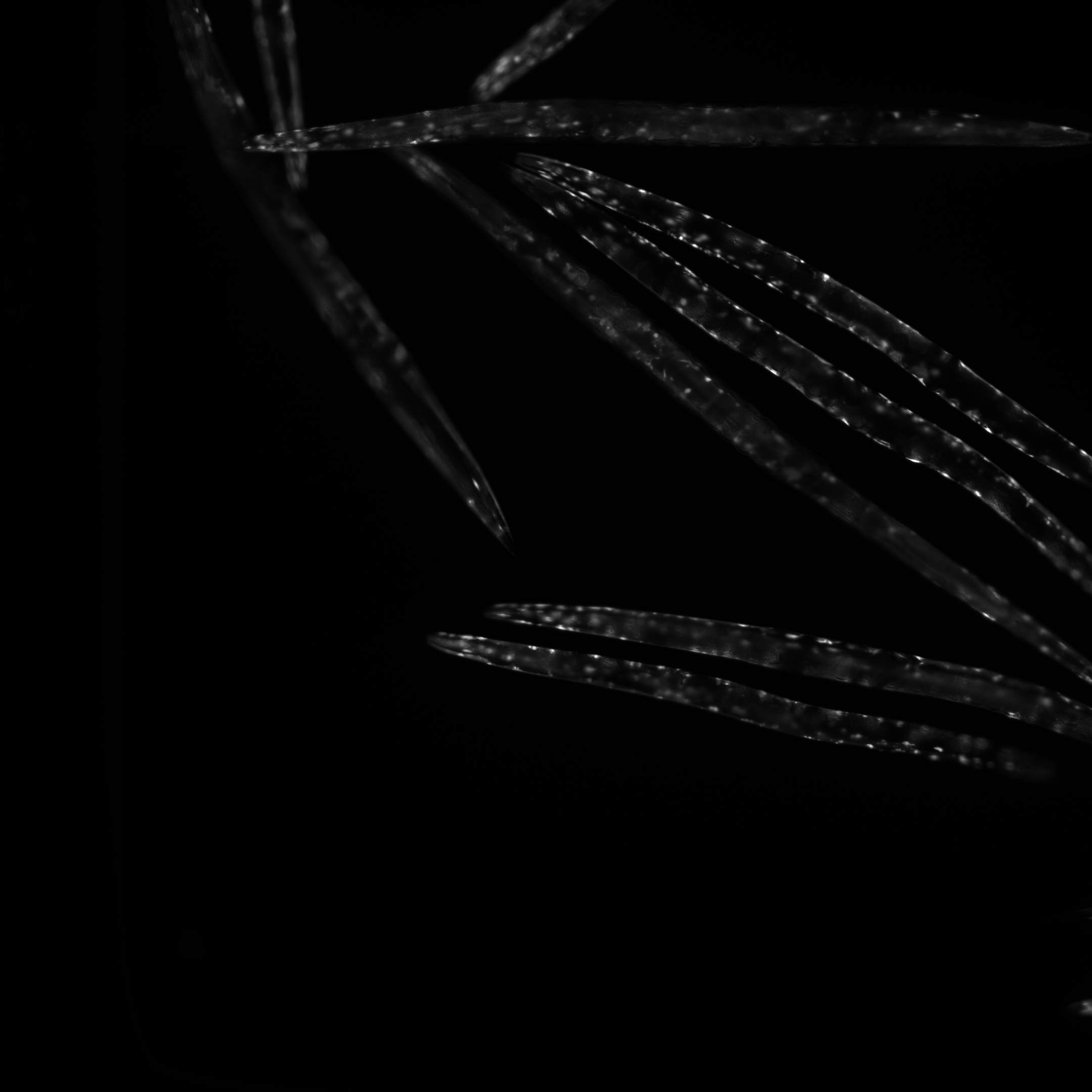

Supplement: Figure 3—source data 1. [file elife-76465-fig3-data1.zip › Figure 3B_source_data/D05_S3_12.tif]

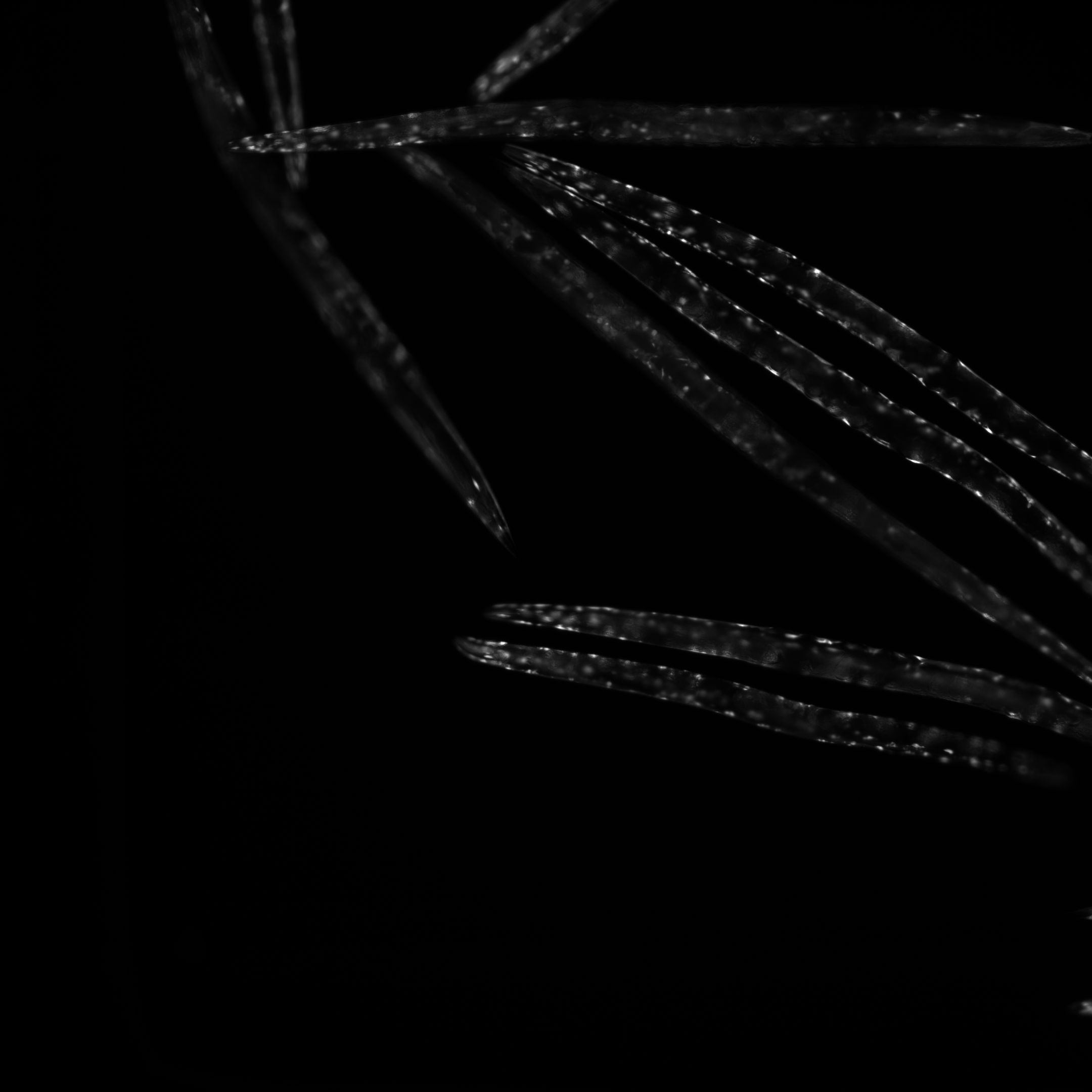

Supplement: Figure 3—source data 1. [file elife-76465-fig3-data1.zip › Figure 3B_source_data/D05_S3_13.tif]

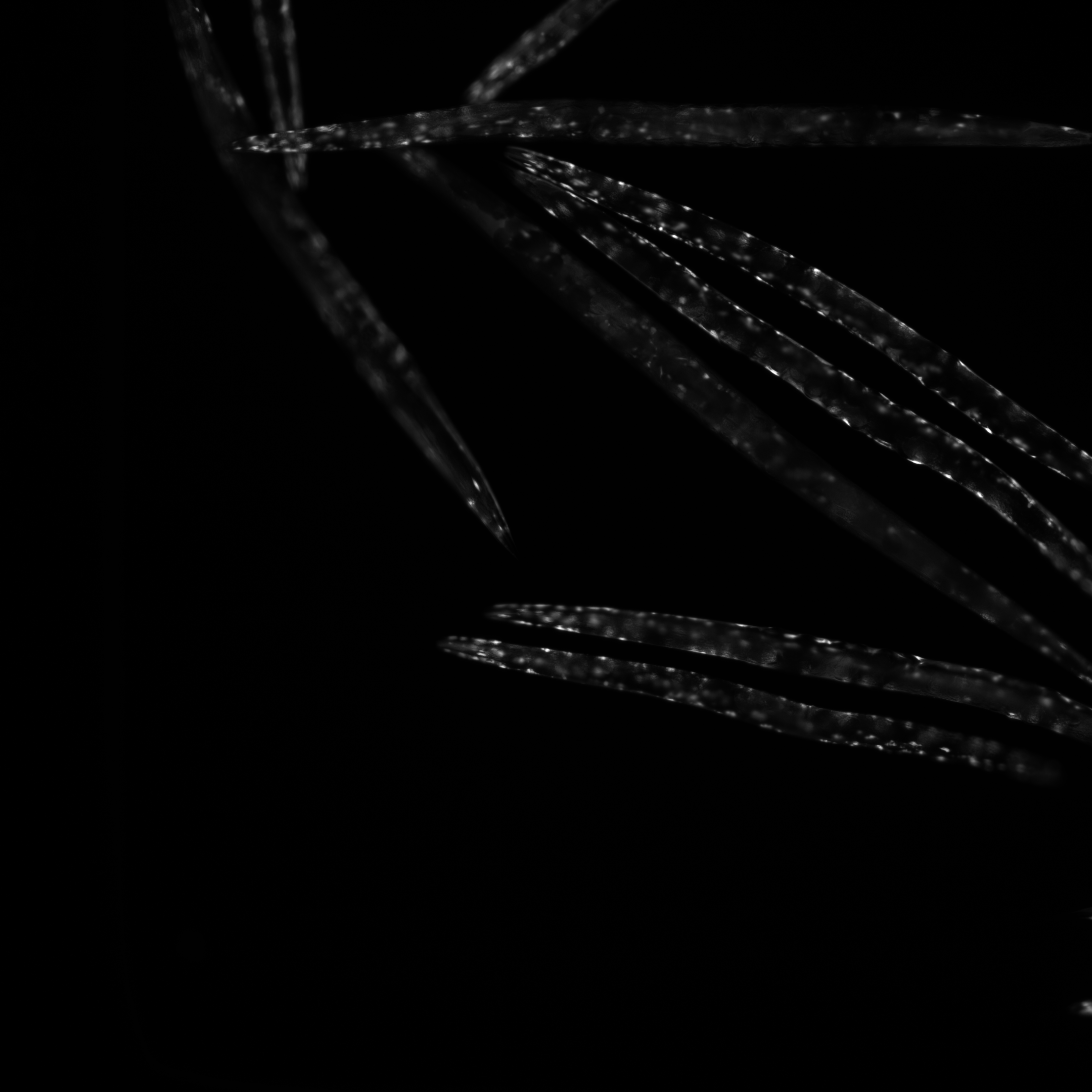

Supplement: Figure 3—source data 1. [file elife-76465-fig3-data1.zip › Figure 3B_source_data/D05_S3_14.tif]

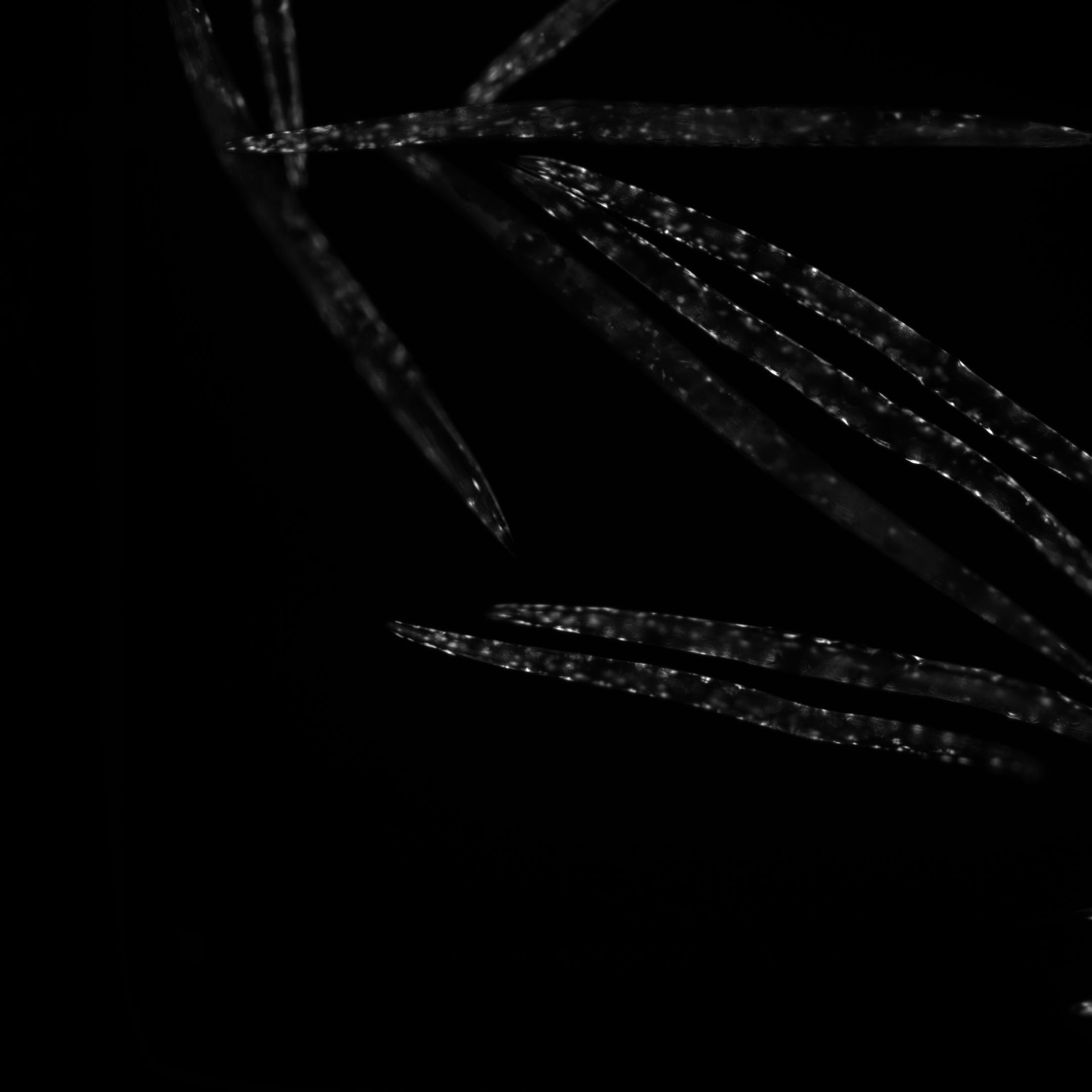

Supplement: Figure 3—source data 1. [file elife-76465-fig3-data1.zip › Figure 3B_source_data/D05_S3_15.tif]

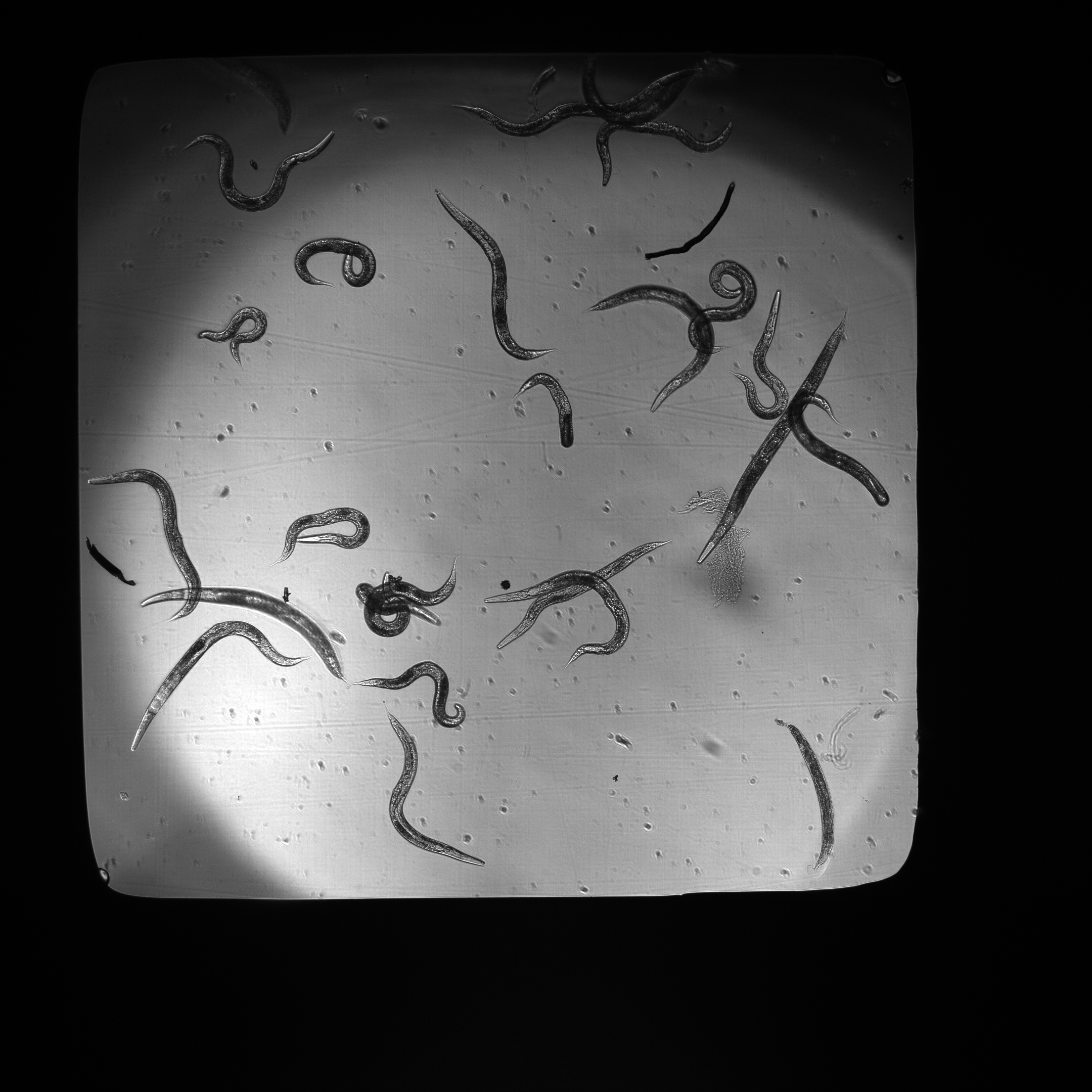

Supplement: Figure 4—source data 2. [file elife-76465-fig4-data2.zip › Figure 4C_source_data/Anis_then_Bort.tif]

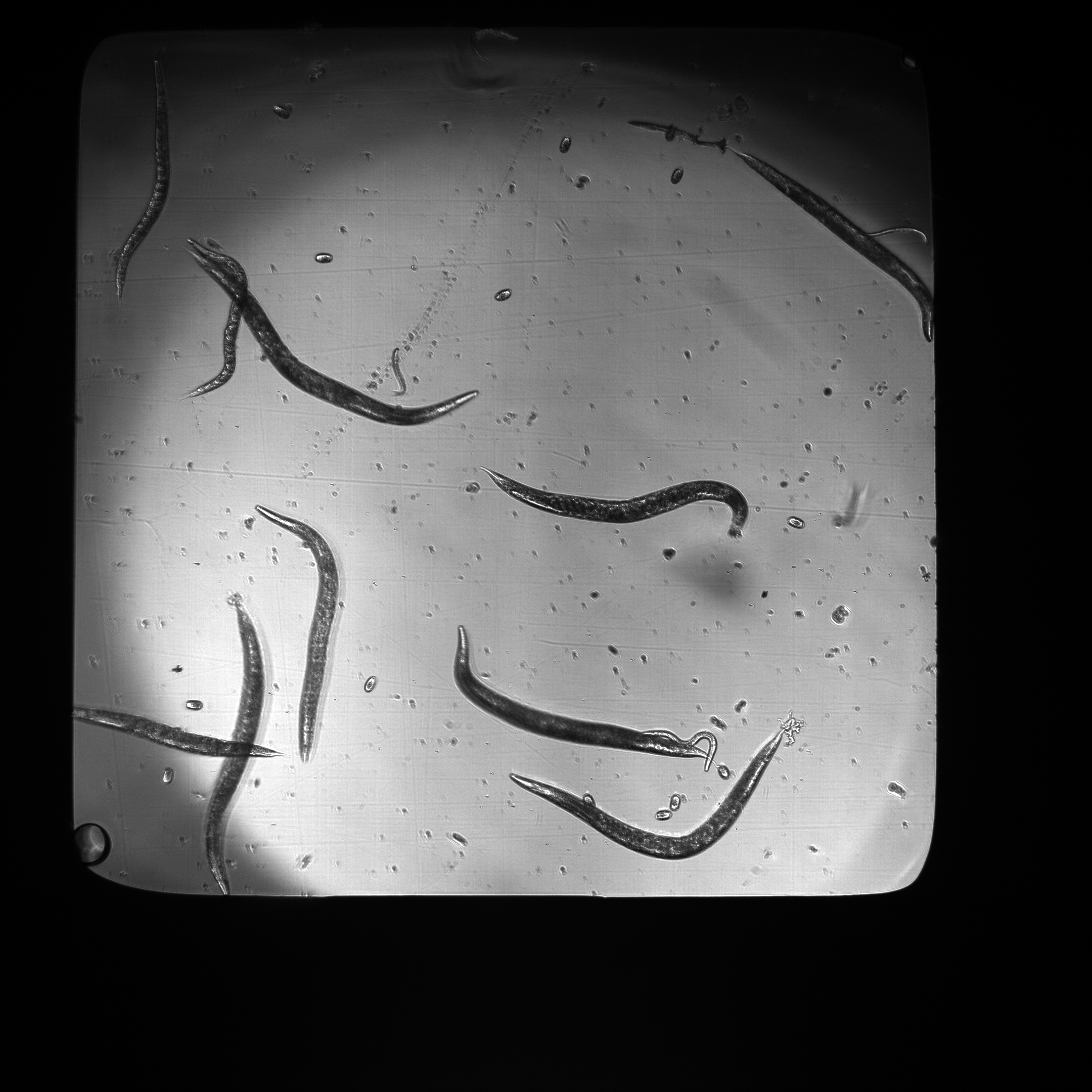

Supplement: Figure 4—source data 2. [file elife-76465-fig4-data2.zip › Figure 4C_source_data/Anis.tif]

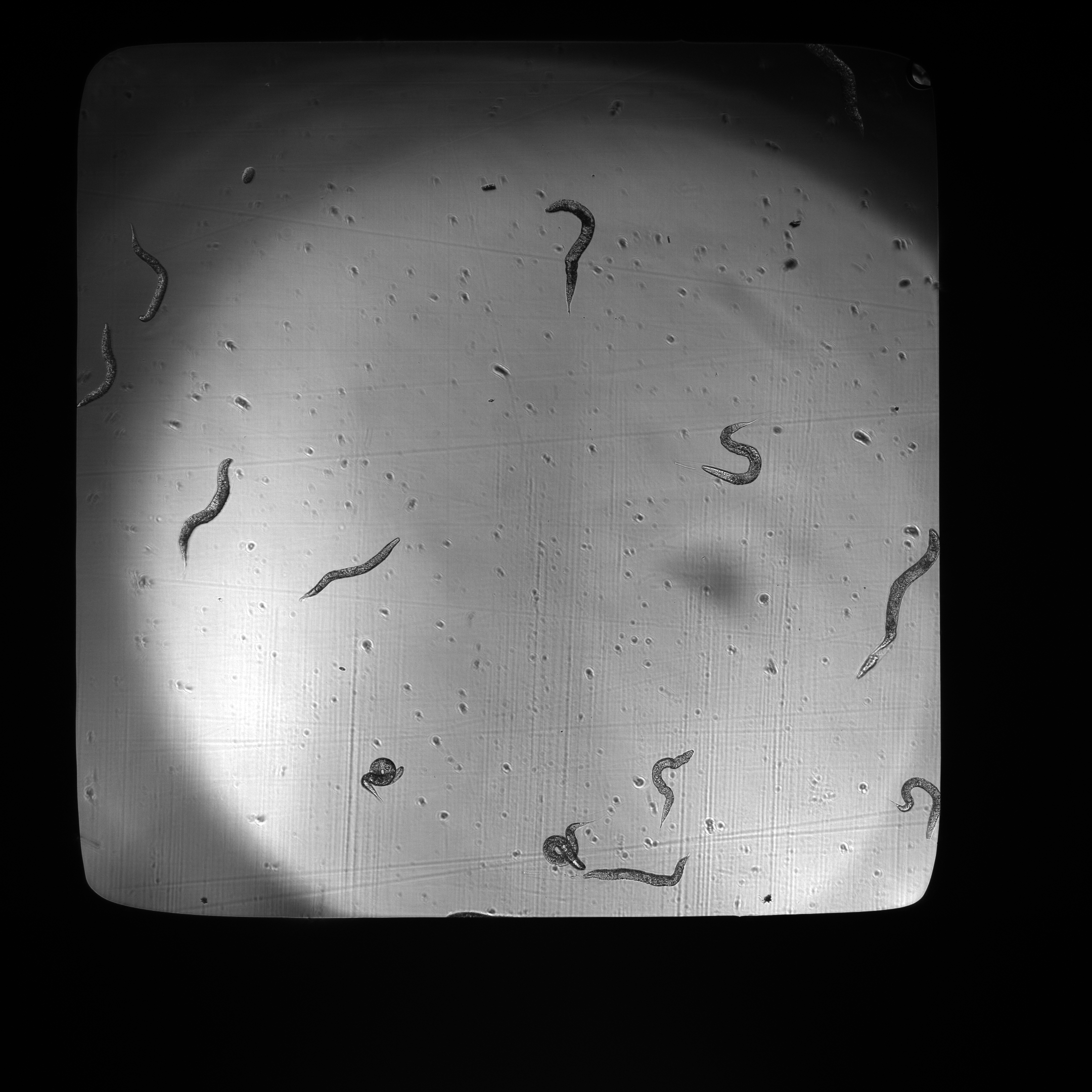

Supplement: Figure 4—source data 2. [file elife-76465-fig4-data2.zip › Figure 4C_source_data/Bort.tif]

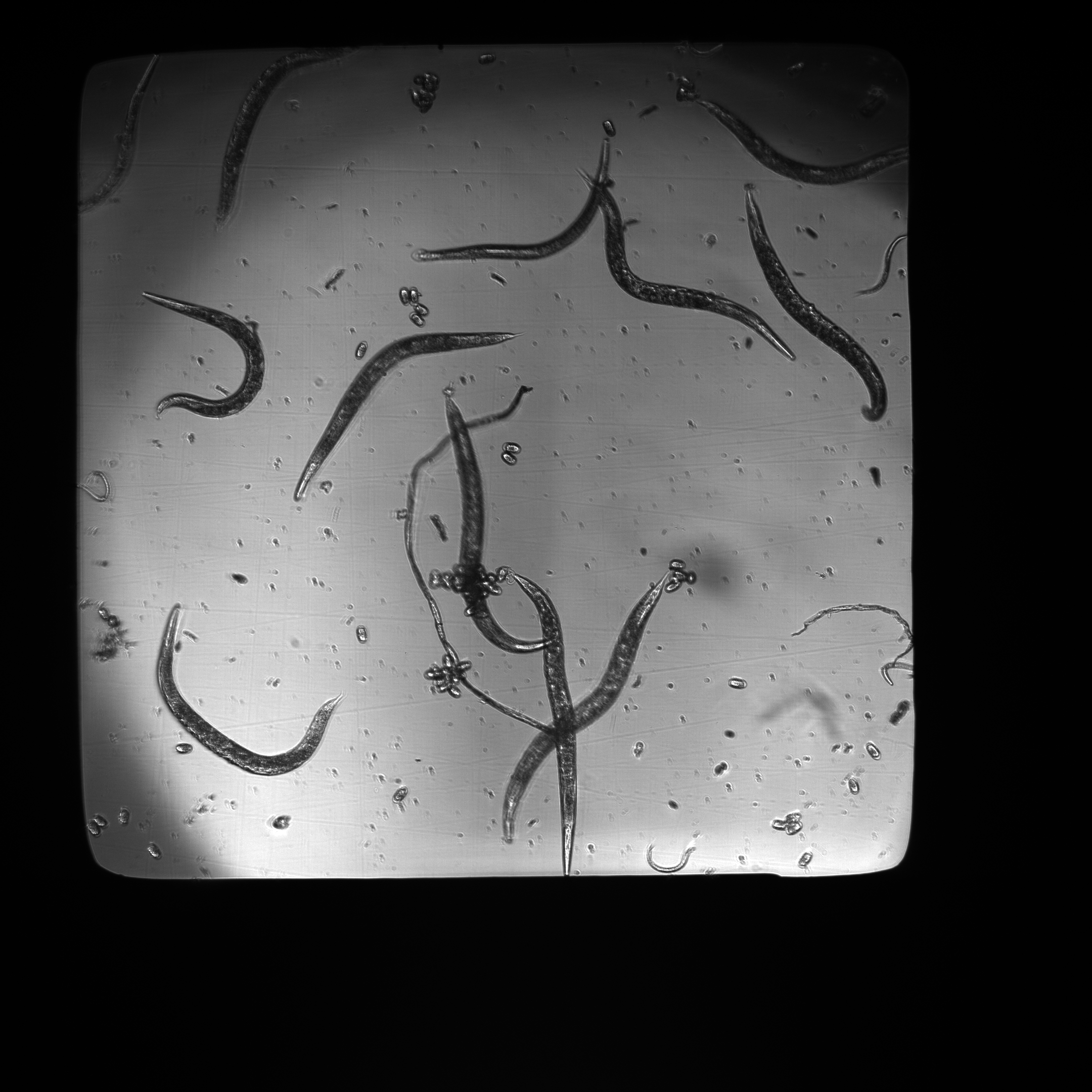

Supplement: Figure 4—source data 2. [file elife-76465-fig4-data2.zip › Figure 4C_source_data/DMSO.tif]

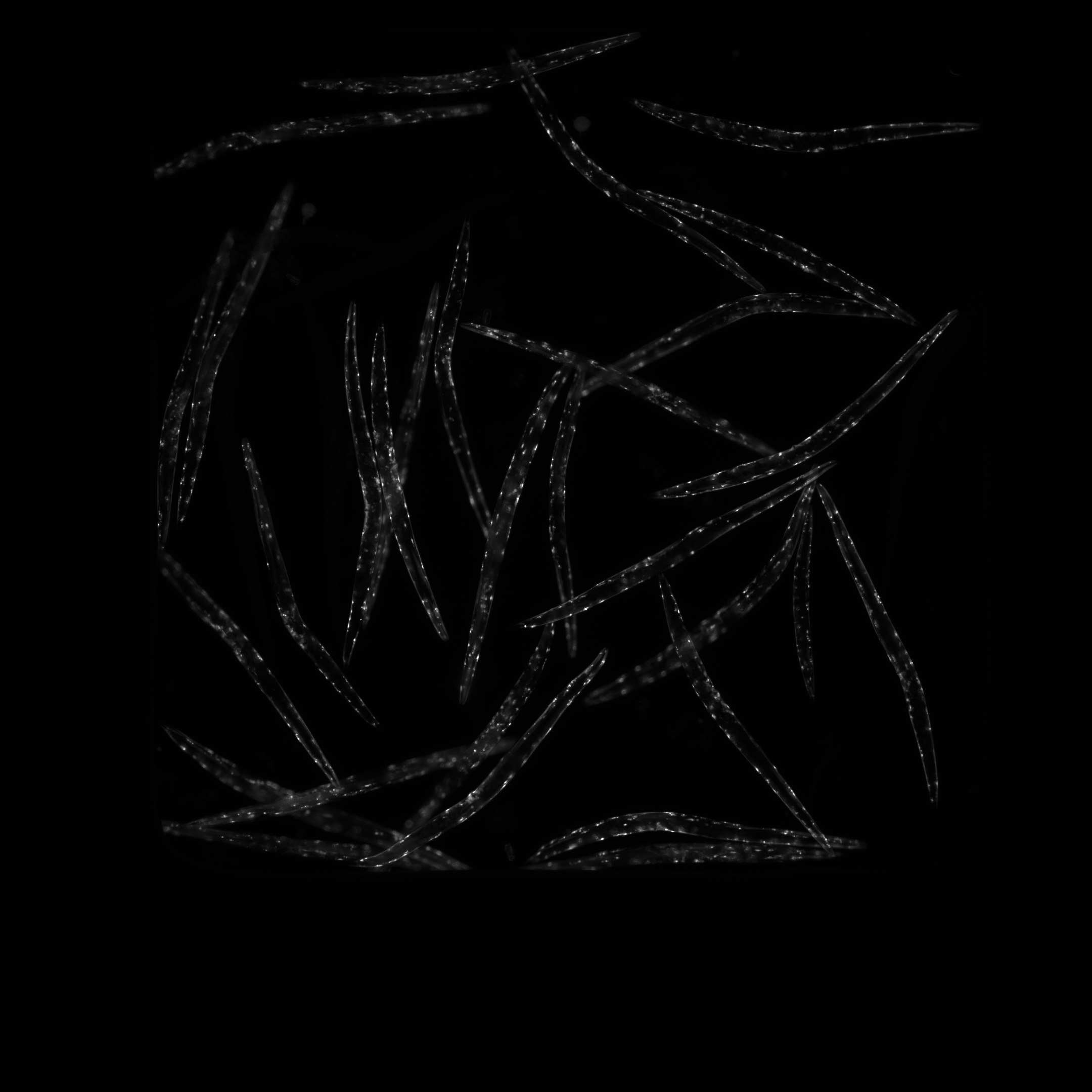

Supplement: Figure 4—source data 4. [file elife-76465-fig4-data4.zip › Figure 4E_source_data/Anis.tif]

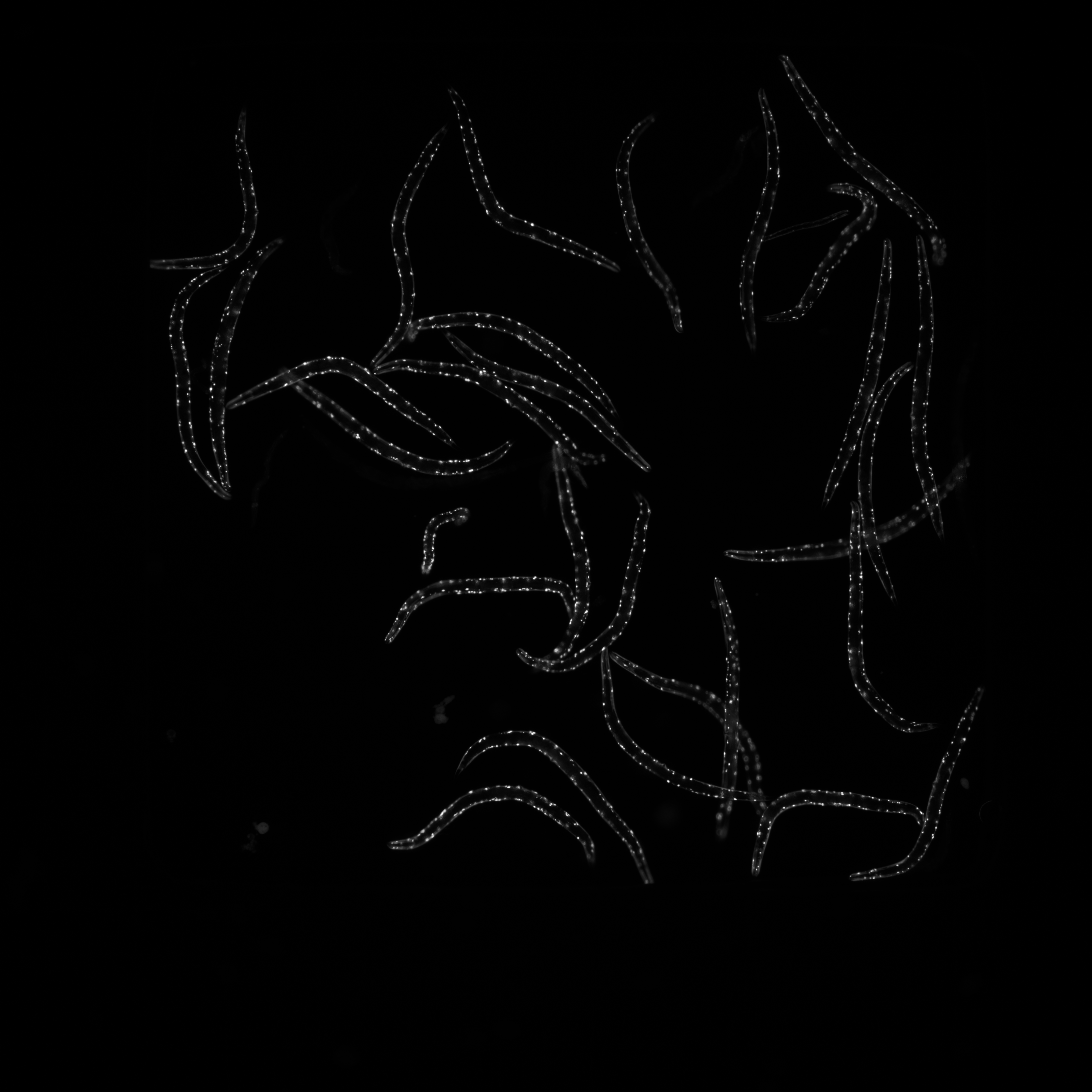

Supplement: Figure 4—source data 4. [file elife-76465-fig4-data4.zip › Figure 4E_source_data/Anisomycin_then_Bortezomib.tif]

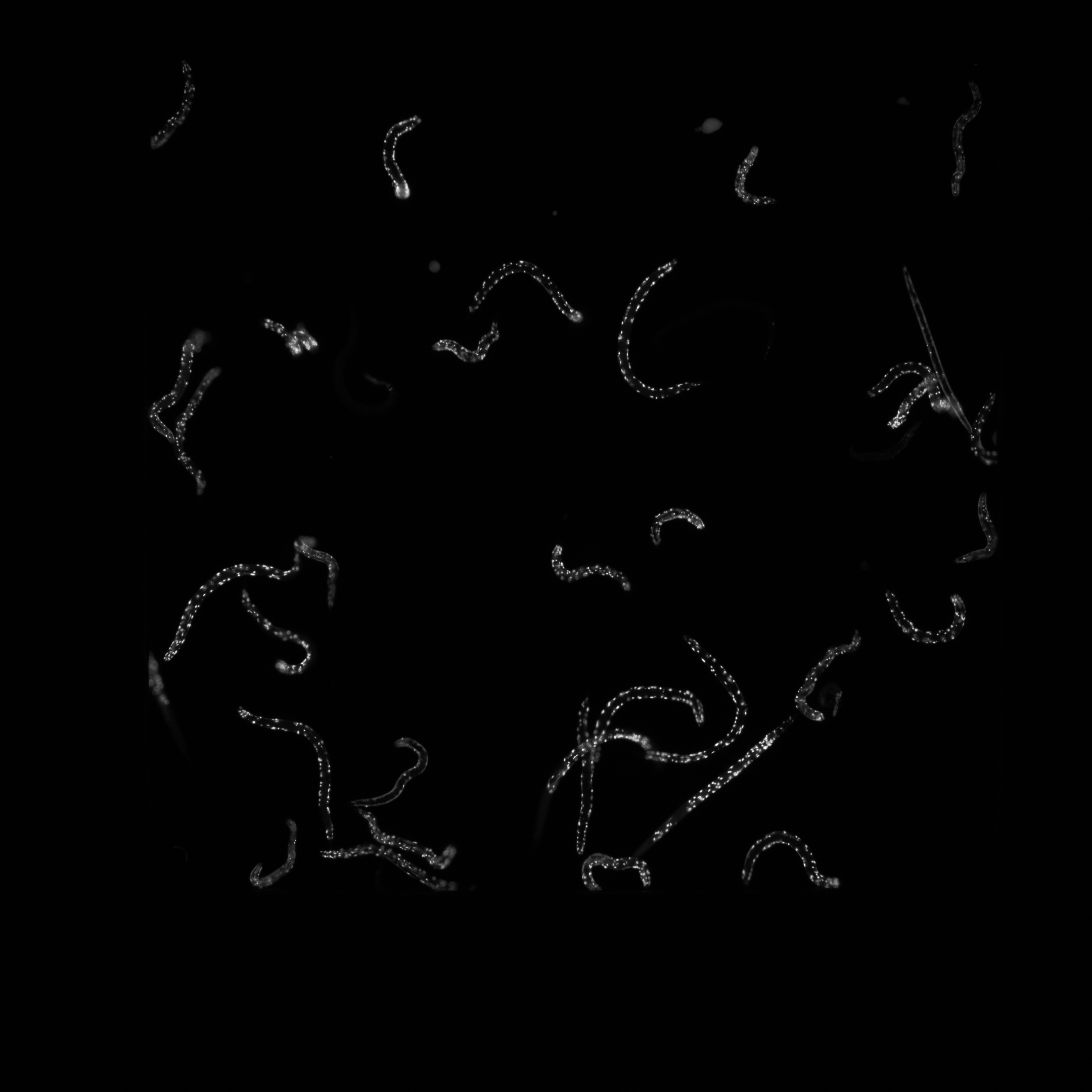

Supplement: Figure 4—source data 4. [file elife-76465-fig4-data4.zip › Figure 4E_source_data/Bort.tif]

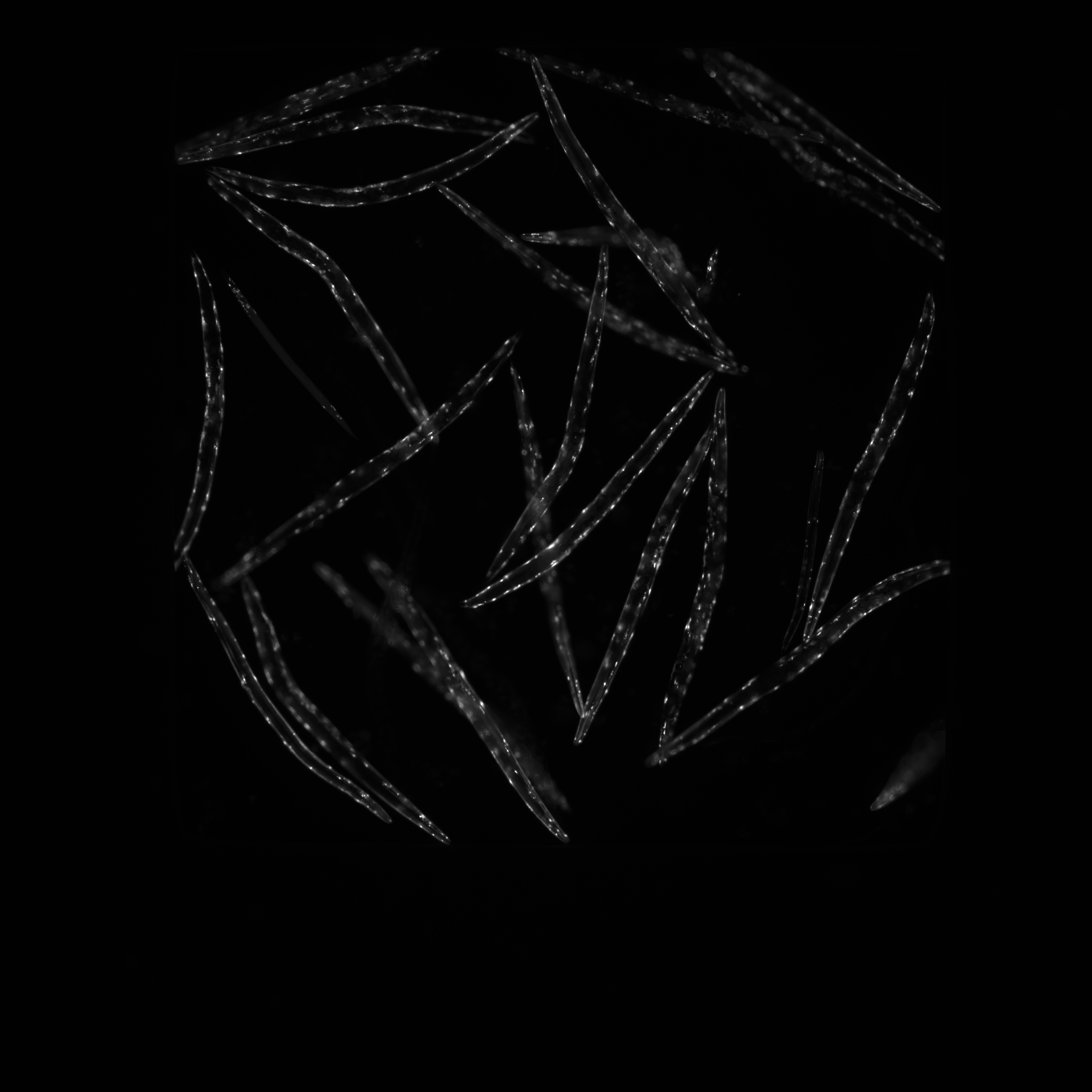

Supplement: Figure 4—source data 4. [file elife-76465-fig4-data4.zip › Figure 4E_source_data/DMSO.tif]

Figure 5D - source data

Repeat 1 Original picture

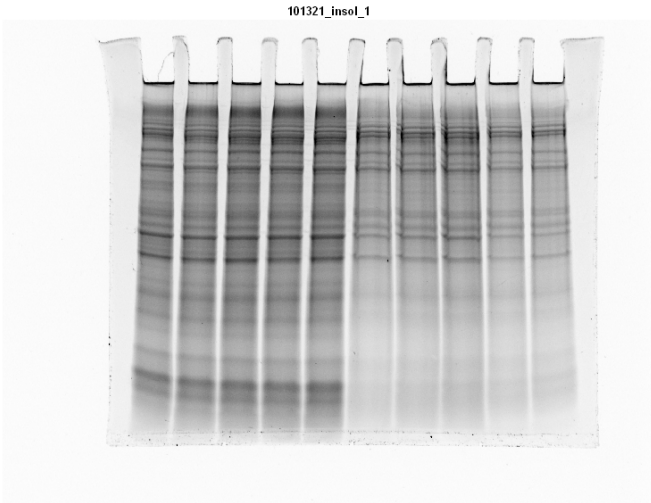

Labelled picture

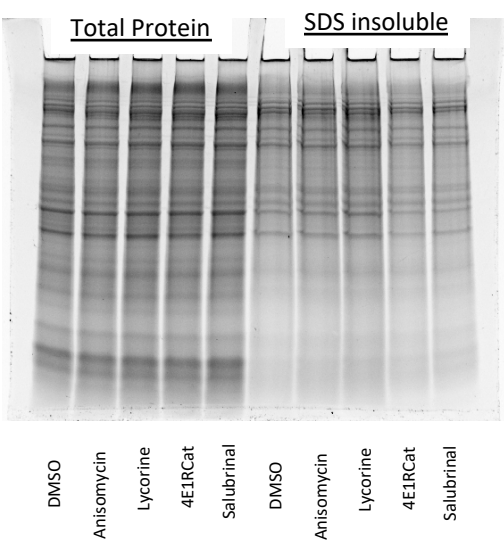

Repeat 2 Original picture

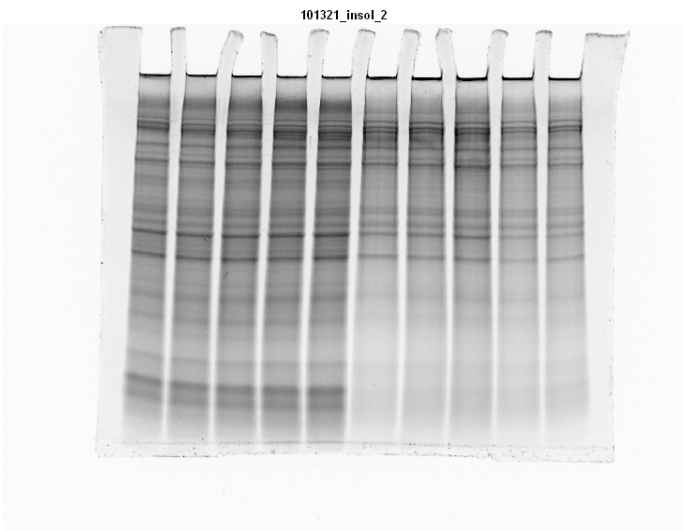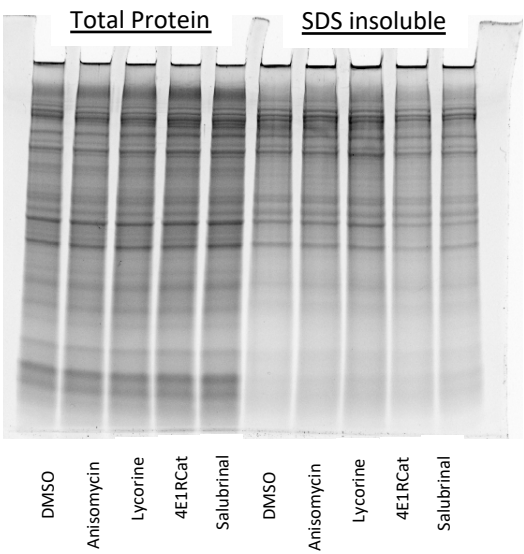

Supplement: Figure 5—source data 3. [file elife-76465-fig5-data3.zip › Figure 5D_source_data/Figure 5D-source data1.pdf]

Rep 1

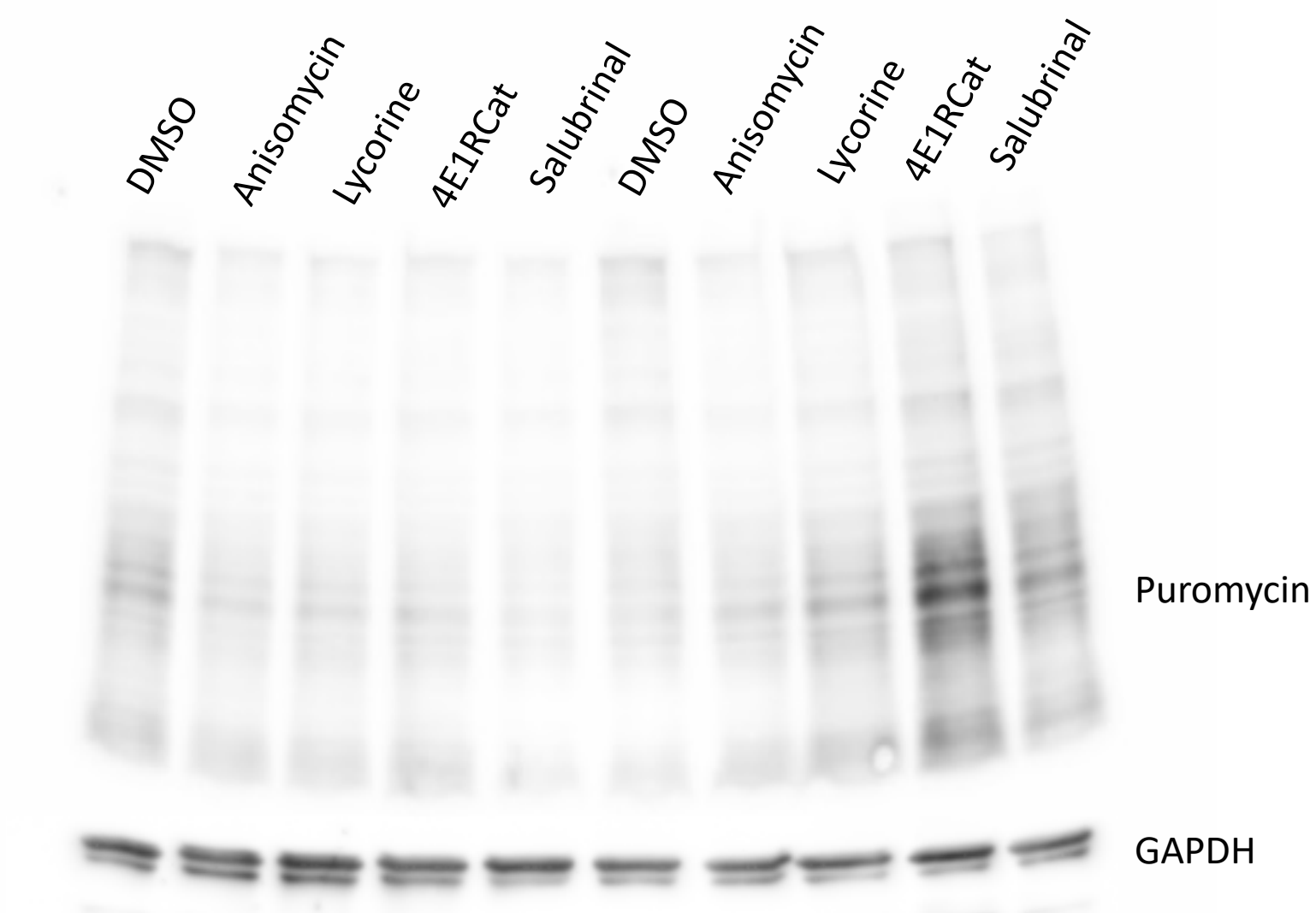

Rep 1

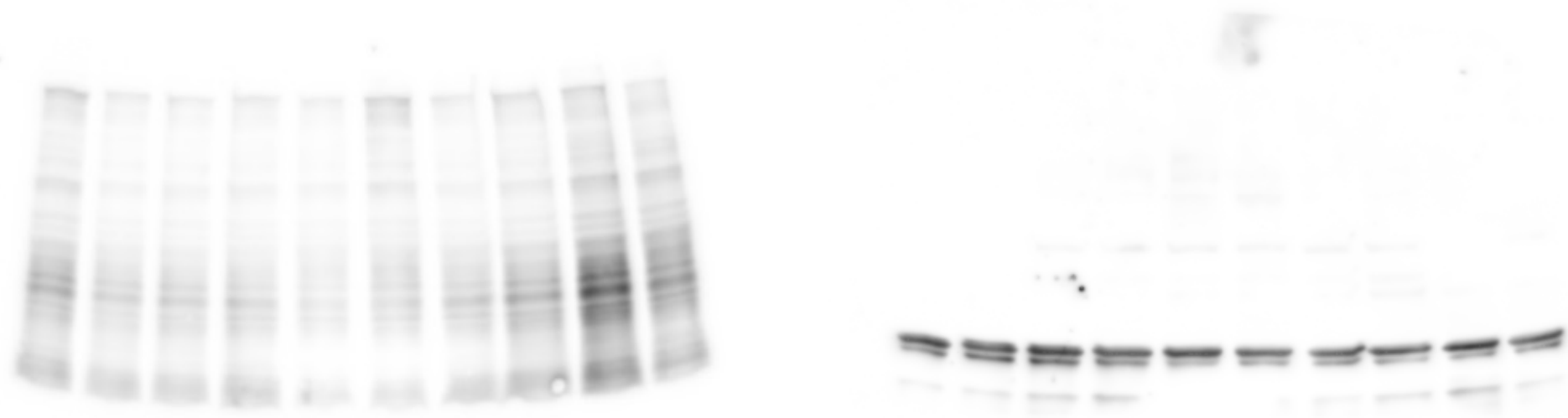

Rep 2

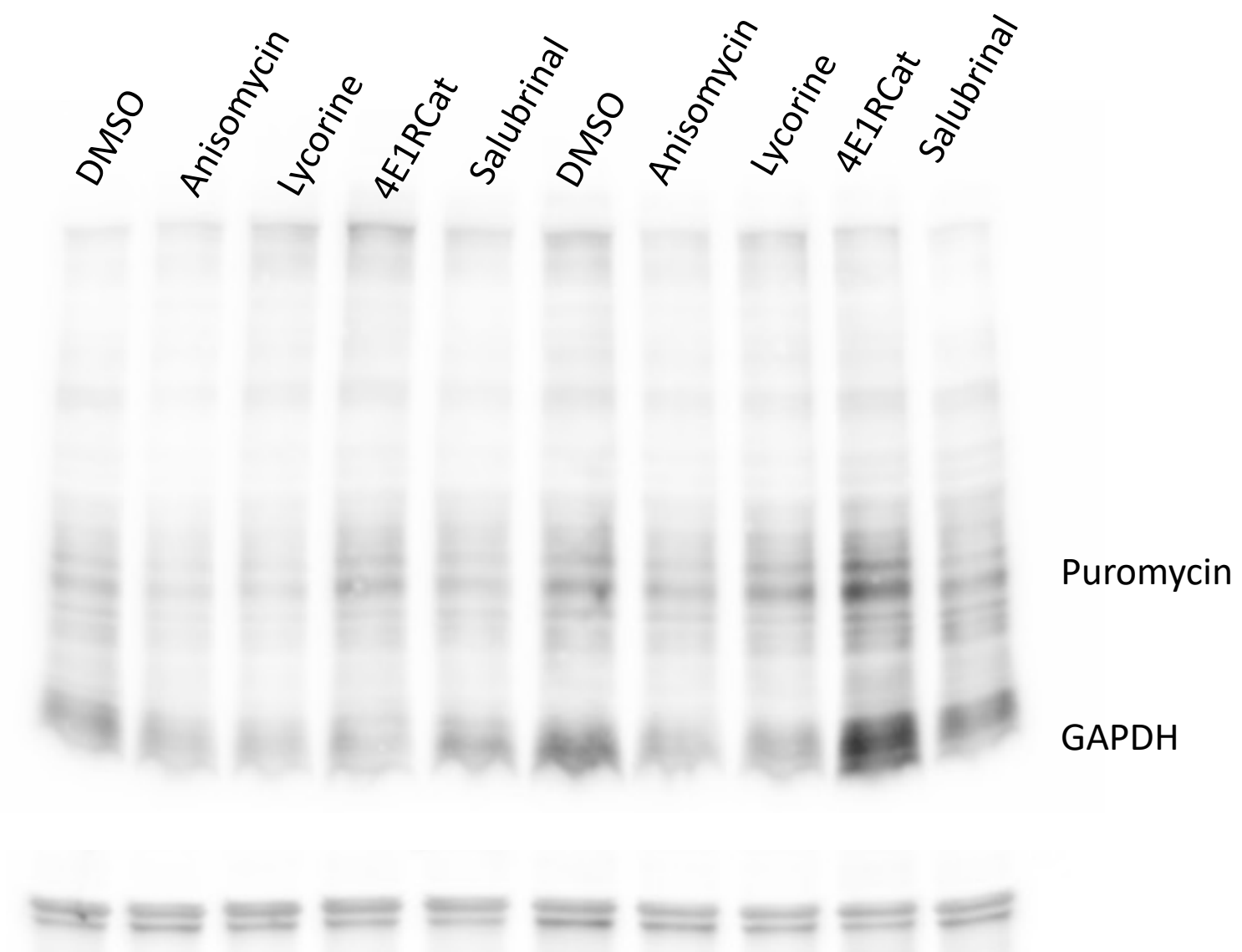

Rep 2

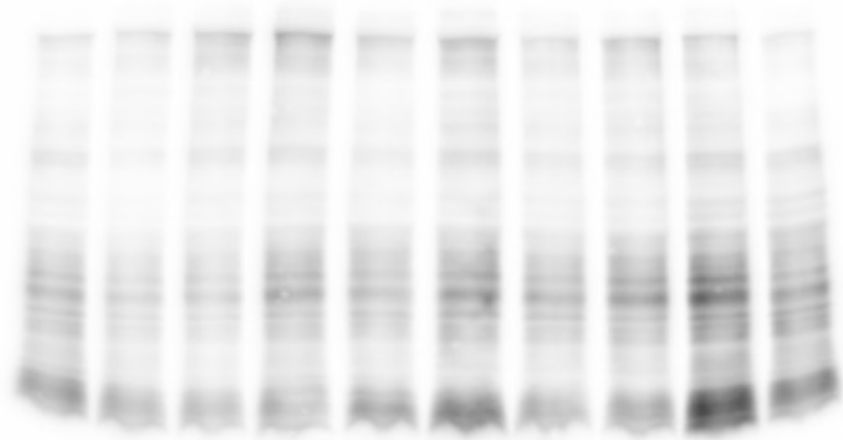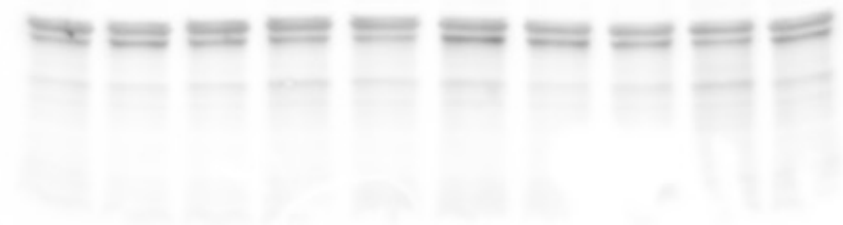

Rep 3

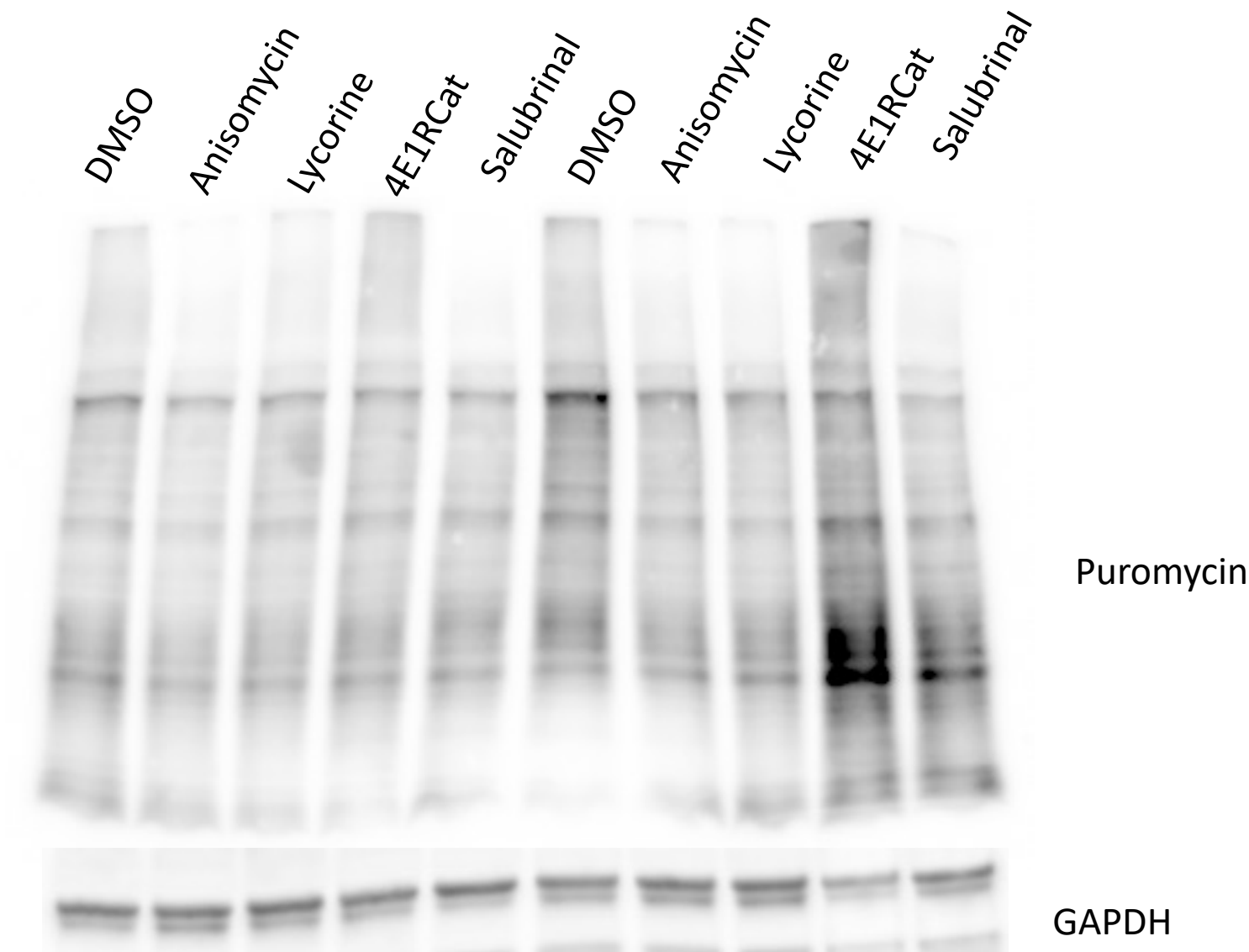

Rep 3

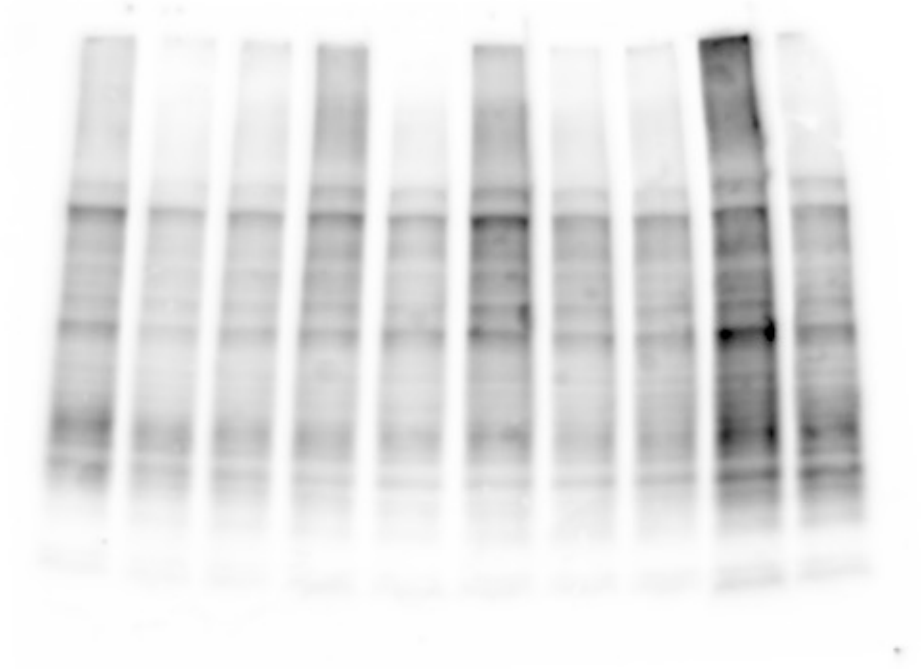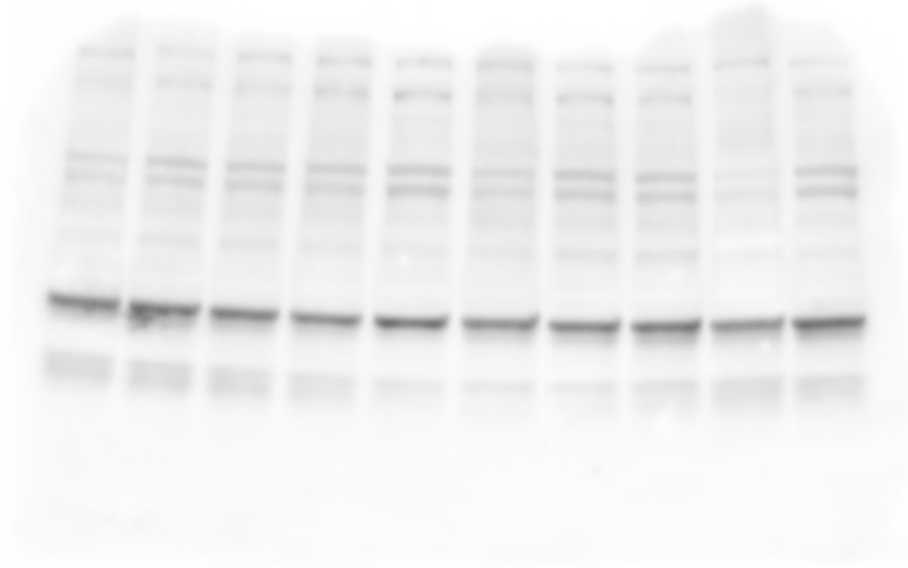

Rep 3

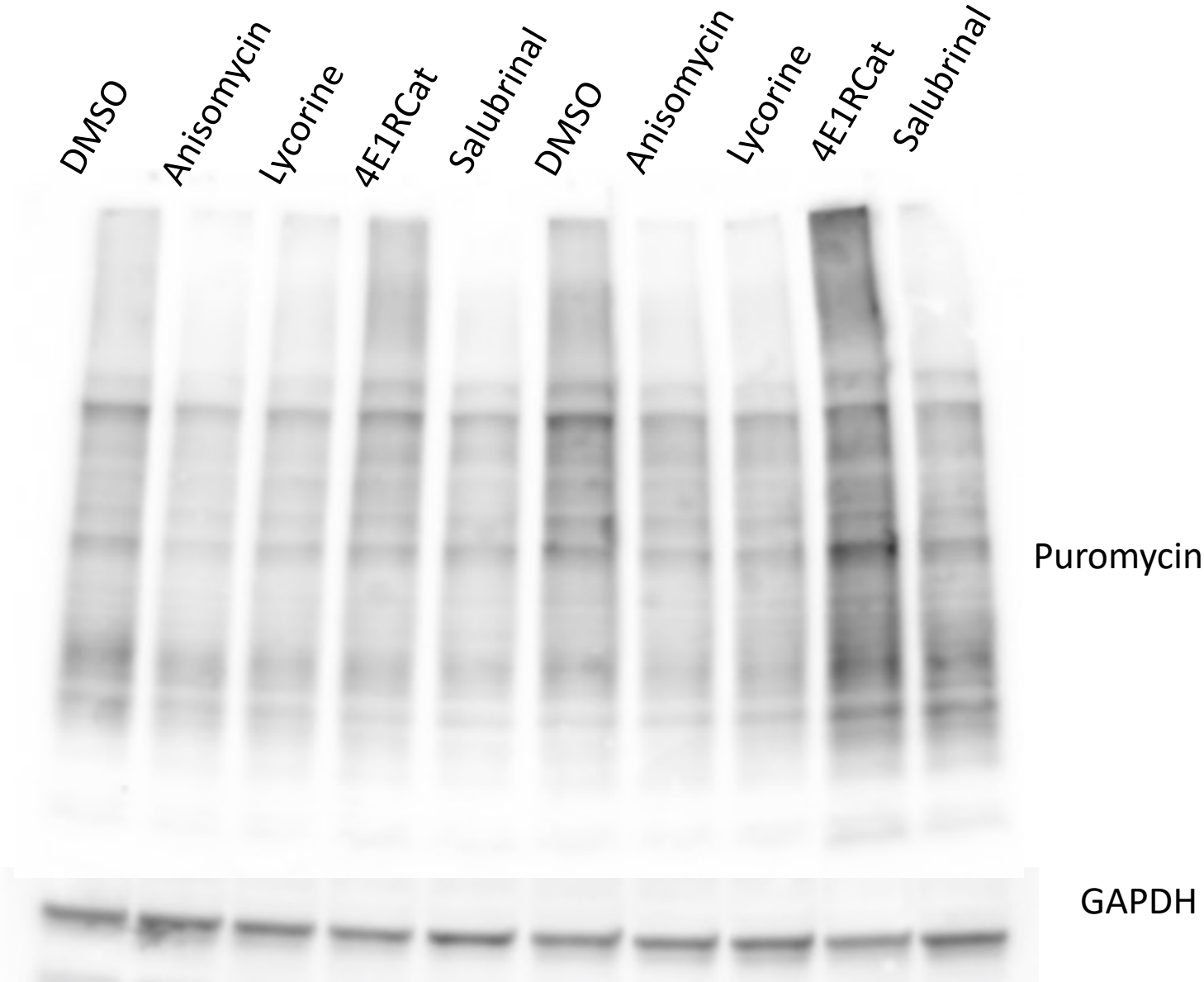

Rep 4

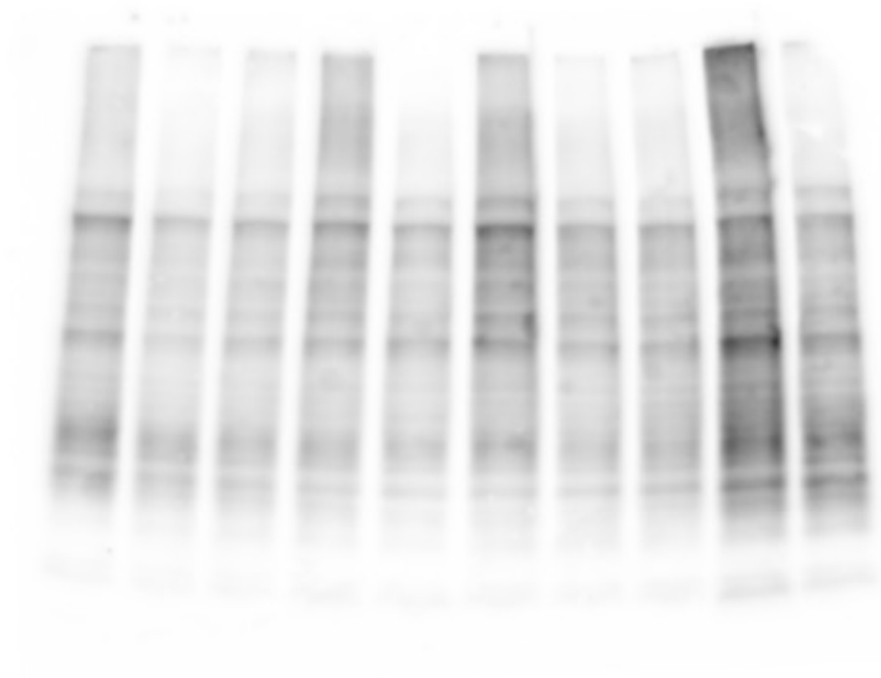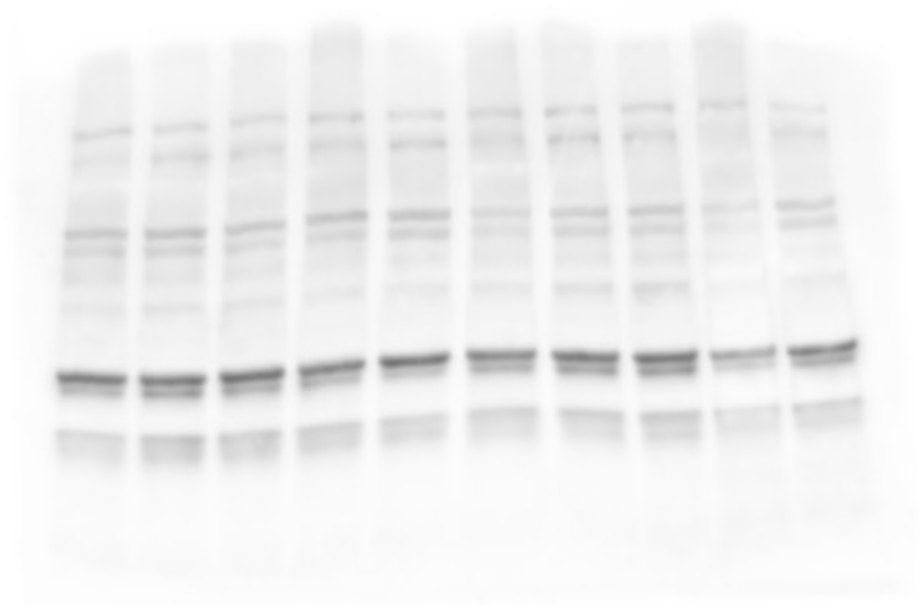

Supplement: Figure 6—source data 1. [file elife-76465-fig6-data1.zip › Figure 6A_source_data/Blots.pdf]
