# Supplementary material for: Proteostasis is differentially modulated by inhibition of translation initiation or elongation
Source: eLife. 2023 Oct 5;12:e76465. doi: 10.7554/eLife.76465 (PMC10581687; doi:10.7554/eLife.76465)

Figure 2D - source data

Repeat 1 Original picture

012621_SEQ_EXT_Run1_insoluble

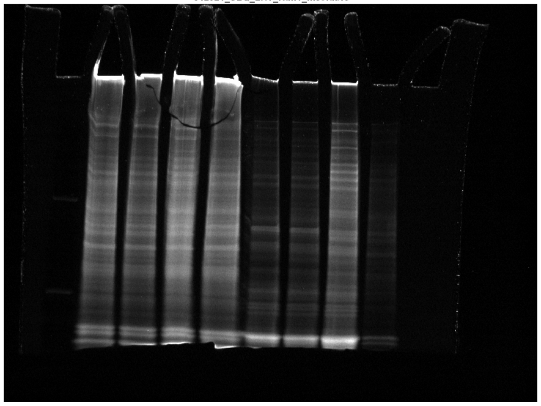

Labelled picture

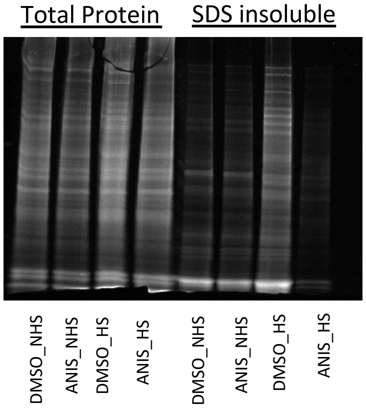

Repeat 2 Original picture

031121_SEQ_EXT_Run2_insoluble

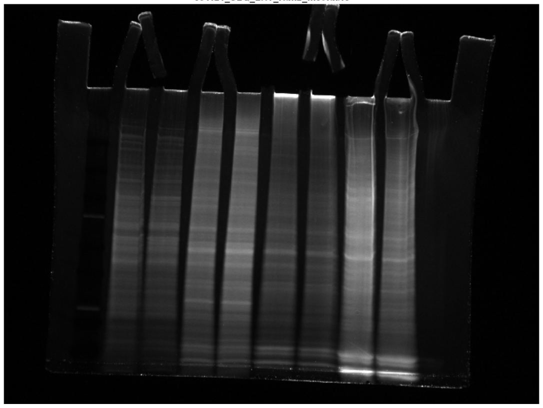

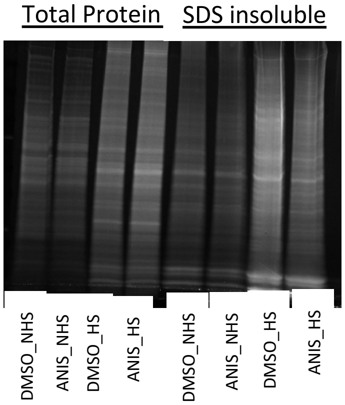

Repeat 3 Original picture

031121_SEQ_EXT_Run3_insoluble

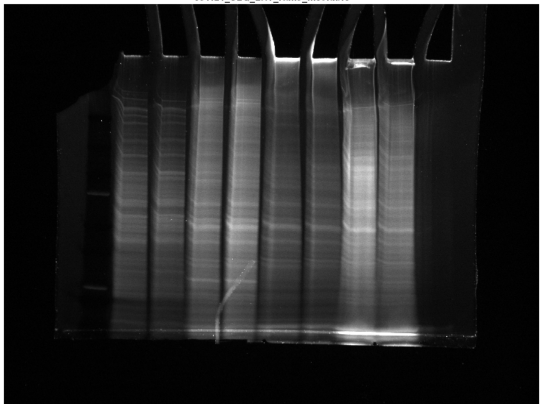

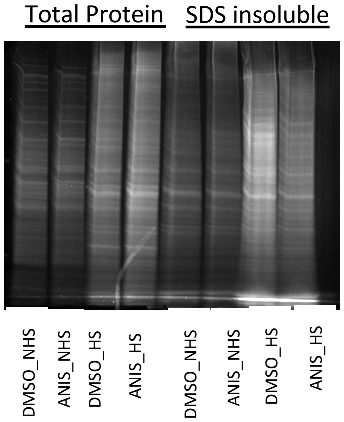

Repeat 4 Original picture

102120_SEQ_EXT_Run4_insoluble

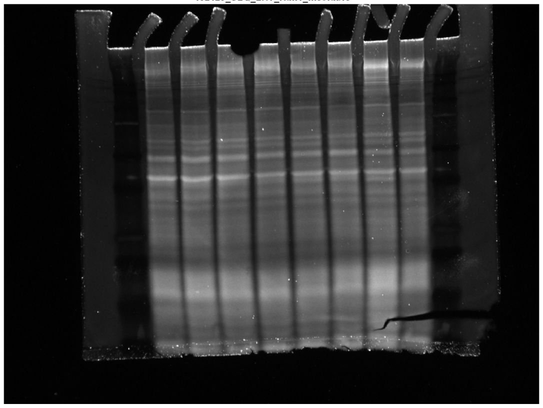

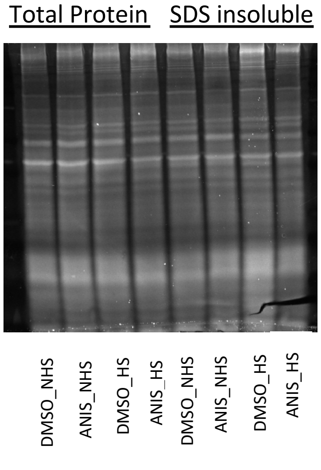

Supplement: Figure 2—source data 4. [file elife-76465-fig2-data4.zip › Figure 2E_source_data/Figure 2E-source data1.pdf]
