# Supplementary material for: Proteostasis is differentially modulated by inhibition of translation initiation or elongation
Source: eLife. 2023 Oct 5;12:e76465. doi: 10.7554/eLife.76465 (PMC10581687; doi:10.7554/eLife.76465)
Supplement: Figure 5—source data 3. [file elife-76465-fig5-data3.zip › Figure 5D_source_data/Figure 5D-source data2pptx.pptx]

## Slide 1
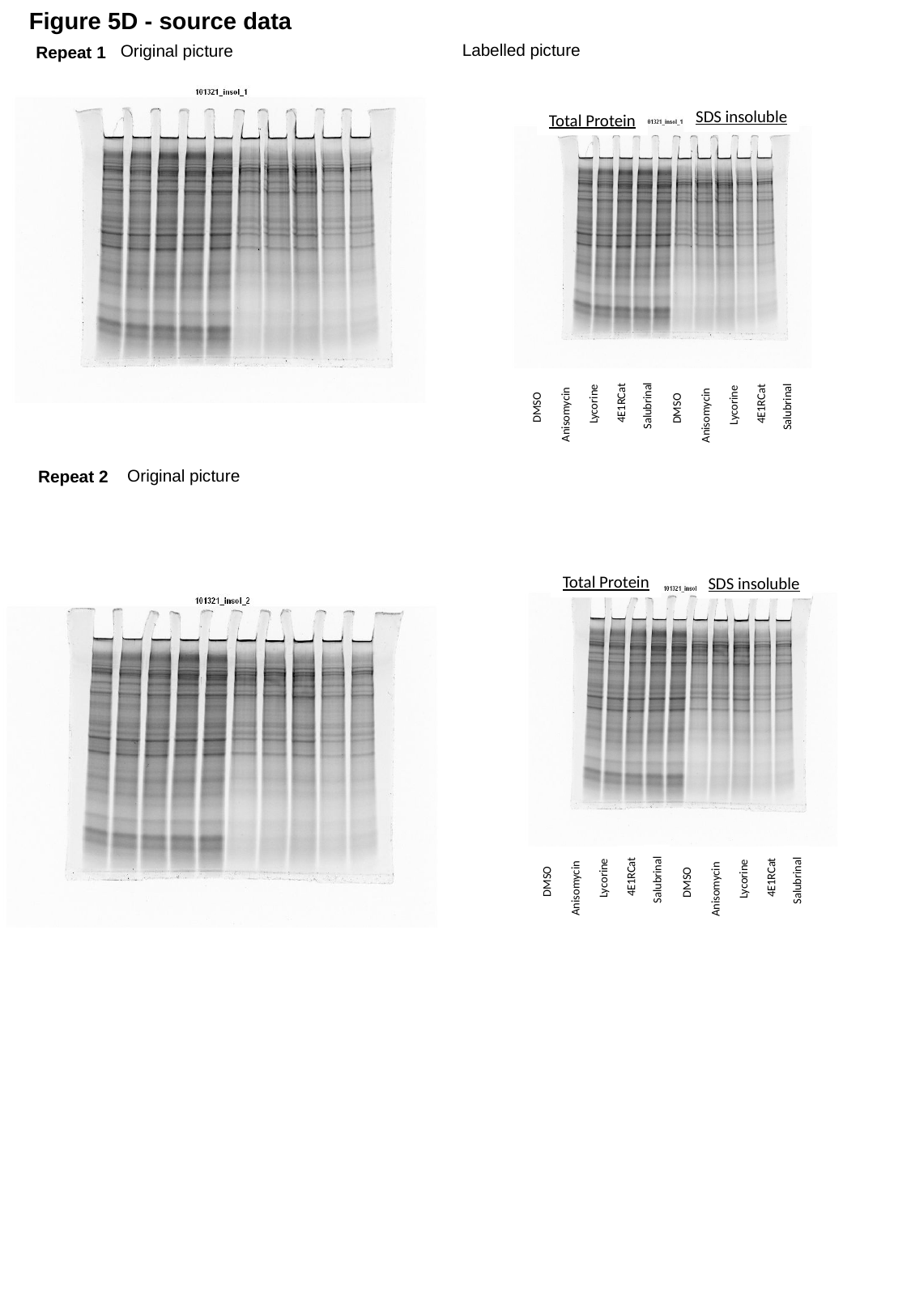

Figure 5D - source data
Labelled picture
Original picture
Repeat 1
SDS insoluble
Total Protein
4E1RCat
4E1RCat
Lycorine
Lycorine
Salubrinal
Salubrinal
DMSO
DMSO
Anisomycin
Anisomycin
Original picture
Repeat 2
Total Protein
SDS insoluble
4E1RCat
4E1RCat
Lycorine
Lycorine
Salubrinal
Salubrinal
DMSO
DMSO
Anisomycin
Anisomycin
