# Supplementary material for: Proteostasis is differentially modulated by inhibition of translation initiation or elongation
Source: eLife. 2023 Oct 5;12:e76465. doi: 10.7554/eLife.76465 (PMC10581687; doi:10.7554/eLife.76465)
Supplement: Figure 6—source data 1. [file elife-76465-fig6-data1.zip › Figure 6A_source_data/Blots.pptx]

## Slide 1
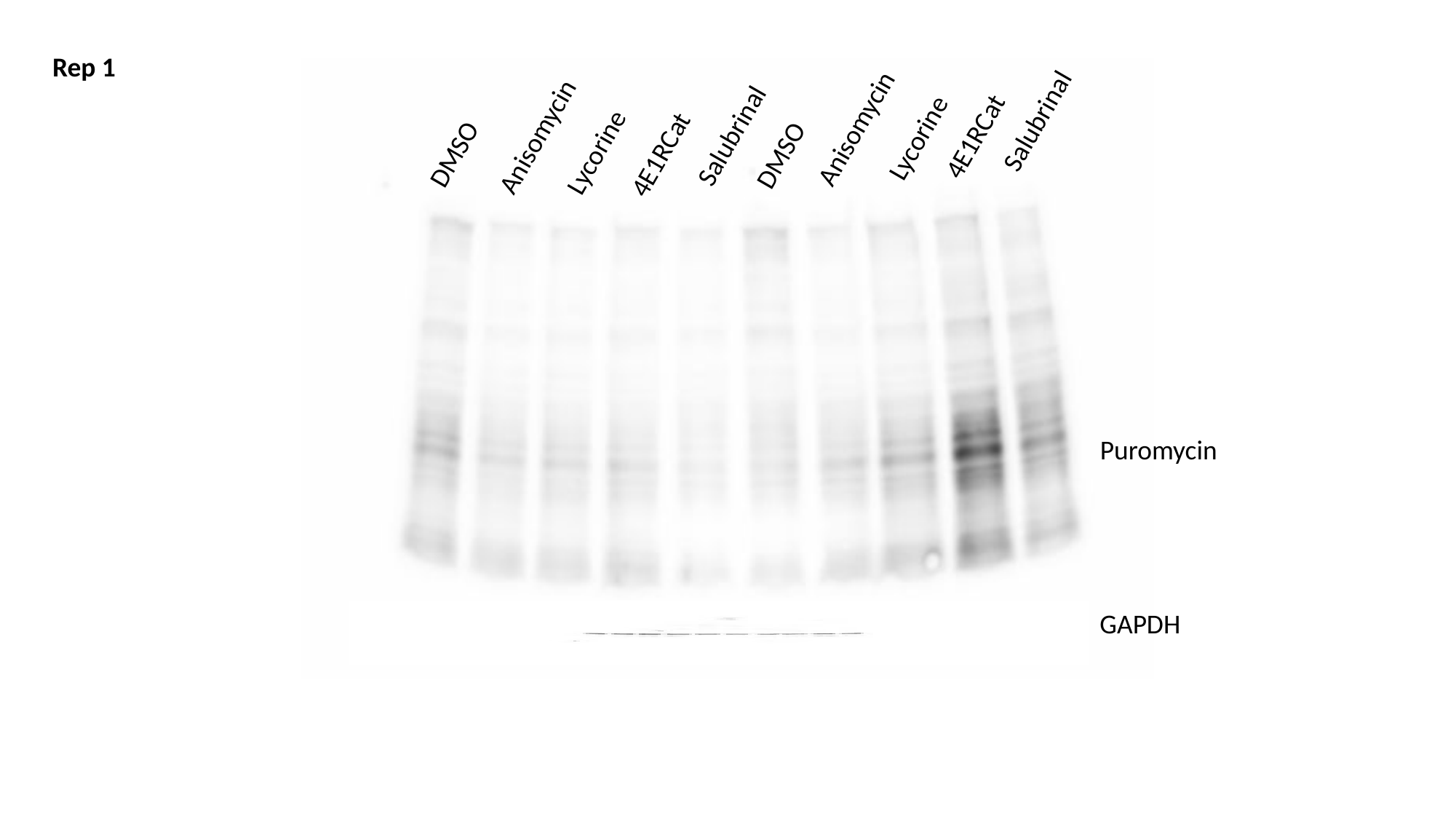

Rep 1
Salubrinal
Anisomycin
Salubrinal
Anisomycin
4E1RCat
Lycorine
Lycorine
DMSO
4E1RCat
DMSO
Puromycin
GAPDH

## Slide 2
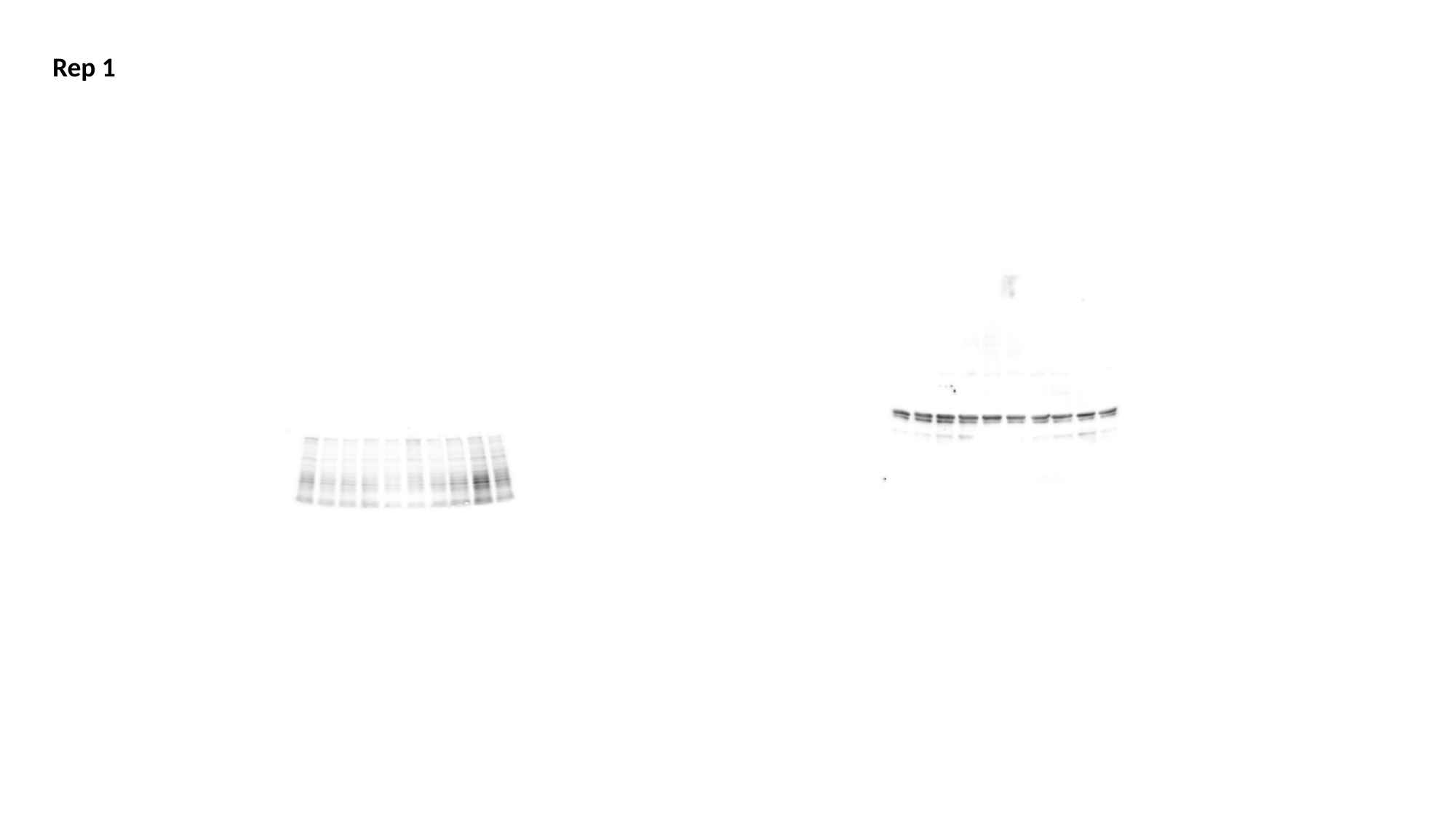

Rep 1

## Slide 3
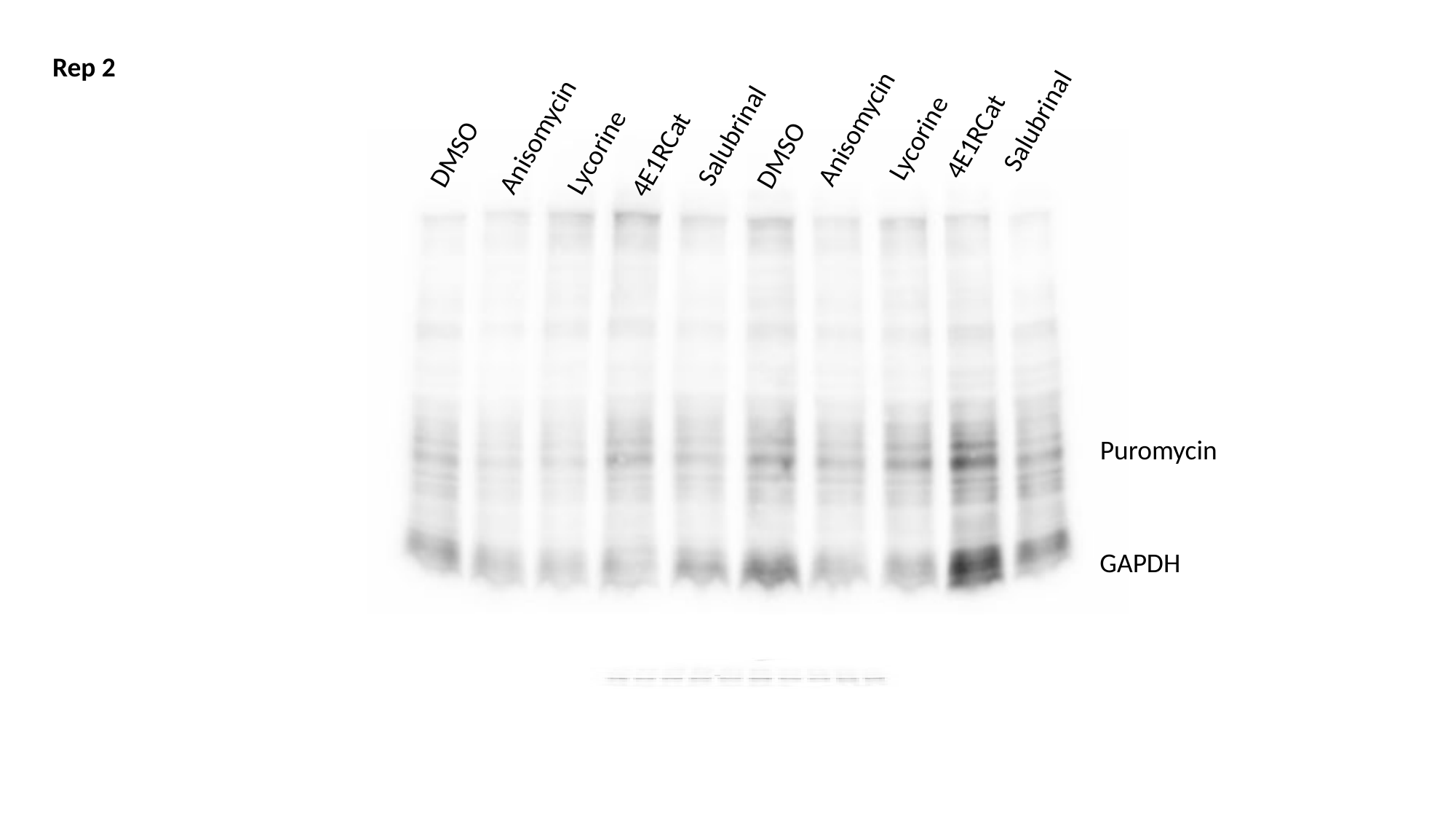

Rep 2
Salubrinal
Anisomycin
Salubrinal
Anisomycin
4E1RCat
Lycorine
Lycorine
DMSO
4E1RCat
DMSO
Puromycin
GAPDH

## Slide 4
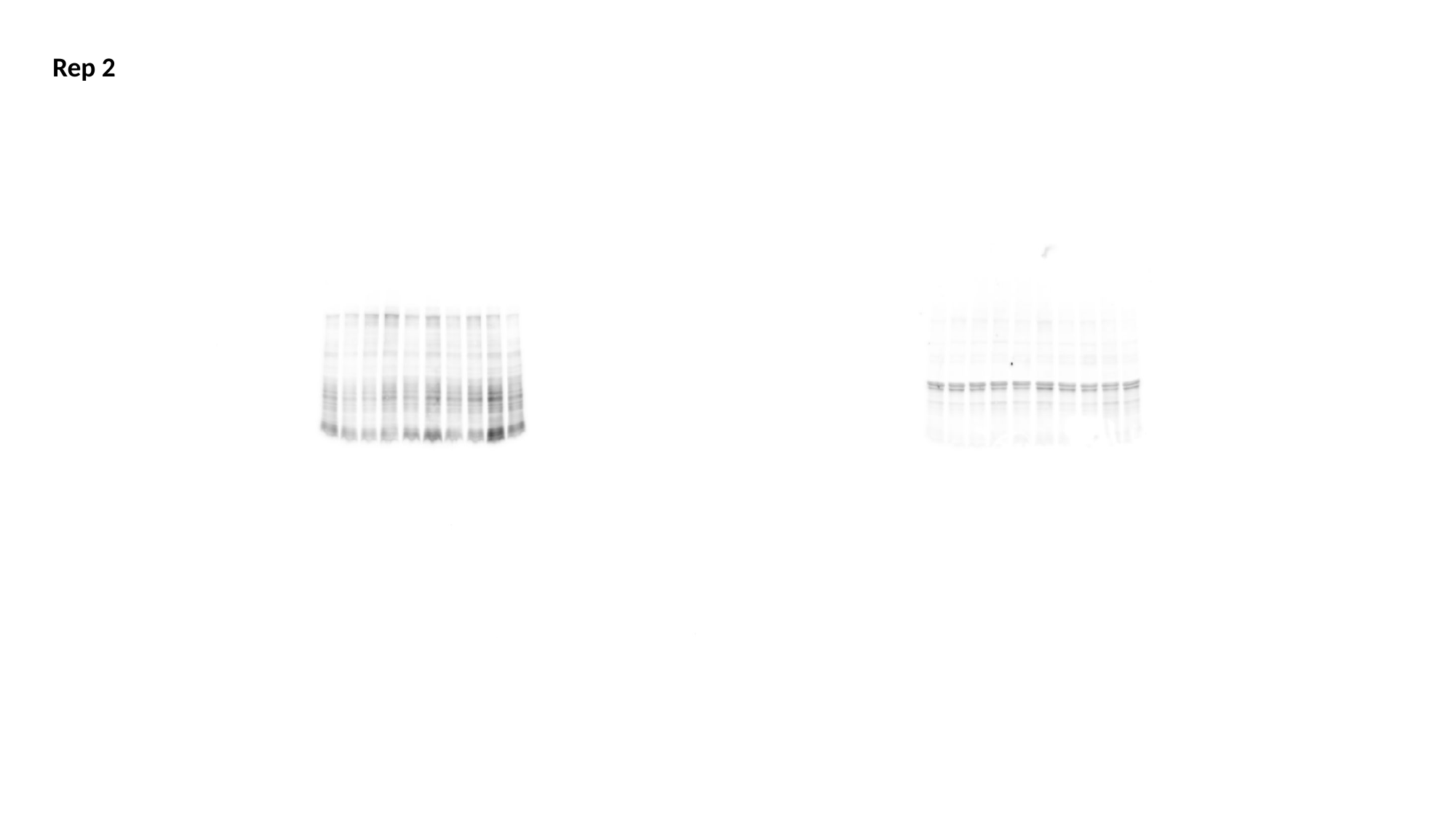

Rep 2

## Slide 5
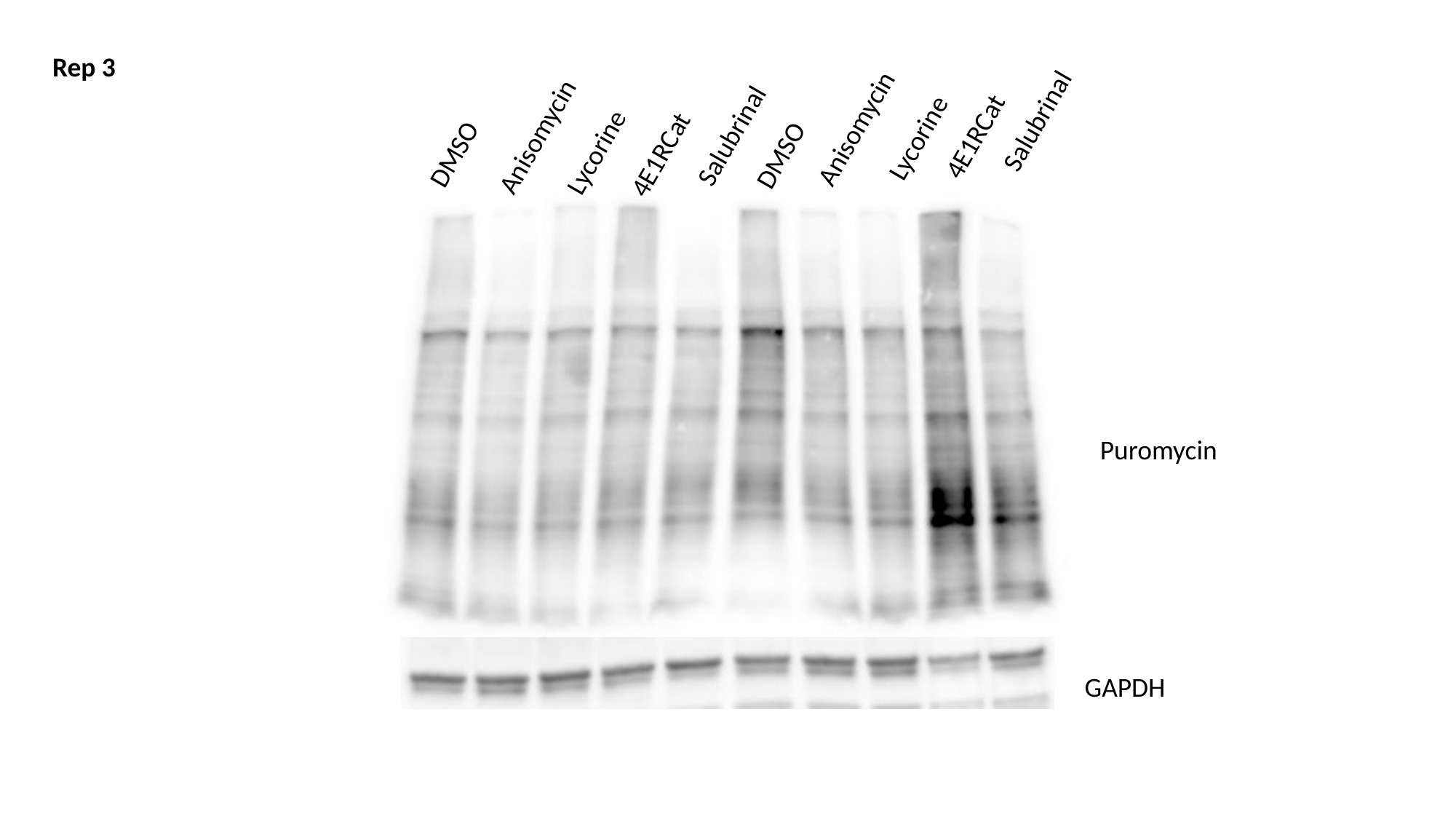

Rep 3
Salubrinal
Anisomycin
Salubrinal
Anisomycin
4E1RCat
Lycorine
Lycorine
DMSO
4E1RCat
DMSO
Puromycin
GAPDH

## Slide 6
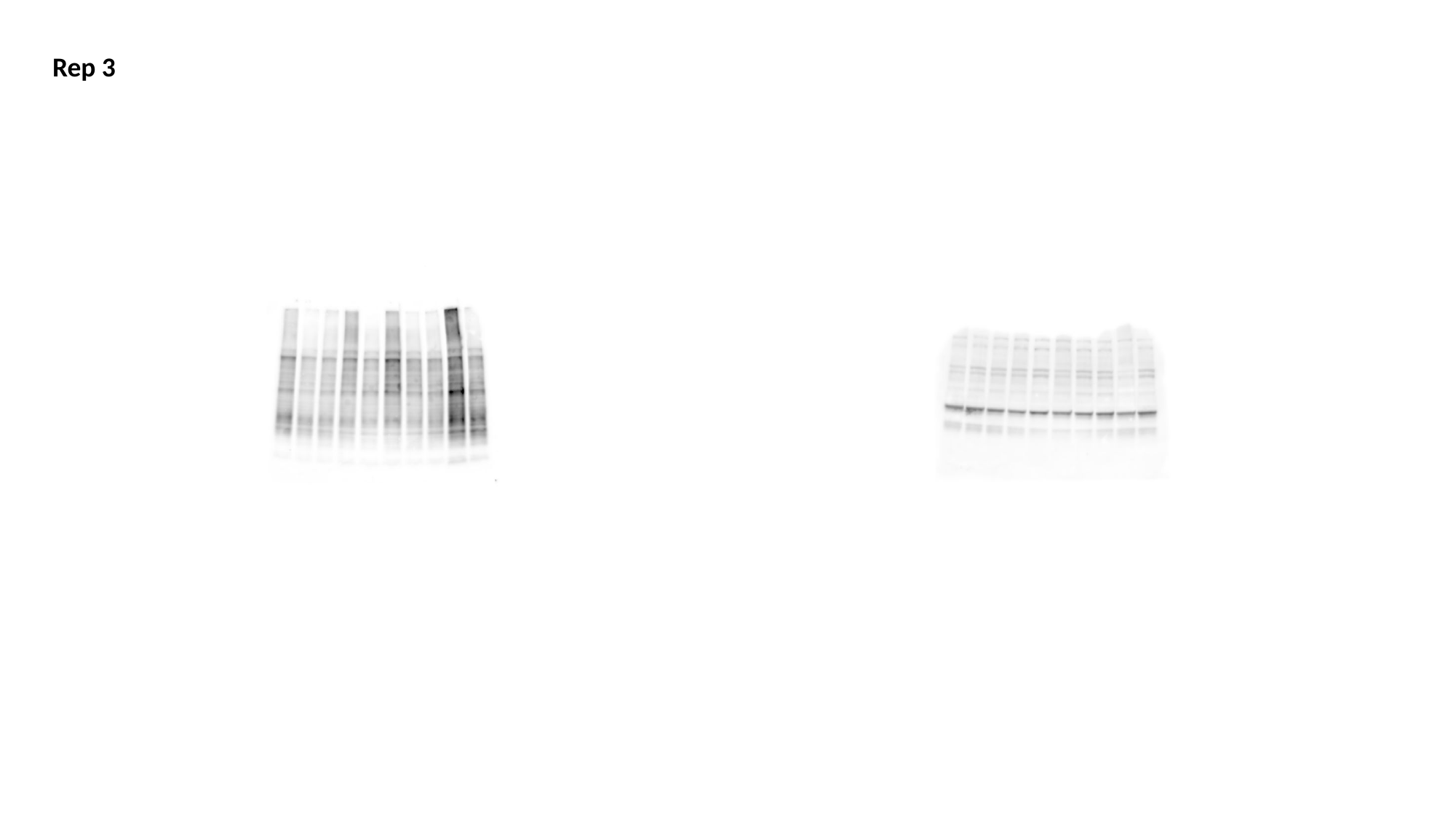

Rep 3

## Slide 7
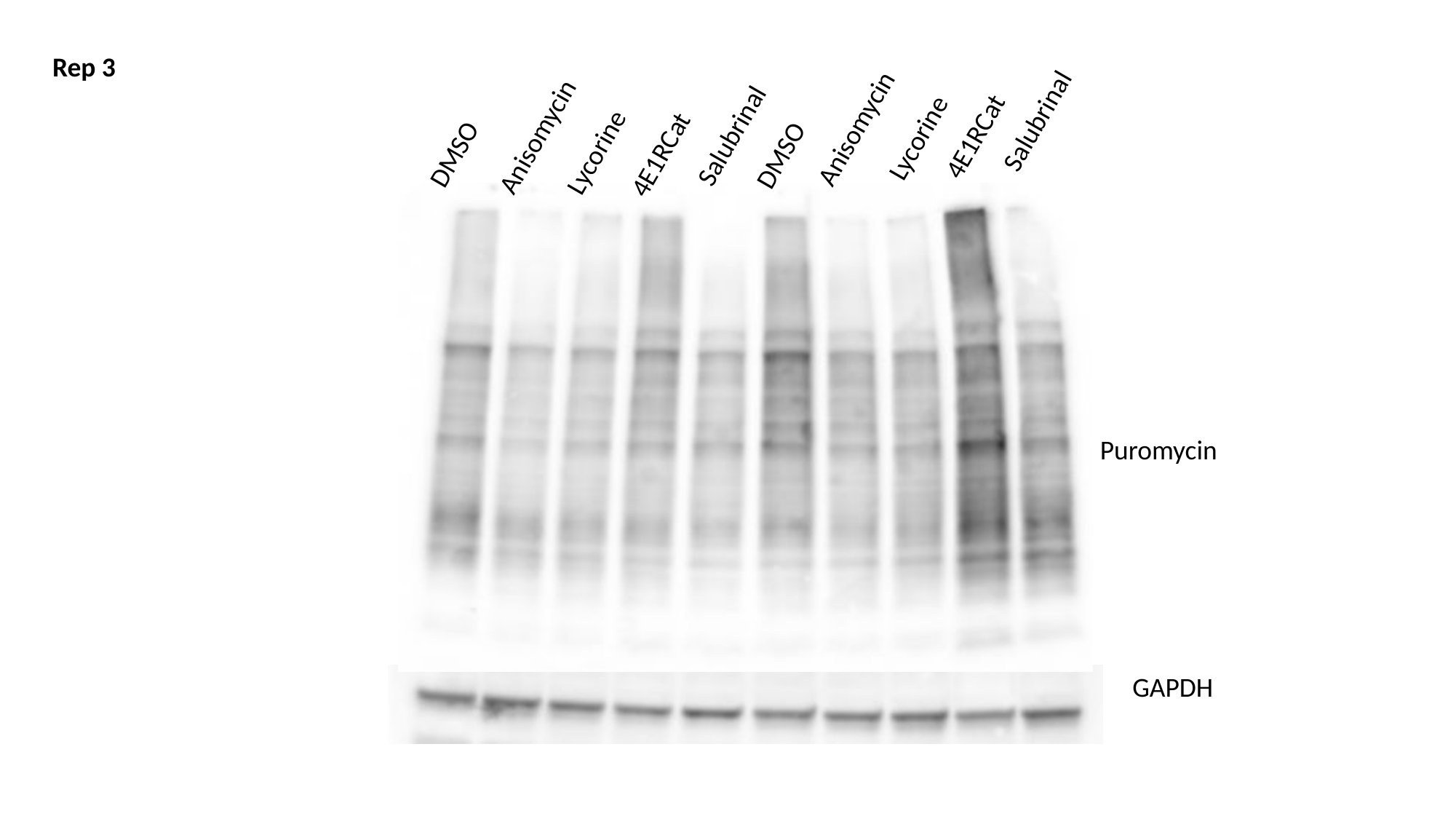

Rep 3
Salubrinal
Anisomycin
Salubrinal
Anisomycin
4E1RCat
Lycorine
Lycorine
DMSO
4E1RCat
DMSO
Puromycin
GAPDH

## Slide 8
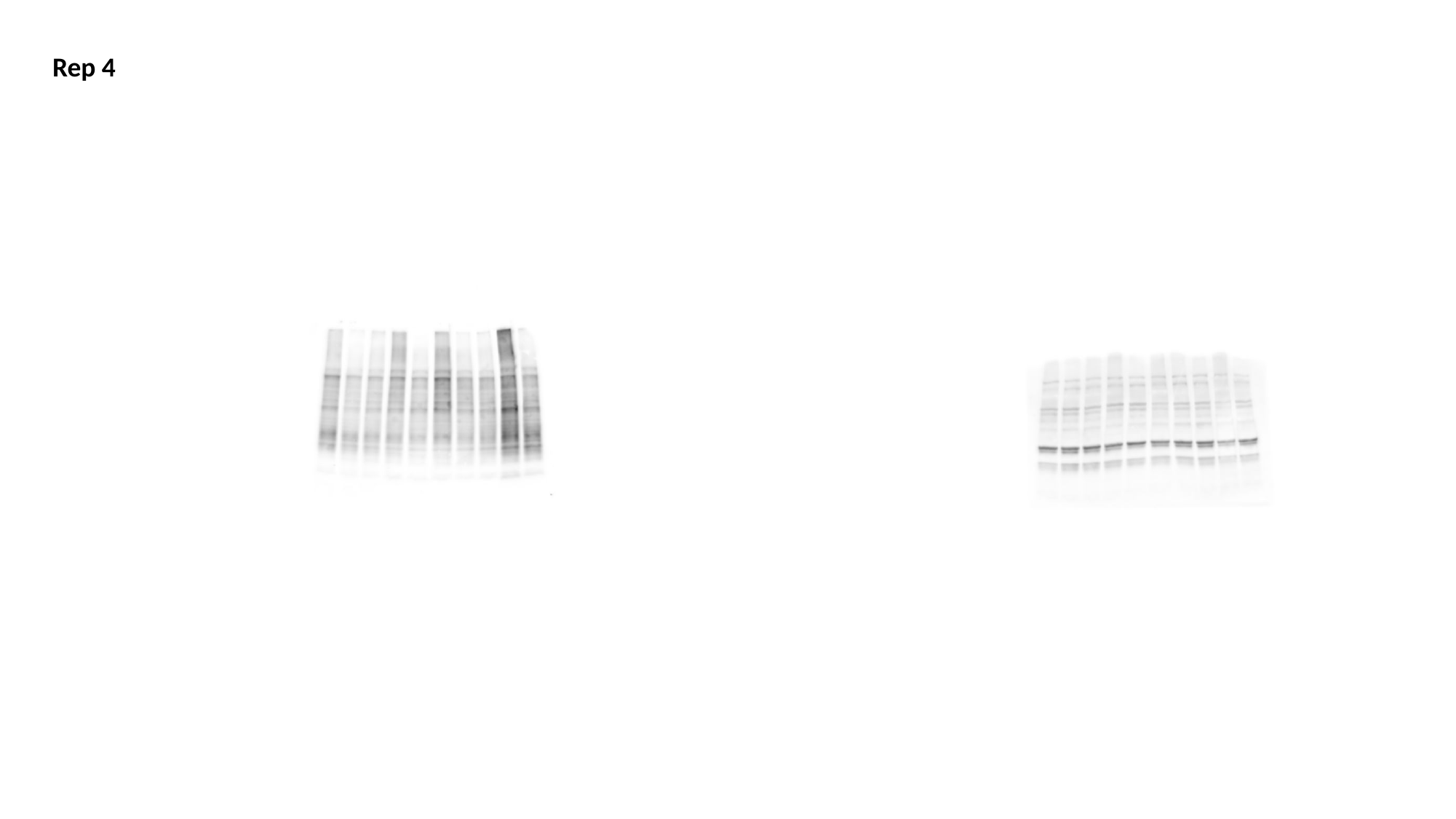

Rep 4
