# Supplementary material for: Proteostasis is differentially modulated by inhibition of translation initiation or elongation
Source: eLife. 2023 Oct 5;12:e76465. doi: 10.7554/eLife.76465 (PMC10581687; doi:10.7554/eLife.76465)
Supplement: Figure 6—source data 5. [file elife-76465-fig6-data5.zip › Figure 6E_source_data/pics.pdf]

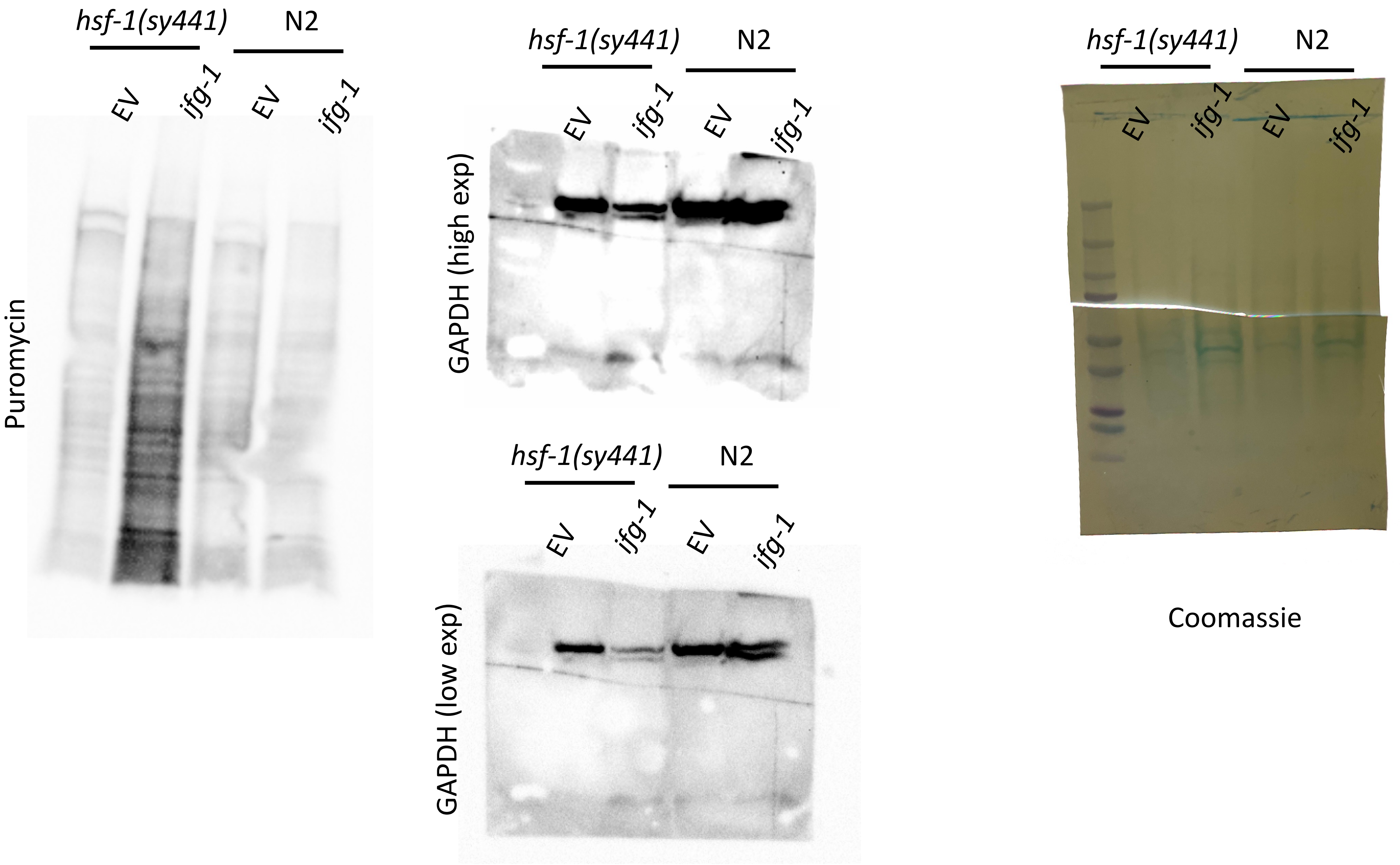
hsf-1(sy441)
N2
EV
ifg-1
EV
ifg-1
Puromycin
GAPDH (high exp)
GAPDH (low exp)
Coomassie

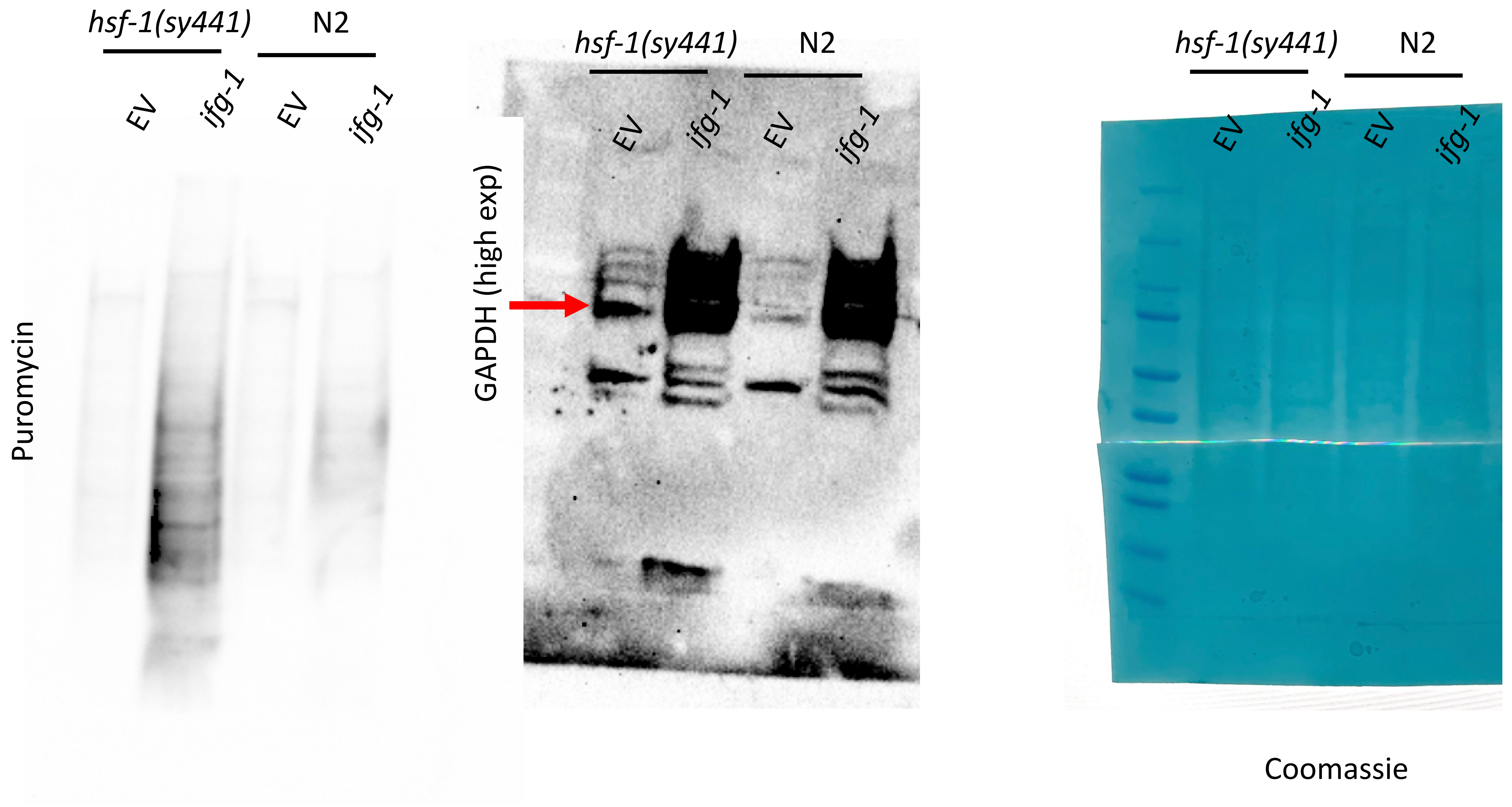
hsf-1(sy441)
N2
EV
ifg-1
EV
ifg-1
Puromycin
hsf-1(sy441)
N2
EV
ifg-1
EV
ifg-1
GAPDH (high exp)
hsf-1(sy441)
N2
EV
ifg-1
EV
ifg-1
Coomassie

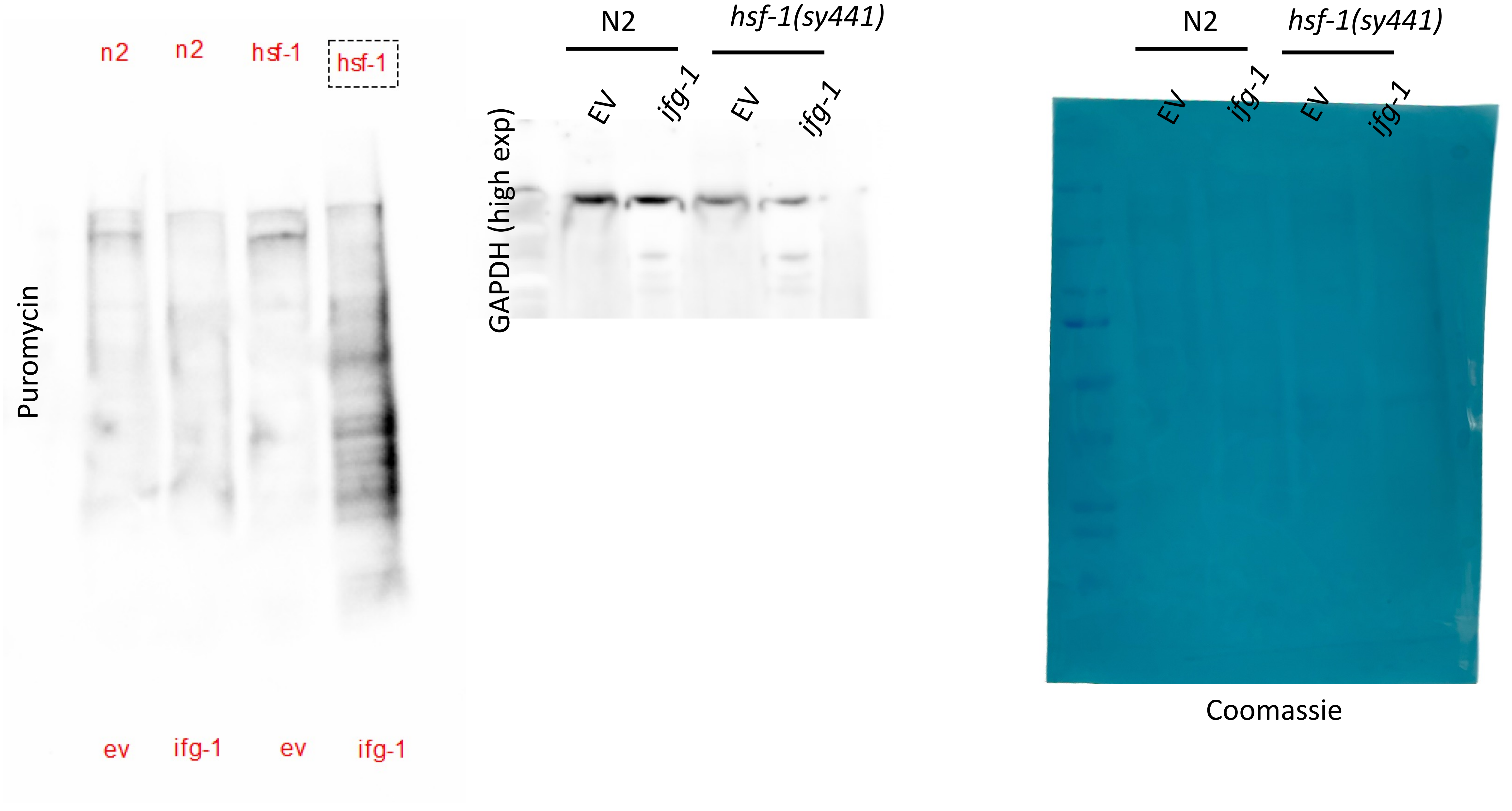
n2
n2
hsf-1
hsf-1
Puromycin
ev
ifg-1
ev
ifg-1
N2
hsf-1(sy441)
EV
ifg-1
EV
ifg-1
GAPDH (high exp)
N2
hsf-1(sy441)
EV
ifg-1
EV
ifg-1
Coomassie
