# Supplementary material for: Proteostasis is differentially modulated by inhibition of translation initiation or elongation
Source: eLife. 2023 Oct 5;12:e76465. doi: 10.7554/eLife.76465 (PMC10581687; doi:10.7554/eLife.76465)
Supplement: Figure 6—source data 5. [file elife-76465-fig6-data5.zip › Figure 6E_source_data/pics.pptx]

## Slide 1
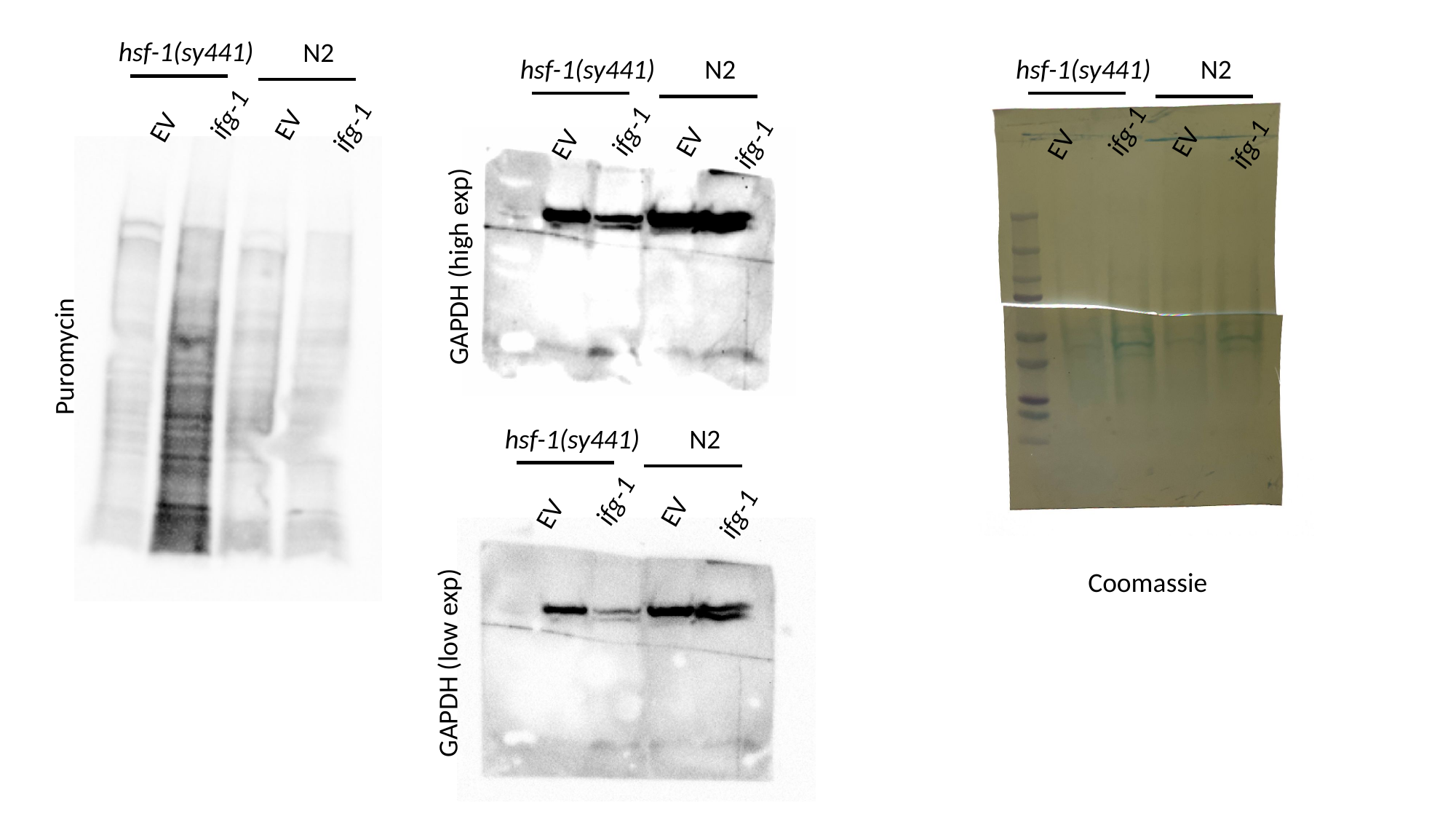

hsf-1(sy441)
N2
hsf-1(sy441)
hsf-1(sy441)
N2
N2
ifg-1
EV
EV
ifg-1
ifg-1
ifg-1
EV
EV
EV
ifg-1
EV
ifg-1
GAPDH (high exp)
Puromycin
hsf-1(sy441)
N2
ifg-1
EV
EV
ifg-1
Coomassie
GAPDH (low exp)

## Slide 2
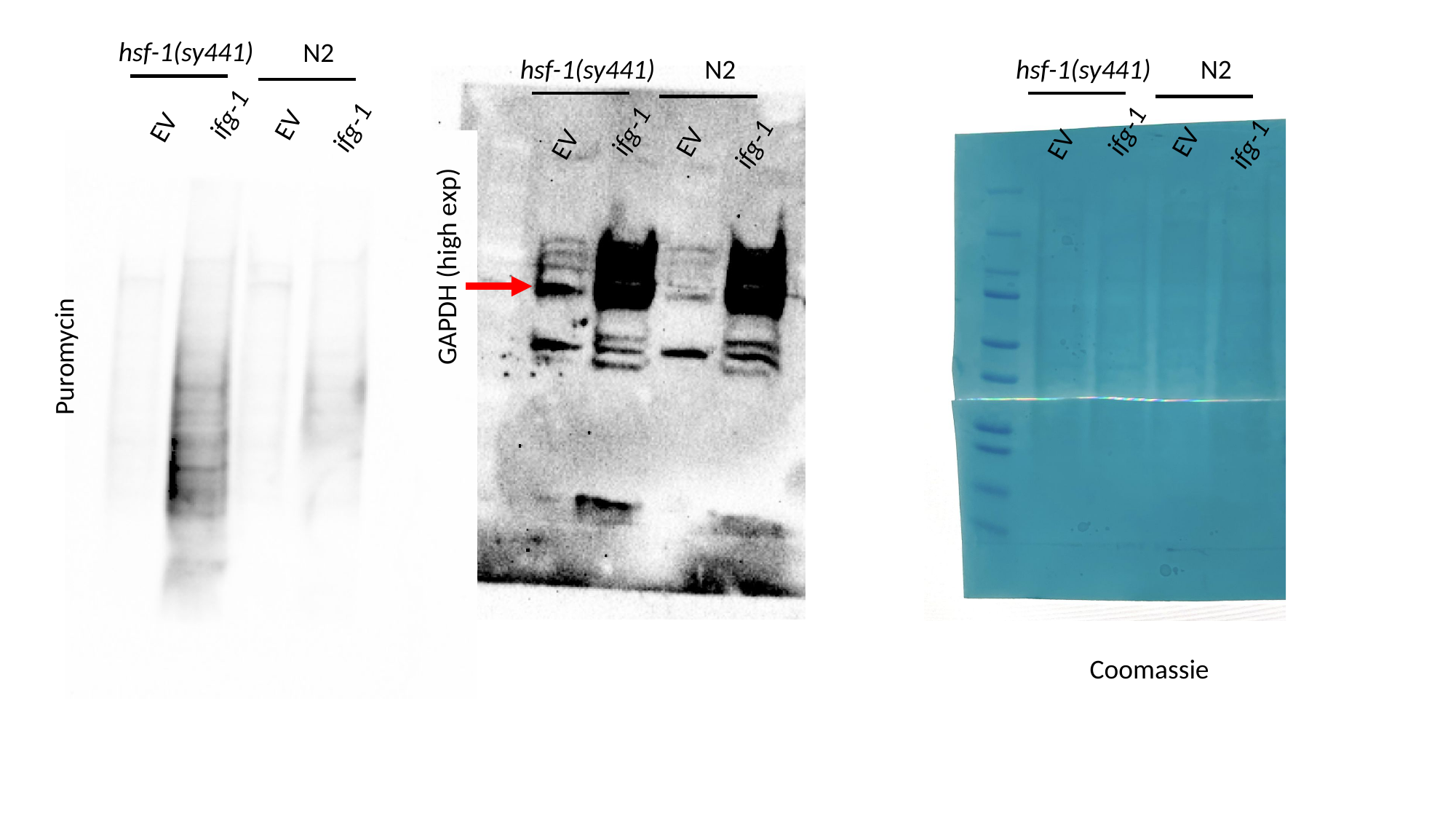

hsf-1(sy441)
N2
hsf-1(sy441)
hsf-1(sy441)
N2
N2
ifg-1
EV
EV
ifg-1
ifg-1
ifg-1
EV
EV
EV
ifg-1
EV
ifg-1
GAPDH (high exp)
Puromycin
Coomassie

## Slide 3
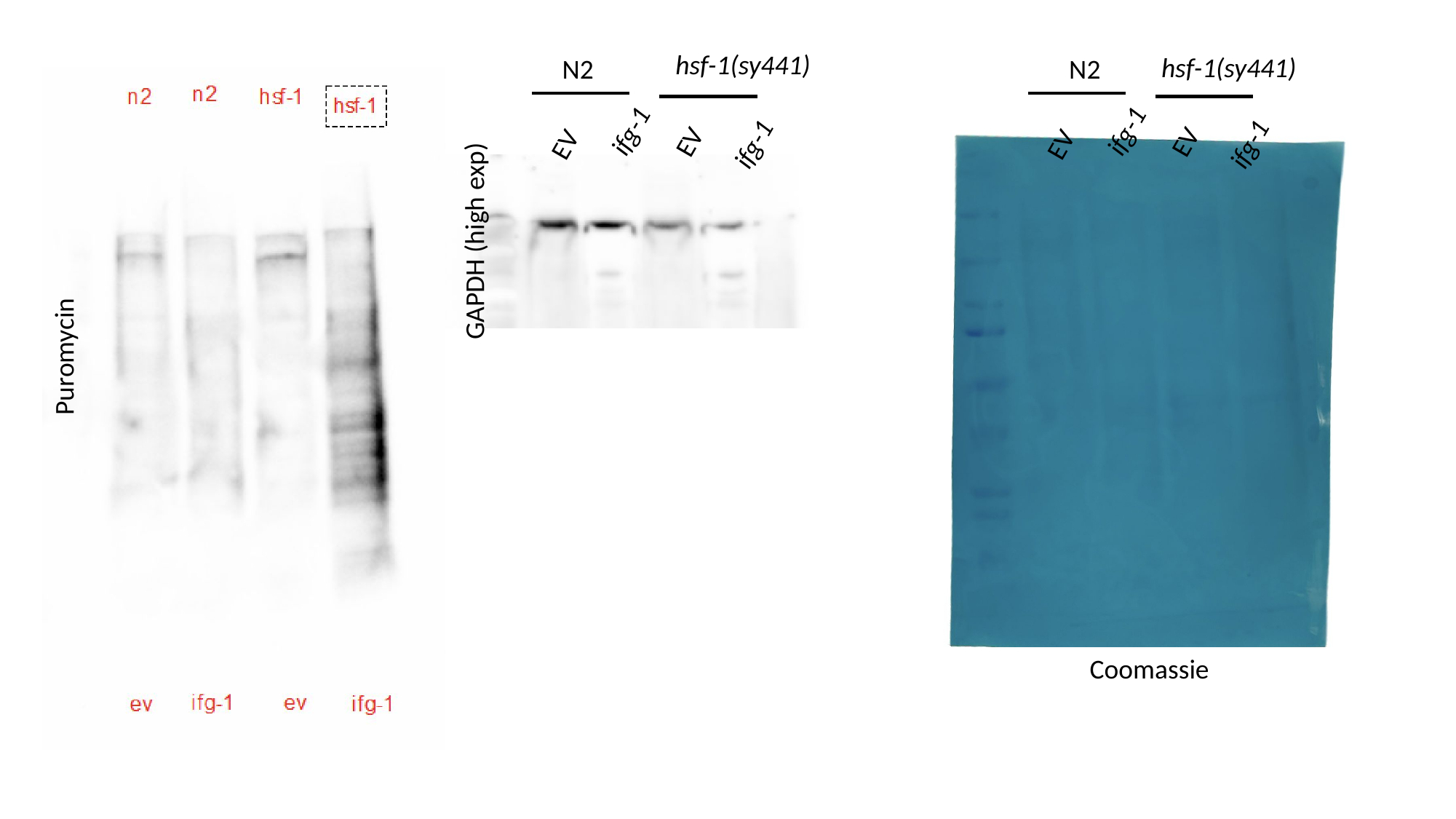

hsf-1(sy441)
hsf-1(sy441)
N2
N2
ifg-1
ifg-1
EV
EV
EV
ifg-1
EV
ifg-1
GAPDH (high exp)
Puromycin
Coomassie
